# Molecular Electrostatic Surface Potential: A Predictive Framework for Noncovalent Interactions and Adsorption Characteristics in Molecular Entities

**DOI:** 10.3390/ijms27083352

**Published:** 2026-04-08

**Authors:** Pradeep R. Varadwaj, Helder M. Marques, Arpita Varadwaj, Ireneusz Grabowski, Koichi Yamashita

**Affiliations:** 1 Institute of Physics, Faculty of Physics, Astronomy & Informatics, Nicolaus Copernicus University, 87-100 Toruń, Poland; 2 Molecular Sciences Institute, School of Chemistry, University of the Witwatersrand, Johannesburg 2050, South Africa; 3 Institute of Advanced Studies, Nicolaus Copernicus University in Toruń, 87-100 Toruń, Poland; 4 Department of Chemical System Engineering, School of Engineering, The University of Tokyo, 7-3-1, Tokyo 113-8656, Japan

**Keywords:** noncovalent interactions, σ-hole and π-hole interactions, molecular electrostatic potential, crystal engineering and catalysis, energy decomposition analysis, structure–property relationships

## Abstract

The molecular electrostatic surface potential (MESP) has become a key theoretical tool for probing reactivity in chemical systems. It reveals electrophilic and nucleophilic regions on molecular surfaces, underpinning the understanding of noncovalent interactions such as hydrogen, triel, tetrel, pnictogen, chalcogen, halogen, matere, and aerogen bonding, among many others. These interactions, driven by Coulombic attraction, govern aggregation in molecular and supramolecular systems across solid, liquid, and gas phases. MESP applications span crystal engineering, polymers, biology, catalysis, photovoltaics, and drug discovery. While limitations exist—such as the arbitrariness in defining isodensity surfaces—its impact on advancing both theoretical and applied chemical research is substantial. This review outlines the conceptual foundations of MESP and highlights its broad relevance across the chemical sciences.

## 1. Introduction

Chemical reactivity shapes bond formation and dissociation and is fundamental to the characterization and understanding of noncovalent interactions in chemistry [1,2,3]. This phenomenon pervades every corner of chemistry—be it the precision of chemical engineering, the innovation of medicinal chemistry [4,5], materials science [6], or supramolecular structures [7] and biological systems [8]. It governs the delicate assembly of molecules, from the nuanced folding of polymer chains to the creation of cross-linked network of thermoset adhesives [9], imbuing them with functionality and purpose [7]. The influence of this reactivity extends beyond the mere distribution of electron clouds; it is the subtle interplay between electrons and nuclei that shapes the behavior and properties of atoms and molecules, guiding their reactivity and unlocking their potential [10].

To truly grasp the essence of any chemical reaction, one must delve into the fundamental forces that draw specific sites on the surface of a chemical system toward others, whether within the same molecule or on neighboring molecules. This attraction sparks the breaking and making of chemical bonds, birthing new molecular species with distinct physical and chemical identities [11,12]. Take, for instance, the fascinating realm of ammonia catalysis [13,14,15,16]: here, the formidable triple bond of N_2_ succumbs to a complete disintegration when it meets the surface of a metal catalyst like Fe(111) [17,18]. This rupture is no mere accident; it is governed by a significant transfer of charge from the metal surface to the π* anti-bonding orbital of N_2_, an active space receptive to electron density. Under precisely tuned conditions of temperature, pressure, and the presence of hydrogen, the nitrogen sites adsorbed onto the metal surface are ignited into reactivity. They engage in a delicate interplay with molecular or atomically adsorbed hydrogen atoms, culminating in the synthesis of ammonia—a compound whose impact resonates profoundly in the advancement of society [19].

This review examines the advantages and limitations of the molecular electrostatic surface potential (MESP) model [20]. The model is widely applied in chemistry to quantify electrophilic and nucleophilic regions on molecular surfaces, providing a detailed characterization of molecular reactivity [11,21,22]. It has been applied across a range of chemical disciplines, including photocatalysis, catalysis, lithium-ion batteries, photovoltaics, crystal engineering, and anion recognition. Its use has also been extended to analyze hydridicity, redox potential, cyclo-carbon aromaticity, trans influence, N-heterocycle CO_2_ affinity, anion H_2_ affinity, and aspects of Clar’s sextet theory [23,24]. These studies collectively underscore the model’s application, akin to a master key unlocking the secrets of chemical interactions. Furthermore, this review reveals that when one calculates the electrostatic potential of a molecular surface, the deployment of a higher isodensity envelope may be recommended over the conventional 0.001 a.u. (electrons bohr^−3^) isodensity envelope. The limitations of the model are also addressed, particularly its challenges in accurately representing certain intermolecular and intramolecular interactions, providing a comprehensive assessment of its capabilities and constraints.

## 2. The Conceptual Framework of the Molecular Electrostatic Surface Potential Model

The MESP model’s versatility spans many scientific realms [11]—from the chemistry of small molecules [25,26,27,28] to the profound depths of delocalized systems [29,30,31,32], crystalline materials [33,34], and metal clusters [12,21], even extending its applicability to catalytic surfaces [21,35]. Its influence extends further, advancing the frontiers of medicinal chemistry [4,36,37], biology [38,39,40] and the art of drug design and development [41,42,43,44]. Through its lens, we gain a fundamental understanding of the directional characteristics that govern a dazzling array of noncovalent interactions within and between molecular entities in the crystalline phase, including, but by no means limited to, the delicate understanding of hydrogen bonding [45], halogen bonding [46], chalcogen bonding [47,48,49,50,51,52] pnictogen bonding [53,54,55,56] and tetrel bonding [57,58]. The model’s reach into the molecular interactions responsible for the liquid phase, though less frequent, still showcases its profound applicability and impact [59,60,61].

MESP is a physical property that is experimentally observable [62,63]. It can be determined by diffraction methods [64,65], and measured computationally [32,54,62,63]. The MESP, symbolically represented by *V(r)*, due to nuclei and electrons at a given point *r*, is defined as the work done in bringing a test positive charge from infinity to the reference point *r* (Equation (1)). The first and second terms on the right-hand side of Equation (1) are due to nuclei and electrons, respectively; *Z_A_* is the charge on nucleus A located at *R_A_*,* R_A_*−* r *and* r′ *−* r* are the distances between *r* and the nucleus or each electronic charge increment *ρ(r′)dr′*, respectively, and *ρ(r)* is the electronic density of the atomic or molecular entity.

(1) $$V\left(r\right)=\sum_{A}\frac{Z_{A}}{{|R}_{A}-r|}-\int \frac{\rho \left(r^{\prime }\right)dr^{\prime }}{\left|r^{\prime }-r\right|}$$

The MESP is often visualized by mapping the *V*(*r*) values on the molecular surface defined by an outer contour of *ρ(r)*. An isodensity surface, which may approximate the van der Waals surface of the molecular entity [61,66], is invoked since this assists in extricating lone pairs and π electron density, which are the essential features in the making and breaking of chemical interactions within or between molecular entities, and allows for the classification of closed-shell intermolecular interactions as σ-, *p*- and/or π-hole interactions [26,31,67,68,69], among others, including lump-hole interactions [22,70,71,72]. In particular, the potential on the 0.001 a.u. isodensity envelope is typically regarded as *V_S_(r)* [73]. When the first term on the right-hand side of Equation (1) dominates over the second term, the potential becomes positive and reflects the dominance of the nuclear contribution. When the second term on the right-hand side dominates over the first, the potential becomes negatively valued minima and saddle points associated with regions of high electron concentration. The maximum and minimum values of *V_S_(r)* are often designated as *V_S,max_* and *V_S,min_*, respectively. Each can be either positive, negative, or neutral, depending on where they show up on the surface, and the extent of electron density depletion or concentration. This is in contrast with the electron density that takes on only non-negative values [62]. The electrostatic potential can be expressed in units of energy (kcal mol^−1^, kJ mol^−1^, or eV).

## 3. The Molecular Electrostatic Surface Potential and Its Usefulness in Characterizing σ- and/or p/π-Holes in Molecular Entities

The descriptors emanating from the MESP model [74] must not lead to the assumption that positive electrostatic potentials are always associated with σ- and π-holes [75]. These regions of potential can exhibit nucleophilic behavior when the electron density is concentrated to some appreciable extent, yet they can also become highly electrophilic when marked by a significant depletion in electron density, enabling them to engage with electron-rich sites to form noncovalent interactions. The nature of σ- and *p*/π-holes, whether they manifest as positive or negative, is dependent on various factors, including the connectivity, electronegativity, and polarizability of the bonded atoms that shape the molecular entities and bonding regions [75].

### 3.1. The σ-Hole

An electron density deficient region does not always appear along the outer extension of the covalently bonded atom A in R–A, where R is the remainder part of the molecular entity. Whether the electrostatic potential on A in R–A is positive or negative depends on the electron-withdrawing character of R. This means that a σ-hole is not necessarily a region of positive electrostatic potential, as has been sometimes assumed [76,77]. When the σ-hole is observed on a transition metal complex, it is of a d-type since the d-orbital(s) is/are generally empty for low-coordinated transition metal atoms [12,21]. It is worth nothing that there is actually no physical hole on atom A in R–A. The term “σ-hole” literally refers to an “electron density deficiency” on the surface of bonded A, which appears along the outermost extension opposite to the R–A σ-bond.

**Figure 1:**
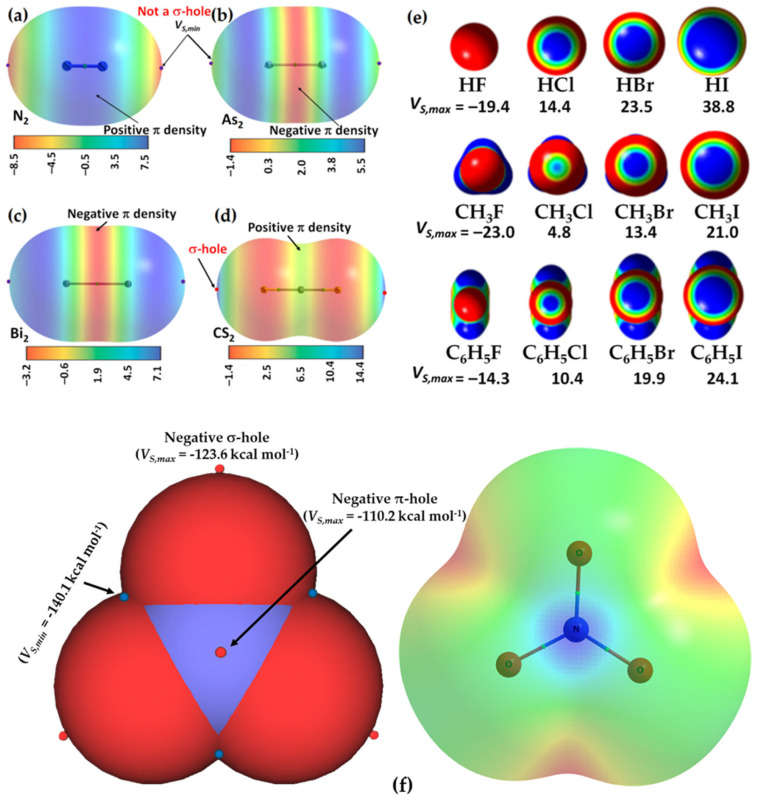
The 0.001 a.u. isodensity envelope mapped potential on the surfaces of some selected molecular entities. (**a**) N_2_ [MP2(full)/aug-cc-pVTZ]; (**b**) As_2_; (**c**) Bi_2_; (**d**) CS_2_. Shown in (**e**) are the [MP2/aug-cc-pVTZ-PP(Br,I)/aug-cc-pVTZ(C, H, F, Cl)]-level 0.002 a.u. isodensity mapped potentials of HX (top panel), C_5_H_5_X (middle panel), and CH_3_X (bottom panel) reported in ref. [78], where X = F, Cl, Br, and I. (**f**) The [M06-2X/Aug-CC-pVTZ]-level 0.001 a.u. isodensity mapped potential on the surface of nitrate anion (NO_3_^−^) obtained from this work. Values of *V_S_*_,*max*_ in (**a**–**e**) are in kcal mol^−1^. Selected *V_S_*_,*min*_ and *V_S_*_,*max*_ in (**a**–**e**) are shown as tiny circles in blue and red, respectively. The tiny, filled circles along bond extensions in panels (**a**–**d**,**f**), colored red (*V_S_*_,*max*_) and blue (*V_S_*_,*min*_), indicate the σ-hole regions, while the *p*/π-belt-like regions in (**a**–**d**,**f**) are highlighted with arrows. Colors of the surfaces: red indicates the most negative electrostatic potential, whereas blue indicates the most positive.

This region is not always associated with a maximum of potential, *V_S,max_*, and can also be associated with a potential minimum, *V_S,min_*; this may be referred to as a lump if it carries a negative potential, and an example is illustrated in Figure 1a, the MESP plot of N_2_ [56,73]. The outer extensions of the N≡N bond are not typified by *V_S,max_*, but by *V_S,min_*. The sign of *V_S,min_* is negative (*V_S,min_* = −8.5 kcal mol^−1^), showing an accumulation of electron density at the extension of the N≡N bond, which is less pronounced around the lateral portions of each N.

In the case of molecular phosphorous, P_2_, the *V_S,min_* on P along the P≡P bond extensions is positive (*V_S,min_* = 2.0 kcal mol^−1^), and more so around the lateral portions of the same atom (*V_S,max_* = 5.6 kcal mol^−1^), which may be regarded as a “hole”, as reported at the MP2(full)/aug-cc-pVTZ level of theory [54].

A very similar result was reported in the molecules As_2_ and Bi_2_ (see Figure 1b and c, respectively); the *V_S,min_* and *V_S,max_* values on As along and around the extensions of the As–As bond are 1.2 and 5.5 kcal mol^−1^, respectively, whereas those on Bi in the Bi–Bi bond are 5.0 and 7.1 kcal mol^−1^, respectively. The central bonding region features a belt of positive potential for N_2_ (*V_S,max_* = 7.6 kcal mol^−1^) and negative potential for P_2_ (*V_S,min_* = −1.7 kcal mol^−1^), As_2_ (*V_S,min_* = −1.4 kcal mol^−1^) and Bi_2_ (*V_S,min_* = −3.4 kcal mol^−1^). Clearly, none of these regions on the extensions of the pnictogen molecules can act as a σ-hole donor. One might characterize them as positive and negative *p*/π-belts, respectively. Clearly, these are unlikely to sustain a σ-hole interaction when close proximity is to either a negative or a positive site.

A σ-hole is an electron density deficient region along the outer extension of the covalently bonded atom A in R–A; of course, this deficiency is observed in reference to the lateral portion of the same atom A. It is characterized by a maximum of potential *V_S,max_*, which can either be positive (*V_S,max_* > 0), or negative (*V_S,max_* < 0), or neutral (when *V_S,max_* does not show up!). For instance, a σ-hole can be found on sulfur along each of the two C=S bond extensions in CS_2_ (*V_S,max_* = 14.4 kcal mol^−1^ each), Figure 1d, and is more positive than the lateral portion of the atom; the latter is described by a belt of negative potential (*V_S,min_* = −1.4 kcal mol^−1^ for each minimum). The electron-density distribution on the surface of the sulfur atom differs markedly from that in the central region of the molecule around the carbon atom, which is encircled by a belt of positive electrostatic potential (*V_S,max_* = 4.1 kcal mol^−1^). This feature explains why the C=S fragment in CS_2_ engages in noncovalent interactions with negative sites, leading to the formation of σ-hole-centered tetrel-bonded complexes with CO, HCCH, HCN, NH_3_, H_2_O, and H_2_S. In the case of CO, no σ-hole is present at the O end; instead, this region acts as a nucleophilic site that interacts with the tetrel holes of BH_2_ (B = Si, Ge, Sn, Pb). The resulting non σ-hole centered interaction strengths follow the order SiH_2_ > GeH_2_ > SnH_2_ > PbH_2_ [79].

The σ-hole on H in hydrogen fluoride is highly disperse (*s*-type) compared to that of F (*p*-type) in HF and of X (*p*-type) in X_2_ (X = halogen); details of the *s*- and *p*-type σ-holes on molecular systems may be found elsewhere [12]. Whereas regions on H and F that feature the σ-hole are both electron density deficient compared to the lateral portions on the respective atoms in the hydrogen fluoride molecule H–F [80,81], they have different character: the σ-hole on H is positive (electrophilic) and that on F is negative (nucleophilic, Figure 1e (upper)), but the nature of the surface potential and the positional occurrence of specific critical points of electrostatic potential can be tuned by spatial confinement as illustrated in a study by Lipkowski et al. [81]. Further examples of σ-holes that appear on A along the R–A bond extensions include the surfaces of the H and X atoms in hydrogen halide molecules (H–X), the X atoms in the dihalogen molecules X_2_, the halogen-substituted methane, and benzene derivatives, C_6_H_6-*n*_X*_n_* (*n* = 1–6, X = F, Cl, Br, I). They are characteristics of positive or negative *V_S,max_*, and some of them can be seen in Figure 1e, reported by Ibrahim et al. [78].

From Figure 1e, it is apparent that an increasingly positive σ-hole is often observed on some molecules containing higher halogen derivatives (*viz.* X in HX, CH_3_X, C_6_H_5_X, X = Cl, Br, I) and in C_6 × 6_ (X = Cl, Br, I). This is not the case in CX_4_ (X = F, Cl, Br, I) in which a positive σ-hole occurs regardless of the nature of the halogen derivative [82]. From Figure 1e, it is also clear that a negative σ-hole can be observed on F in the fluorinated molecules HF, CH_3_F and C_6_H_5_F. The same is true for the character of the σ-hole on the F atom in the series C_6_H*_n_*F_6-*n*_ (*n* = 1–6) [32], but not so on the halogen derivative in chemical systems such as HOX (X = F, Cl, Br) [83,84], and C in As(CH_3_)_3_ [55] and Bi(CH_3_)_3_ [85]. The σ-hole is neutral on the surface of the F atoms in chemical systems like AsF_3_ [55] and SbF_3_ [86].

The noncovalent interaction formed by the elements of a Group has been named based on the name of the Group of the periodic table. The “hydrogen bond” is an exception. When an electrophilic σ-hole is found on the surface of a bonded atom A of Groups 13 (triel derivative), 14 (tetrel derivative), 15 (pnictogen derivative), 16 (chalcogen derivative), 17 (halogen derivative), or 18 (noble gas atoms [67]), it can serve as a σ-hole-centered triel [57], tetrel [57,87], pnictogen [88,89], chalcogen [90], halogen [91], or aerogen bond donor [69,92] respectively. When it is found on the elements of groups 1 and 2 of the periodic table (except H), it is a σ-hole-centered alkali bond and alkaline earth bond donor [93], respectively. In similar vein, when it is found on the elements of Groups 11 and 12, it is called a σ-hole-centered regium bond and a spodium bond donor [94]. However, we have used terms such as “nitrogen bond” [56], “phosphorous bond” [54], “arsenic bond” [55], “stibium bond” [86], “bismuth bond” [95], and so on, even though they all belong to the same family of “pnictogen bonds”.

The term “refractory bond” [96] has been introduced by Varadwaj et al. to describe a newly defined family of σ-/π-hole interactions involving Group 4 transition metals—specifically Titanium (Ti), Zirconium (Zr), and Hafnium (Hf)—as electrophilic centers. The erythronium bond [97] involves Group 5 metals—vanadium (V), niobium (Nb), and tantalum (Ta)— can form directional Nb/V⋅⋅⋅O/N noncovalent contacts with electron-rich sites (O or N) in crystals. The term references vanadium’s historical name, erythronium, highlighting its electrophilic noncovalent interactions. A study has highlighted the role of matere bonds [98], which involve elements such as Mn, Tc, and Re from Group 7 [98]. Similarly, wolfium bonds involve elements of Group 6 in molecular entities [99,100]. These interactions are characterized by the metals acting as σ-hole donors, attracting electron-rich sites in close proximity, thereby facilitating effective molecular assembly. This concept is explored in this publication. We believe this is necessary because each element is unique in expressing its chemical reactivity and has bonding characteristics that are different from those of other elements of the same family. Similarly, Grabowski et al. [101,102] have pointed out that although Li and H belong to Group 1 of the periodic table, the definition of a hydrogen bond is not strictly valid for noncovalent bonds formed by Li. Zierkiewicz and coworkers [93] have referred to the noncovalent interactions formed by Group 1 as “alkali bonds”, which may be misleading unless H is explicitly excluded. Terms such as “lithium bond”, “beryllium bond” [103] and “magnesium bond” [104] have been used to recognize the noncovalent interactions formed by these atoms in molecular entities. Cavallo et al. [105] have proposed that intermolecular interactions should retain their distinct names based on the identity of the electrophile associated with a covalently bonded element of the periodic table, although this was recently criticized by Taylor [92].

Because σ-holes are defined by regions of positive electrostatic potential that typically occur along the outer extension of covalent bonds, one might ask whether the electrophilic region observed on the side of the pnictogen atom in P_2_, As_2_, and Bi_2_ (see above), characterized by *V_S,min_* > 0, could also be classified as a σ-hole. The answer is clearly no, since the defining criterion for a σ-hole is the presence of a positive electrostatic potential (*V_S,max_* > 0).

### 3.2. The π-Hole and π-Belt

A π-hole on a molecular surface can be negative or positive. Typically, it appears on the surface of delocalized bonds within the molecule or at the centroid region of the arene moiety. Bauzá et al. [106] suggested that the positive potential should be located on the vacant π* orbital of the molecular entity in order to it behave as a π-hole, as opposed to the σ-hole that might be associated with a high-energy σ* antibonding orbital.

The π-hole is negative (nucleophilic) around the centroid portions of C_6_H_6_ (*V_S,max_* = −16.9 kcal mol^−1^ with M06-2X/6-31+G(d,p) [107]), and positive (electrophilic) around the centroid portions of C_6_F_6_ (*V_S,max_* = +23.9 kcal mol^−1^ with M06-2X/6-31+G(d,p) [107]). It also appears on the outer surface of N in R–NO_2_ molecules [106,108]. It was argued that the charge distribution of in NO_3_^−^ is anisotropic, with a potential of −112 kcal mol^−1^ on nitrogen [109]. The molecule possesses *D*_3*h*_ symmetry, with a horizontal mirror plane that renders the electrostatic potential above and below the molecular plane equivalent. Our M06-2X/aug-cc-pVTZ calculations reveal a pair of strongly negative electrostatic potential extrema on the nitrogen atom (*V_S,max_* = −110.2 kcal mol^−1^), Figure 1f, indicating regions of high electron density. These features are analogous, in their location relative to the molecular framework, to the σ-holes observed along the outer extensions of the O atoms, but with the same sign (*V_S,max_* = −123.6 kcal mol^−1^). Consequently, nitrogen exhibits two π-type highly electron-rich regions rather than true positive π-holes. These regions are unable to engage in π-hole interactions with electron-rich sites due to dominant Coulombic repulsion at short distances. They can serve as electron donors for cations or interact favorably with arene moieties that exhibit σ- and π-holes, i.e., regions of appreciable electron deficiency. Of course this is contrast to certain systems, such as the CaCl_3_^−^ and SrCl_3_^−^ anions, that display a pair of positive *p*/π-holes on the surface of the alkaline-earth metal, enabling them to attract electron-rich sites effectively [110].

Nevertheless, it has been argued that the anisotropy of NO_3_^−^ is sufficiently dampened by resonance delocalization over a larger area, leading to the emergence of a weak Lewis acidic site on nitrogen that can interact favorably with electron-rich partners [109]. Such behavior is unlikely in the gas phase but may occur in the solid state, where NO_3_^−^ is significantly polarized and further polarizable, and where counterions play a crucial role in stabilizing the crystal lattice. Under these constrained conditions, the relatively low charge density and planar geometry of nitrate render the π-region sterically accessible, allowing NO_3_^−^ to act as a Lewis acid and form formally pseudo anti-electrostatic interactions in the solid state. There are many delocalized systems (including fullerenes, carbon nanotubes and allotropes of graphene) that do not feature σ-holes, but only π-holes, and they are either positive or negative by their character [57,111,112]. Examples may include,

A positive (or negative) π-hole is characterized by an area of electron density deficiency (or electron density rich) that is perpendicular to part of a molecular framework. In the context of double and triple bonds, π-holes refer to regions that are orthogonal to the direction of the covalent bond. The positive or negative nature of a π-hole can be explained by the sign of *V_S,max_*. Specifically, when a double or triple bond, or the centroid of an arene moiety, has *V_S,max_* > 0, it is termed an electrophilic π-hole; conversely, when *V_S,max_* < 0, it is referred to as a nucleophilic π-hole. For example, a negative π-hole can be observed on the central bonding regions of the C=C and C≡C bonds in H_2_C=CH_2_ [113] and HC≡CH [10,114], respectively, and a positive π-hole/belt can be observed in the bonding region in N≡N of N_2_ (Figure 1a). Both a σ-hole and a π-hole can also be found in non-aromatic molecules, such as KrOF_2_ and XeOF_2_ [93], and PnX_3_ (Pn = N, P, As, Sb, Bi; X = F, Cl, Br, I) [54,55,56,85,86]. The AClₙ^−^ anions, with central atoms A = Ca, Sr, Mg, Te, Sb, Hg, Zn, Ag, Ga, Ti, Sn, I, and B, constitute a family of chemical systems in which A exhibits a negative σ- or π-hole. Each anion interacts through its σ- or π-hole, even though the electrostatic potential associated with this hole is strongly negative in most cases [110]. Other studies focused on halogen-, pnictogen-, and tetrel-centered π-hole interactions may be found elsewhere [79,115,116].

**Figure 2:**
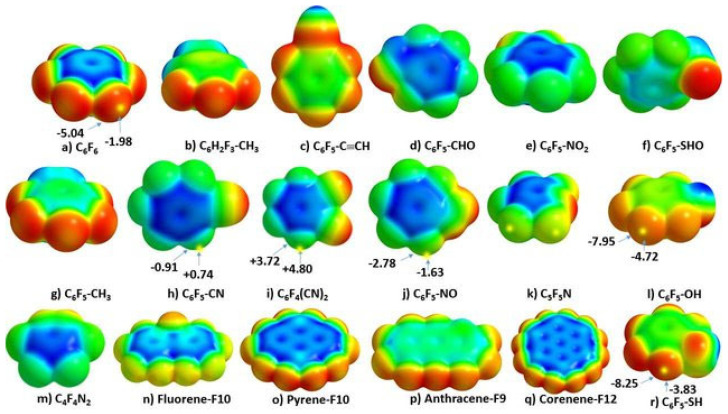
(**a**–**r**) PBE/6-311++G(2d,2p) level 0.001 a.u. isodensity mapped molecular electrostatic surface potential of some partially and fully fluorinated aromatic compounds, illustrating the presence of both σ- and π-holes. Regions colored red and blue refer to the most negative and most positive potentials, respectively. The maximum and minimum of potential (*V_S_*_,*max*_ and *V_S_*_,*min*_ (in kcal mol^−1^), respectively) are shown for some fluorine atoms in a few of these compounds, demonstrating the anisotropic nature of the charge density. For clarity, yellow dots representing *V_S_*_,*max*_ (negative and positive σ-holes) are shown only in a few cases. Reproduced from ref. [30].

Eighteen arene moieties with variety of functional groups are illustrated in Figure 2. The centroid regions (above and below the skeletal frameworks) of all these systems are positive to some degree. They represent positive π-holes. The σ-holes on the peripheral F atoms in these systems are either negative or positive. For instance, they are negative in C_6_F_6_ (Figure 2a), C_5_F_5_-OH (Figure 2l) and C_6_F_6_-SH (Figure 2r), among others, that feature surfaces colored orange and red. Similarly, they are positive in C_6_F_5_-CHO (Figure 2d), C_6_F_5_-NO_2_ (Figure 2e), C_6_F_4_(CN_2_)_2_ (Figure 2i), among others, that have surfaces colored blue and/or green. Details of the nature of the σ- and π-holes in these systems are discussed elsewhere [30].

Murray et al. have demonstrated that a *positive* π-*hole* can interact in a highly directional manner with negative sites, e.g., the lone pairs of Lewis bases, and is the counterpart of a σ-hole [117]. They contended that the electrostatic interaction is a major driving force in π-hole bonding, and a gradation can be found from weakly noncovalent interactions to considerably stronger ones with a possible measure of coordinate covalency.

The names of noncovalent chemical interactions such as hydrogen bond, halogen bond, and chalcogen bond and their definitions and cartelistic features, which have been recommended by a IUPAC working group [45,118,119] emerged from the names of the atoms or Groups of the periodic table, but they were primarily devised for σ-hole centered interactions. None of these definition papers mentions whether they are applicable to π-hole-centered interactions as well. We suggest that when the π-orbitals on the chalcogen, halogen or pnictogen atom in a molecular entity (such as in N_2_, Figure 1a) are involved in forming a π-hole centered noncovalent interaction with an interacting negative site, they should be named accordingly based on the name of the element, array of similar elements, that hosts the π-hole. For example, when the π-belt region of an N_2_ molecule engages attractively with the lone-pair-rich axial region of another N_2_ molecule, this interaction should be referred to as a “*p*/π-belt centered pnictogen bond.” Such interactions are observed in the polymorphs of N_2_ within the crystalline phase [56].

**Figure 3:**
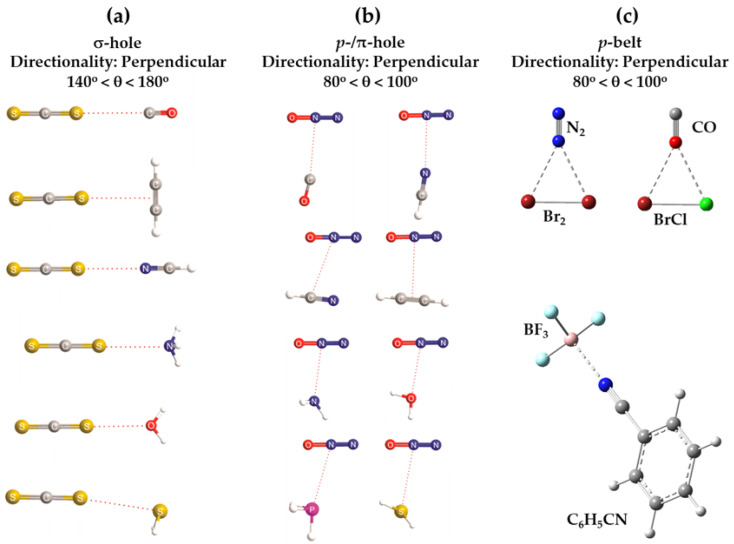
Schematic illustration of the directionality of σ-, π-, and *p*-hole interactions between an acidic site in one molecular entity and a basic site in another. Selected atoms in panels (**a**,**b**) are labeled as reported in [115], while the structures shown in (**c**) are fully reconstructed but remain representative of the systems investigated in [123,124,125]. Dotted lines denote intermolecular interactions.

The central bonding portion of a carbon monoxide molecule, C≡O, comprises a belt of positive potential (*V_S,max_* = 12.3 kcal mol^−1^ at PBE0/6‒311++G(d,p)) [120], and the axial portions feature a negative potential (*V_S,min_* (O=C) = −12.8 kcal mol^−1^ and *V_S,min_* (C=O) = −7.1 kcal mol^−1^). These results suggest that there is no σ-hole on this molecule. Kim et al. have described the anisotropy of charge density using a tripole model, comprising of two negative potential regions on the C and O atom-ends and one positive region around the cylindrical surface of the triple bond [121]. Interestingly, they found that when the spin state is changed to triplet, it features a significant dipole moment, with the O- and C-ends featuring negative and positive potentials, respectively. What should we call the chemical interaction formed by the π-belt of a singlet C≡O and the negative site of another CO molecule when they are in close proximity? This interaction arises because the π-belt in C≡O has orbital character from both O and C, with a notable contribution from the carbon atom. In this regard, the (C≡O)_π_···O interactions that contribute to various polymorphs of CO (space group *P*2_1_3, ICSD ref. 26,962 [122]) could be considered π-hole-centered tetrel bonds. Analogous intermolecular bonding features have been observed for interactions involving the central carbon atom in CO_2_ and the nitrogen atom in N_2_O with B = CO, HCCH, HCN, NH_3_, H_2_O, H_2_S, and PH_3_ (cf. Figure 3a,b). However, when CS_2_ is used as an acid, the sulfur end exhibits a positive σ-hole, resulting in linear complexes for all bases except H_2_O. The formation of tetrel and pnictogen bonds was demonstrated, with intermolecular distances ranging from 2.8 to 3.5 Å and bond dissociation energies between 0.71 and 3.57 kcal mol^−1^ (Table 1); notably, the tetrel bonds were comparatively stronger than the corresponding pnictogen bonds [115]. Comparable weak tetrel-bonded complexes, with SiH_2_, GeH_2_, SnH_2_, and PbH_2_ acting as π-hole donors toward bases such as N_2_, HCN, CO, and C_6_H_6_, have been reported by Chen et al. [79], exhibiting intermolecular bond distances and interaction energies that span from weakly to moderately strong complexes (Table 2).

### 3.3. The p-Hole and p-Belt

In a manner akin to σ-holes and π-holes, the somewhat enigmatic *p*-holes (or *p*-belts) found on the surface of an atom—or perhaps an entire ensemble of atoms—reveals itself through the maximum of potential, *V_S,max_*, comparatively devoid of electron density. The term appears to have originated from the work of Tarannam et al. [126] and Taylor [92] as they explored the electrostatic surface of boron trifluoride, BF_3_. Conventional wisdom, following, for example, the works of Bauzá et al. [106] and Zierkiewicz et al. [93], would suggest that the surface of boron in BF_3_ features a pair of π-holes, equivalent to that of S in SO_3_ [123], but these workers assert that “*p*-hole” is the correct designation, since its origins are the void in a *p*-type orbital. This notion perhaps extends to the entire BX_3_ (X = Cl, Br, I) series. We suggest that these *p*-holes on BX_3_ and SO_3_ can form a triel bond and a chalcogen bond with a base, respectively, as demonstrated by Zhang and coworkers [123].

In the same light, we may reconsider the conventional description of the triple bond of N_2_, which assumes it to be a region of π-electron density. In fact, there is a ring or belt of positive potential originating from an empty *p*-type anti-bonding orbital. Recent investigations have unveiled that even the hypervalent halogen atoms, in compounds such as XY_3_ (where X and Y are the halogens), feature π-holes on their surfaces [116,124]. These π-holes, born from *p*-type orbitals, are the very definition of *p*/π-holes, and they have an aptitude to engage in noncovalent interactions with nucleophilic regions on a molecular species with which they interact.

The dihalogens X_2_, along with their mixed derivatives, XY, also have *p*/π-belts around and perpendicular to the X-Y covalent single bond [124]. These belts of positive potential have a reasonable affinity to engage in noncovalent interactions. These *p*/π-belts form halogen bonds are partly the determinants of many crystal structures deposited in the CSD [127,128,129]. Ramaswami and Murray [130] have recently illuminated the intriguing role of “*p*-holes” in specific singlet halonium ions, drawing on previous research that revealed significant anisotropies in their electronic densities and electrostatic potentials. These *p*-holes engage favorably with nitrogen and oxygen bases, as well as other electron-rich sites, along the axis of X^+^. This axis features two distinctly positive local surface maxima (*V_S,max_*) that are symmetrically situated around the nucleus, enhancing their interactions.

## 4. The Molecular Electrostatic Surface Potential and Its Relationship with Directionality and Interaction Energy

A directional interaction follows a Type-II pattern of chemical bonding topology, in which the angle of interaction is either linear, or quasi-linear, or perhaps bent, where the interacting regions on the surface of the atoms that engage in the interaction have opposite charge polarity [82,131]. A number of studies have shown that the positive *V_S,max_* associated with σ-holes [67,132,133] and π-holes [117] are roughly consistent with the directionality of the interaction they sustain with a negative site. (For *p*- or π-hole interactions [109], the Lewis base approaches perpendicular to the plane of the π-system or electron-deficient atom, with bond angles typically near 90°, rather than following the linear or quasi-linear Type-II geometry characteristic of σ-hole interactions (cf. Figure 3)). This occurs in complexes reported in the gas phase, as well as in the solid state, even though the presence of secondary interactions sometime plays a significant role in changing a quasi-linear interaction to a non-linear one. There has been a continuous debate whether or not the directionality of σ-hole [67,132,133] and π-hole [117] interactions is electrostatically driven [63,73], or arise from the non-negligible exchange repulsion term [134]. Wang and coworkers [135] have found a linear correlation between charge transfer energy and total interaction energy that partially accounts for the directionality of halogen bonds over hydrogen bonds; this led them to demonstrate the suitability of simple force fields in the simulation of large systems involving halogen bonds.

Murray and Politzer [63] have shown the significance of *V(**r**)* in relation to the interaction energy *E_int_* of molecular complex systems. For a molecule A with an electrostatic potential *V_A_(**r**)*, the interaction energy *E_int_* with a point charge Q at position ***R*** is given by *E_int_* = *QV_A_(**R**)*. The signs of *Q* and *V_A_(**R**)* dictate whether the interaction is attractive or repulsive. For instance, if *Q* or *V_A_(**R**)* possess opposite signs, then *E_int_* < 0 and the interaction is attractive. Similarly, if both *Q* and *V_A_(**R**)* possess the same sign, then *E_int_* > 0 and the interaction is repulsive. This concept can be applied to rationalize the nature of an intermolecular interaction of molecule A with a second molecule B, given that B is simply a collection of point charges (nuclei and electrons). Hence, surface regions of positive (or negative) electrostatic potential on A will interact attractively with the surface regions of negative (or positive) potential on B when they are in close proximity. When the sign of *E_int_* between A and B is negative (attractive, *E_int_* < 0), the interaction is exothermic at 0 K, but endothermic when *E_int_* > 0.

Other studies have sought linear correlations between *V_S,max_* associated with σ-holes in various series of molecules containing atoms of Groups 14–17 and their interaction energies *E_int_* with negative site(s) [136,137,138,139,140,141]. While such a correlation was found using the potential obtained using a 0.001 a.u. isodensity envelope, others have demonstrated that the potential obtained using the 0.002 a.u. isodensity envelope correlates reasonably well with interaction energy of noncovalent interactions in a variety of systems, as evidenced by a large correlation coefficient. For instance, Lange et al. [46] looked at a range of 30 nitrogen-bearing heterocycles, halogenated systematically by chlorine, bromine, or iodine, yielding 468 different ligands that were used to probe scaffold effects on halogen bonding strength. As a template interaction partner, they chose *N*-methylacetamide as the protein backbone and adduct formation energies were obtained at the MP2/TZVPP level of theory. They examined the correlation between *E_int_* and the *V_S,max_* obtained with isodensity values ranging from 0.001 a.u. to 0.050 a.u. Their results showed that the best overall fit using a third-order polynomial function (*R*^2^ = 0.99, RMSE = 0.562 kJ mol^−1^) with rather smooth transitions between all halogens was obtained for *V_S,max_* calculated using an isodensity surface calculated with a 0.014 a.u. isodensity envelope.

Stenlid and Brinck [21] have extended the concept of the σ-hole to metallic systems (specifically, Au clusters), where they observed that the high reactivity at low-coordinated Au atom sites towards Lewis bases such as those in CO or H_2_O is a result of σ-holes, arising due to the overlap of mainly the valence *s*-orbitals. A linear correlation was found between the *E_int_* of the complex system for the binding of CO or H_2_O on Au-clusters and the *V*_S,max_.

Some studies [63,137,140] have shown that for two series of Group 14–17 molecules with their σ-holes interacting with NH_3_ and with HCN, the *R*^2^ values for the correlation between *E_int_* and the *V_S,max_* were 0.95 and 0.98, respectively. If different negative sites are involved, then their *V_S,min_* must also be taken into account via a double regression analysis (Equation (2)). This was discussed for 39 complexes involving Groups 14–17 plus hydrogen and six different negative sites, for which the predicted vs. computed *E_int_* had *R*^2^ = 0.91, despite the varied nature of the dataset [137]. If the negative site or the positive site was the same for all of the interactions, then it would suffice to include only either the *V_S,max_* or the *V_S,min_* of its partners [107]. A dual-descriptor combination was also demonstrated by others for different systems [142], where the strength of interactions between TiO_2_ nanoparticles ((TiO_2_)*_n_*, *n* = 7–10) and Lewis bases was shown to be predictable using Equation (2), which involves the *V*_S,max_ of the particle and the *V_S,_*_min_ of the base (viz., H_2_O, H_2_S, NH_3_, and CO).

(2) $$E_{int}=aV_{S,max}+bV_{S,min}+c$$

Storer and Hunter [61] observed that a striking correlation can be found when one attempts to connect *V_S,max_* and *V_S,min_* calculated on the 0.002 a.u. isodensity surface with the experimental noncovalent interaction parameters, *α* and *β*. These parameters quantify the relationship between chemical structure and the free energy change for the formation or exchange of hydrogen-bonded interactions. They describe the sum of all contributions to the interaction energy, i.e., changes in electrostatics, polarization, dispersion and entropy.

**Figure 4:**
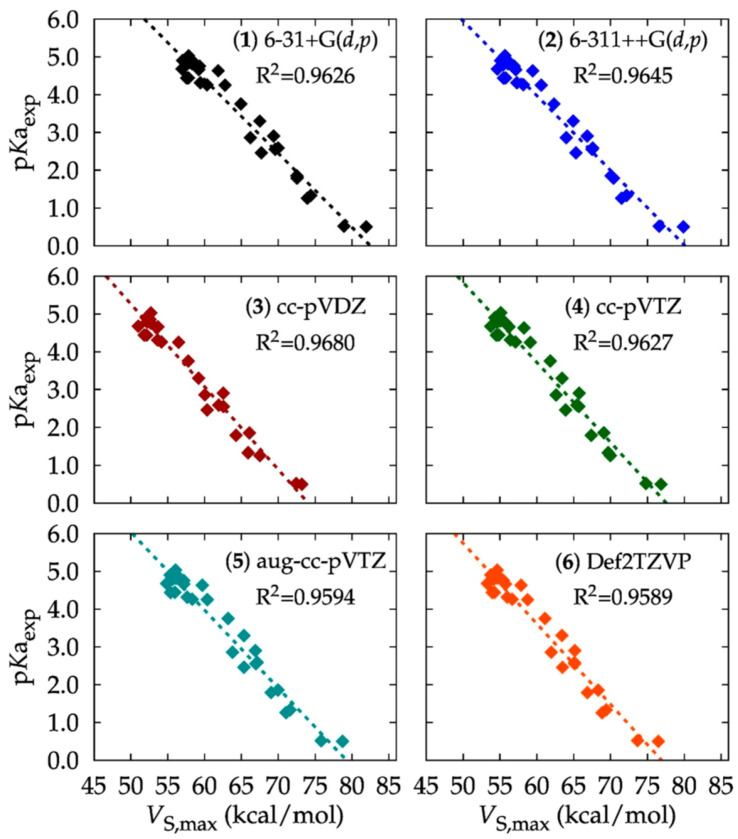
Illustration of the DFT-*ω*B97X-D level dependence of experimental pKa values on the local most maximum of potential, *V*_S,max,_ for some carboxylic based systems, obtained using six different basis sets. Reproduced from the work of Caballero-García et al. [143].

Caballero-García et al. [143] used the *V_S,max_* on the acidic hydrogen atoms of 30 carboxylic acids to describe the H-bond interaction with water and have correlated this with experimental p*K*a values to obtain a predictive model for other carboxylic acids. Among various levels of theory and basis sets applied, *ω*B97X-D outperformed in reproducing the reported *pKa* values, Figure 4. The best value of *R*^2^ was obtained with basis set cc-pVDZ, Figure 4(3), with a predictive power of 98%; the empirical relationship found was p*K*a = −0.2185 *V_S,max_* + 16.1879. Similar relationships have been discussed by Stenlid and co-workers [144]. Whereas the *V*_S,max_ calculated at the *ω*B97X-D/cc-pVDZ level of theory was found to be best to correlate with the reported p*K*a values, with a predictive power of 98%, their proposed descriptor was suggested to depend on the isodensity envelope used. The trends seen in Figure 4 was understood by examining the polarization of the O–H bond in the carboxylic acid structure. When the electron density of this bond is more polarized towards the oxygen atom, the hydrogen atom exhibits a more positive electrostatic potential. This increased positive character makes the hydrogen atom more labile and readily available for water to abstract, resulting in a lower p*K*_a_.

Sandhya and Suresh [145] have studied a series of metal hydride complexes of groups 6, 7 and 8 for water splitting reactions. Using the MESP approach, they found that the *V_min_* of the hydride ligand correlates with the MESP value at the hydride nucleus (*V_H_*). Moreover, the former correlates with the activation barrier for release of H_2_ and the latter with the binding energies of H···H bonded complexes. This confirms that *V_H_* serves as a useful indicator of the hydridic nature of the hydride ligand. The fluctuating electron-donating ability of the ligand environment manifests in the corresponding shifts in the negative character of both *V_min_* and *V_H_*. A more pronounced negative MESP leads to a lower activation energy for H_2_ elimination. Consequently, they have shown that the MESP characteristics offer a sophisticated method to fine-tune the ligand environment within a metal-hydride complex, optimizing the hydridicity of the hydride ligand. The efficacy of this MESP-based hydridic descriptor in orchestrating water-splitting reactions has been rigorously evaluated using model complexes of group 6 metal hydrides, specifically focusing on tungsten.

## 5. The Molecular Electrostatic Surface Potential Model and Intramolecular Interactions

The MESP model faces two principal limitations. First, it has limited ability to reveal intramolecular interactions within a molecular entity. This deficiency stems from its inability to identify local extrema of the electrostatic potential on atomic basin surfaces, which are essential for characterizing such interactions. These extrema—indicative of complementary positive and negative regions on interacting basins—are likely suppressed within the intramolecular bonding region. As a result, the model offers little insight into the physical chemistry of Coulombic interactions between these basins, despite their inherent presence.

Murray et al. [146] have asserted that when two formally nonbonded atoms A and B within a molecule come into close proximity, it raises the question: is this due to an attractive interaction, or is it merely a consequence of the molecule’s structure? Comparing the energy of the A–B interaction to the sum of the energies of A and B is not a reliable criterion, as it’s impossible to meaningfully determine the separate energies of A and B before their interaction when they are part of the same molecule. In this context, the geometric criterion of being “less than the sum of the van der Waals radii of the respective atomic basins” can be used to assess the reliability of whether the intramolecular interaction has actually occurred. This was shown for some chemical systems, including, for example, among others [147], in the optimized structures of H_3_Si–CH_2_–N(CH_3_)_2_ and ClH_2_Si–CH_2_–N(CH_3_)_2_, that feature Si···N noncovalent interaction.

**Figure 5:**
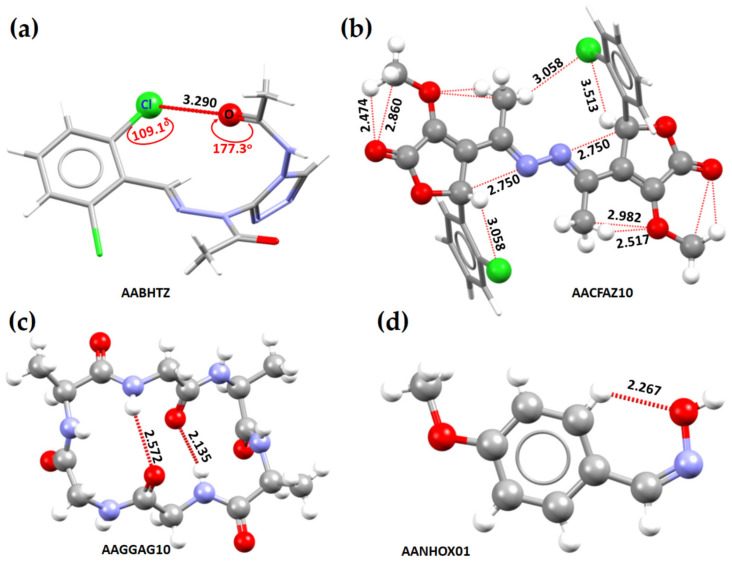
Illustration of intramolecular interactions in selected molecular entities reported in the crystalline phase: (**a**) 4-acetoamido-3-(1-acetyl-2-(2,6-dichlorobenzylidene)hydrazine)-1,2,4-triazole [148]; (**b**) *N,N’*-bis(3-acetyl-4-(2-chlorophenyl)-4-hydroxy-2-methoxycrotonic acid lactone)-azine [149]; (**c**) cyclo(*L*-alanyl-*L*-alanyl-glycyl-glycyl-*L*-alanyl-glycyl) [150] and (**d**) *Z*-4-methoxybenzaldoxime [151]. Selected bond distances and bond angles are in Å and degree, respectively. Atoms H, C, O, N, Cl in the ball-and-stick and mixed-crapped-stick models are colored in white, gray, red, blue and green, respectively. The CSD reference is shown in uppercase letters.

Let us now consider the chemical systems such as 4-acetoamido-3-(1-acetyl-2-(2,6-dichlorobenzylidene)hydrazine)-1,2,4-triazole [148], *N,N’*-bis(3-acetyl-4-(2-chlorophenyl)-4-hydroxy-2-methoxycrotonic acid lactone)-azine [149], cyclo(*L*-alanyl-*L*-alanyl-glycyl-glycyl-*L*-alanyl-glycyl) [150] and *Z*-4-methoxybenzaldoxime [151]. In the first system, Figure 5a, it is not readily apparent whether the Cl···O intramolecular interaction is the result of halogen bonding or chalcogen bonding. From chemical intuition, one would speculate that the O site double bonded with C in the C=O fragment may be nucleophilic, but it is unclear whether the lateral portion of the covalently bonded interacting atom Cl is electrophilic. Similarly, the nature of the close contacts C···N, C···O, H···Cl and H···O, identified in the crystal system in Figure 5b could be speculated upon based on the “less than the sum of the van der Waals radii “criterion. While chemical intuition might help identify the former two interactions as tetrel bonds and the latter two as hydrogen bonds, the MESP model falls short in assessing the origin and strength of these interactions. For instance, in the crystal depicted in Figure 5c, several hydrogen bonds of the H···O type are present (with two highlighted), whereas Figure 5d features two distinct types of hydrogen bonds (one exemplified as H···O). In both cases, the bonded atomic basins can be superimposed when viewed through a space-filling model. In such cases, analyzing atomic charges may offer insights into the coulombic attraction driving the interaction between these atomic basins.

**Figure 6:**
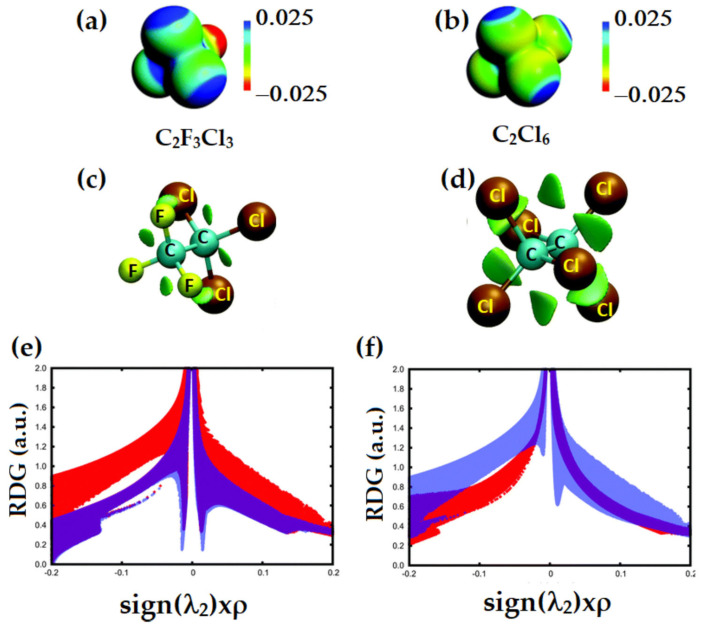
The MESP plot (in a.u.) of (**a**) C_2_Cl_3_F_3_, and (**b**) C_2_Cl_6_, mapped on the 0.001 a.u. isodensity envelope. Shown in (**c**,**d**) are the reduced-density-gradient (RDG) based noncovalent interaction (NCI) isosurface (green disks) plots in 3D for CF_3_Cl_3_ and C_2_Cl_6_, respectively, whereas that in (**e**) and (**f**) are the RDG-based NCI plots in 2D of CF_3_CCl_3_ (red) and C_2_Cl_6_ (translucent blue), and C_2_H_6_ (red) and C_2_F_6_ (translucent blue), respectively. Reproduced from the work of Johansson and Swart [152].

The DFT-revTPSS0-D level MESP for three perhalogenated ethane derivatives reported by Johansson and Swart [152] are shown in Figure 6a,b. The σ-holes (blue regions) were found on the surface the chlorine atoms along the C–Cl bond extensions in C_2_Cl_3_F_3_ and C_2_Cl_6_ (see Figure 6a and b, respectively). These electrophilic σ-holes play no role in the formation of halogen⋯halogen intramolecular interaction of the CX_3_–CX_3_ species, where X = halogen. This means that the X···X close contacts are not due to the involvement of a positive site attracting a negative site. The interaction isn’t “counterintuitive” either; rather, it arises from the overlap of the lateral sides of the halogen derivative, a distinctive feature of these molecules’ structure. They are characteristic of Type-I halogen bonds, in contrast to Type-II halogen bonds, which typically result from a σ-hole.

The reduced density gradient (RDG) isosurface volumes between the halogen atoms in CF_3_CCl_3_ and C_2_Cl_6_ confirm the attractive interactions between the halogen atoms in these molecules, which were not found in between the H atoms in C_2_H_6_ and that between the F atoms in C_2_F_6_. The spikes that emerged from the noncovalent-index (NCI) analysis for CF_3_CCl_3_ and C_2_Cl_6_, Figure 6e, are indicative of a van der Waals type interaction since they show up on either side of the sign(*λ*_2_) × *ρ* ≈ 0 region (Figure 6f), and were very different from what was calculated for C_2_H_6_ and C_2_F_6_. This is expected since the strength of the halogen⋯halogen interaction computed with DFT-D was +0.6, −18.8 and −22.2 kJ mol^−1^ for C_2_F_6_, CF_3_CCl_3_ and C_2_Cl_6_, respectively, indicating that it was repulsive in the first and attractive in the latter two species. Similar X···X intermolecular interactions were also observed in tetrel bonding environments in some other systems [58], and in halogenated benzene derivatives [29].

## 6. The Reliability of the 0.001 a.u. Isodensity Envelope

A second limitation of the MESP model is that the nature (both the sign and magnitude) of the potential depends on the isodensity envelope of the molecular entity used for mapping the potential. While the mathematical expression governing the expression of MESP, Equation (1), is unambiguous, the determination of the potential on the surface is somewhat arbitrary, which stems from the subjective nature of defining the isodensity envelope. For example, Baei and coworkers [153] have mapped the potential using a 0.004 a.u. isodensity envelope to provide the reaction chemistry of the phosgene molecule and its adsorption on Al_12_N_12_. Medrek and coworkers [154] have used the 0.004 a.u. isodensity envelope to provide the reactive chemistry of endohedral fullerenes, with endohedral species H_2_O, NH_3_, H_2_, 2H_2_, 3H_2_, 4H_2_, O_2_, and O_3_. Others have used a variety isodensity envelopes for exploring a variety of systems (for example, viz. 0.027 a.u. (Li et al. [84]); 0.01 a.u. (Popov et al. [155], Frontera [156]); 0.04 a.u. (Haakansson et al., [157]) and 0.05 a.u. (Omidi et al. [158])). Gatti et al. [75] investigated the surface of bonded sulfur along the C–S bond elongations in some chemical systems, but it did not find a σ hole when the potential was mapped onto an 0.001 a.u isodensity envelope. They therefore used isodensity envelopes in the 0.001 to 0.004 a.u. range to properly detect the small σ holes on S.

Some studies have used 0.002 a.u. isodensity envelopes to reveal the electrophilic and nucleophilic nature of many chemical systems, including fullerenes, carbon nanoring [11] cycloparaphenylene ([11]CPP) [159] and halide-substituted arene systems C_50_X_10_ (X = Cl and Br) [160]. There are also many studies that do not mention what isodensity envelopes were used to map the potential (see for example [161,162,163,164,165]). Clearly there is no consensus as to which isodensity envelope(s) should be used for the mapping of potentials.

Murray [166] studied how the 0.001 a.u. isodensity influences the electron density contours used to generate the electrostatic potentials of A···B complexes at equilibrium. She found that as the envelopes around A and B nearly touch and move closer to the nuclei, there is an optimal distance where the two envelopes merge into one, leading to the formation of σ-hole-bonded complexes. In either of the two cases, polarization of the electronic densities of partners A and B takes places. These observations led to a conclusion that the interactions between A and B begin beyond the van der Waals radii of the closest atoms, as demonstrated in several studies [167,168], and supports the notion that distances greater than 1.2 times the van der Waals radii of interacting atoms may indicate favorable interactions.

Politzer and coworkers have argued that the 0.001 a.u. isodensity envelope is an appropriate representation of the van der Waals surface, and therefore a suitable choice for mapping molecular electrostatic potentials [67,169,170], a view also shared by Scheiner [110]. As articulated by Murray and Politzer [171], although any molecular surface definition is inherently arbitrary, Bader et al. [66] proposed an outer contour of the electron density, ρ(***r***), which has the advantage of reflecting molecule-specific features such as lone pairs, π-electron density, and strained bonds. The 0.001 a.u. (electrons/bohr^3^) contour of ρ(***r***) was reported to encompass more than 95% of the total electronic charge. Subsequent work by the same authors suggested that similar isodensity surfaces may enclose up to ~97% of the electronic charge [172], while Bauzá and coworkers proposed that a slightly higher contour value of 0.002 a.u. encloses approximately 99.4% of the electron density [161]. Despite these quantitative differences, all studies trace their justification to the foundational work of Bader et al. [66], which broadly suggested that the 0.001–0.002 a.u. range captures roughly 95% of a molecule’s electronic charge. The reported fraction of enclosed electron density thus varies across studies [131,173], likely reflecting differences in molecular systems, computational methods, and interpretive criteria. The rationale for favoring substantially higher percentages, however, remains insufficiently articulated.

The selection of either a 0.001 or a 0.002 a.u. envelope depends on the nature and size of the atomic constituents in a molecule since these determine their overall volumes and polarities [66]. It is not surprising that each chemical system has a unique charge density distribution on its surface; so it may not be fully appropriate to always use the 0.001 a.u. isodensity envelope to approximate the van der Waals surface to map the potential of each and every chemical system [58].

The 0.001 a.u. envelope has failed on several occasions to reveal the correct nature of the potential of chemical system comprised of atoms with low polarizability. In particular, when a chemical system contains fluorine atoms, the model sometimes fails to provide the correct nature of the electrophilic character of covalently bound fluorine. For instance, when the 0.001 a.u. envelope was used to map the potential, the *V_S,max_* representing the σ-hole was absent on the electrostatic surface of F along Cl–F, Cl–F and S–F bond extensions in ClF_3_, ClF_2_Br and F_2_SO, respectively [174]. However, mapping the potential on the 0.0020 a.u. isodensity envelope provided a description of the strength and the complete nature of the electrostatic potential (and hence the reactivity of these molecules).

**Figure 7:**
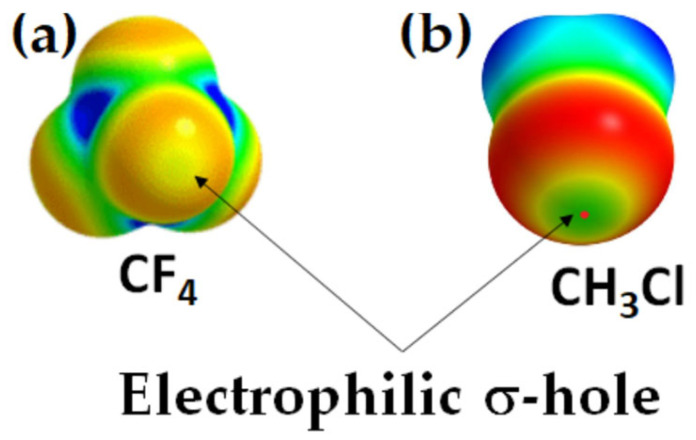
Illustration of electrophilic σ-hole on the surface of: (**a**) F in CF_4_ and (**b**) Cl in CH_3_Cl reported in [26,174] and [25,27], respectively. Colors of the surfaces: red indicates the most negative electrostatic potential, whereas blue indicates the most positive.

Consistent with Gatti and colleagues’ findings [75], we have also identified several chemical systems where the 0.001 a.u. isodensity envelope mapped electrostatic potential failed to accurately represent the true electrophilic and nucleophilic profiles. The insights derived from this envelope not only led to misleading interpretations of chemical reactivity but also mislead conclusions. Notably, Chloromethane (CH_3_Cl) and tetrafluoromethane (CF_4_) serve as key examples where the 0.001 a.u. isodensity envelope mapped potential misrepresents their true chemical behavior [25,26,27]. A higher electronic density envelope, greater than 0.001 a.u., is required for an accurate mapping of the potential, as illustrated in Figure 7, discussed elsewhere [25,26,27]. This discrepancy underscores the need for a more refined approach to the mapping of potential.

We found [25,26,27] that a strongly negative site, like O in H_2_C=O, is not always required to convert a negative potential on an interacting molecule into a positive one through its electric field [73,175,176]. For instance, it was shown that a nucleophilic σ-hole calculated on Cl in H_3_CCl can be transformed into an electrophilic σ-hole by the polarization induced by the electric field of an interacting H_2_C=O molecule [177]. However, this transformation is not essential to rationalize the realistic nature of the σ-hole on Cl in H_3_CCl, as the Cl atom inherently possesses an electrophilic σ-hole that enables it to interact effectively with negative sites, much like other organochlorine compounds [178]. Here, we used the term “σ-hole” instead of “potential” because the sign and magnitude of the potential indicate the presence and nature (positive or negative) of a σ-hole on an atom’s surface. If there is no maximum potential along this bond extension, then no σ-hole exists. It’s important to emphasize that an electrophilic σ-hole is essentially synonymous with a positive potential, as the latter defines the former along the covalently bond extension.

**Figure 8:**
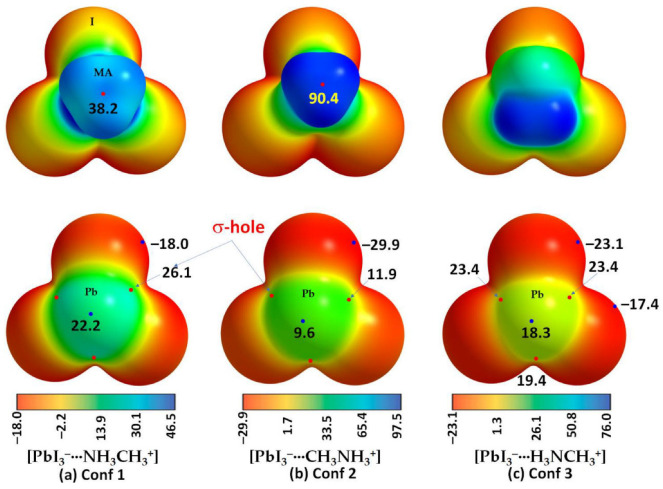
[*ω*B97X-D/def2-TZVPPD] level potential on the electrostatic surface of the three conformations of [CH_3_NH_3_^+^·PbI_3_^−^]: (**a**) [PbI_3_^−^···NH_3_CH_3_^+^]; (**b**) [PbI_3_^−^···CH_3_NH_3_^+^]; and (**c**) [PbI_3_^−^···H_3_NCH_3_^+^]. The methyl and ammonium groups in MA are facing the reader in the top panel of (**a**–**c**), respectively, in three different orientations. The Pb atom is facing the reader in the bottom panel of all three MESP plots shown in (**a**–**c**). Values on the color bar are in kcal mol^−1^. Atom labeling is shown for selected systems. Reproduced from the work of Varadwaj et al. [182].

The claim [73] that a negative σ-hole can be converted to a positive one induced by the electric field of an interacting partner may be applicable to other systems [25,26,27,179,180] but is not to H_3_CCl. For instance, a σ-hole on an atom in an anion (such as PbI_3_^–^) can be induced when it is in proximity to a cation (like CH_3_NH_3_^+^ or Cs^+^). This induction allows the anion to engage in intermolecular interactions with another same anion, contributing to the formation of an inorganic framework, as seen in halide perovskite semiconductors [181,182] and other ion-pair adducts [124]. This may be captured from Figure 8a–c, which deals with the [CH_3_NH_3_^+^·PbI_3_^−^] ion-pairs, in which the electrostatic potential on the Pb atom, initially negative in the PbI_3_^−^ anion, is entirely transformed or induced to become positive upon noncovalent interaction with the organic cation. This interaction drives the geometric formation of the CH_3_NH_3_PbI_3_ semiconductor in its crystalline phase, similar to other systems such as [Cs^+^•TtX_3_^−^] (Tt = Pb, Sn, Ge; X = I, Br, Cl [181]). This observation may contradict the assertion made by Kolář and colleagues [180]: “It must be stressed that the presence of a σ-hole is not induced by any interacting partner; it is solely a molecular property.”

Why a σ-hole is not found on the surface of F or Cl in CF_4_ and CH_3_Cl was previously explained as the result of the higher electronegativity of fluorine (or chlorine) that gives it a disproportionately large share of the σ bonding electrons, helping to neutralize the σ-hole [175]. It was further argued in another study that H_3_CCl and H_3_P can interact favorably with OCH_2_ and NSH even though the chlorine and the phosphorus holes have negative or near-neutral potentials; positive potentials were *induced* by the negative sites during the course of interaction [68].

These rationalizations are probably misleading because the σ-hole on F in CF_4_ and Cl in CH_3_Cl is not neutral, but positive (see Figure 7) on the extension of the C–F in CF_4_ and the C–Cl bond in CH_3_Cl [25,26,27]. Similarly, there is a negative σ-hole on F in CH_3_F; it is not neutral, as observed on other anionic systems discussed by Scheiner [110]. Although it is graphically invisible on CH_3_F, [67,183] its presence was quantified by a negative *V_S,max_* [183].

The misconception that the σ-hole on F in C_6_F_6_ [184,185,186] and CH_3_F is absent, or that it cannot be a negative, probably arose because of an incomplete understanding of the underlying concept of the σ-hole [175]. Such views are misleading, and invisibility of the σ-holes on F in these systems arose because the lone-pair regions around the lateral sites of bonded F atoms dominate [30], and the limitation of the color codes (e.g., AIMAll or MultiWfn) that is used to describe them.

**Figure 9:**
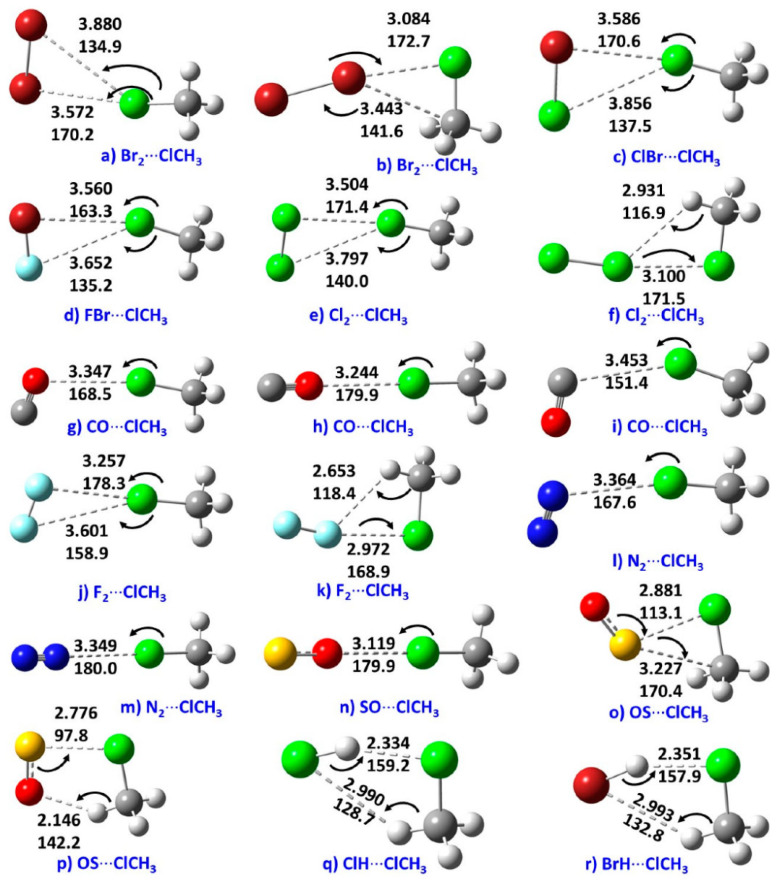
(**a**–**r**) MP2/aug-cc-pVTZ energy-minimized geometries of the 18 binary complexes of CH_3_Cl with a variety of donors [27]. The intermolecular distance in Å (upper entry) and the angle of approach in degree (lower entry) between the monomers in each complex are shown in each case. Reproduced from the work of Varadwaj et al. [27].

The electrophilic σ-holes on F and C in CF_4_ and CH_3_Cl explain why they attracted the negative sites in molecules with which they interact. This is illustrated in Figure 9a–r for complexes of CH_3_Cl with a variety of electron density donors. They form Type-II intermolecular halogen bonds when the axial portion of Cl along the C–Cl bond extension is engaged with the negative site on the partner species [27], even though the electrostatic field on the lateral side of Cl atom, for instance, in Cl_2_ is weaker than that on O in H_2_C=O [177]. Clearly, the majority of these shortcomings were the result of the use of an inappropriate isodensity envelope to map the potential, as well as an inappropriate basis set and/or correlation method [25,26,27].

## 7. Counterintuitive Interactions

Counterintuitive interactions, as discussed by Politzer and Murray [187] and others [107], refer to specific cases in molecular interactions that defy conventional expectations. These interactions often (but not always!) involve halogen bonds and σ-holes, other noncovalent interactions, where the anticipated behaviors based on electronegativity or traditional bonding theories may not hold true. For instance, a halogen may display unexpected electrophilic character due to the presence of a σ-hole, enabling it to engage in interactions with either the same or different molecular entities that aren’t immediately apparent based on its usual chemical properties. Similarly, an arene moiety might show unanticipated electrophilic or nucleophilic behavior, allowing it to interact with sites of similar charge capacities in ways that are not readily obvious. However, Clark et have pointed that Intermolecular attractive interaction between electrophilic sites is a counterintuitive phenomenon, even though the electrostatic interaction therein is repulsive [188]. These counterintuitive interactions highlight the complexity of molecular behavior and the importance of considering non-classical bonding concepts in understanding reactivity and stability in chemical systems.

Counterintuitive interactions may not be readily explained by the MESP model, although there are views on the contrary [187]. For instance, the model does not allow us to readily explain why an attraction between two nucleophilic sites [32,189,190,191,192] or between two electrophilic sites [174,193,194] can occur, for example, between halogen atoms in molecules, when they are in close proximity. Several reports of this have appeared. In some cases,[193,195] they were described as following a Type-III topology of bonding [55,86,189]. A Type-III interaction occurs between two interacting species when the interacting atoms have the same charge polarity and the angle of interaction is linear or quasilinear [54,55,82].

Murray et al. [187] have explored counterintuitive halogen bonding with nitrogen bases, where both the halogen σ-hole and the nitrogen lone pair exhibit negative potentials on their surfaces. Despite apparent repulsion between the ground-state molecules, these interactions were viewed as Coulombic, by explicitly considering electrostatics and polarization. Their analysis revealed that the energies of 20 counterintuitive interactions with four nitrogen bases can be effectively described using the electrostatic potential of the halogen σ-hole and the average polarizability of the nitrogen base. These two properties also effectively represented an expanded dataset that includes 20 additional weak and moderately strong intuitive halogen bonding interactions, where the σ-hole potentials are positive.

**Figure 10:**
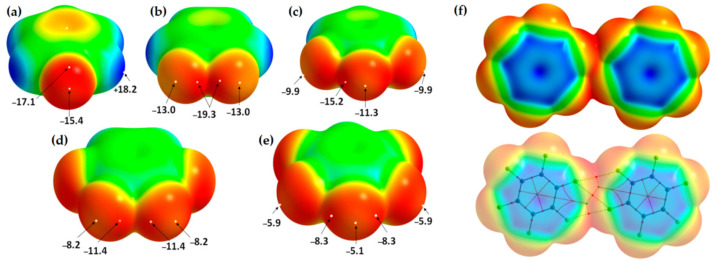
PBE/6-311++G(2d,2p) level 0.001 a.u. isodensity mapped MESP of some partially and fully fluorinated aromatic compounds: (**a**) C_6_H_5_F, (**b**) H_4_C_6_F_2_, (**c**) H_3_C_6_F_3_, (**d**) H_2_C_6_F_4_ and (**e**) HC_6_F_5_. The maximum and minimum (*V_s_*_,*max*_ and *V_s_*_,*min*_, kcal mol^−1^) of MESP are shown on selected fluorine atoms, showing anisotropy in the charge density. For clarity, yellow dots, representing *V_s_*_,*max*_ are shown for only a few cases. Shown in (**f**) is the MP2/cc-pVTZ level 0.001 a.u. isodensity mapped electrostatic potential of (C_6_F_6_)_2_: (**top**) solid fill; (**bottom**) stippled fill overlaying the QTAIM [66,201,202,203] molecular graph, with ring critical point to bond critical point connectivity shown. Colors of the surfaces: red indicates the most negative electrostatic potential, whereas blue indicates the most positive. Reproduced from [32].

Anti-electrostatic noncovalent interactions are stabilizing despite nominal electrostatic repulsion, arising from polarization, electron correlation, and other quantum effects. They occur in systems such as halogen bonds with negatively charged regions, anion–anion contacts, and unconventional hydrogen bonds, often involving σ-hole or π-hole bonding modes [196,197,198,199,200]. The MESP plots of several fluorine-substituted benzene derivatives are shown in Figure 10 (from [32]). Because the electrostatic surfaces of the F atoms in these systems are colored in red, this may lead to the interpretation that the σ-hole on F in these systems is neutral. The σ-hole on the F in each of these five systems is actually negative, becoming more positive as the number of F atoms increases in the series from Figure 10a through Figure 10e. This also applies in the case of hexafluorobenzene (C_6_F_6_), where each F has a nucleophilic σ-hole on its surface [32].

The values of *V_S,max_* associated with the fluorine’s σ-hole in H_5_C_6_F, H_4_C_6_F_2_, H_3_C_6_F_3_, H_2_C_6_F_4_ and HC_6_F_5_ were −15.4, −13.0, −11.3, −8.2 and −5.1 kcal mol^−1^ respectively at the [PBE/6-311++G(2d,2p)], level of theory [32]. In the case of C_6_F_6_, the lateral and axial regions of F along each C–F bond extension have *V_S,min_* = −5.8 kcal mol^−1^ and *V_S,max_* = −3.0 kcal mol^−1^ at the same level of theory. This shows that the σ-hole on F is somewhat more positive than those observed for the systems shown in Figure 10a–e and is not absent. In all cases, the lone-pair electron density residing on the lateral portions of F dominates, obscuring the σ-hole on F. Whilst F is negative in all these fluorinated benzene derivatives, it (except in the case of H_5_C_6_F) can enter into an attractive engagement with the equivalent atom in a similar molecule with which it interacts when in close proximity. This can be seen in Figure 10f, where the (C_6_F_6_)_2_ dimer has a binding energy of −0.42 kcal mol^−1^ with a stability that arises largely from the dispersive part of the interaction [32]. In fact, H_3_C_6_F_3_···F_3_C_6_H_3_, H_3_C_6_F_3_···F_4_C_6_H_2_, H_2_C_6_F_4_···F_4_C_6_H_2_, and H_2_C_6_F_4_···F_5_C_6_H exhibit anti-electrostatic behavior, where repulsive electrostatics (E (electrostatics) > 0) are overcome by induction and dispersion, yielding net attraction, while H_4_C_6_F_2_···F_2_C_6_H_4_ and H_4_C_6_F_2_···F_3_C_6_FH_3_ are slightly repulsive, and the highly fluorinated complexes (C_6_F_5_···F_5_C_6_H, HC_6_F_5_···F_6_C_6_, C_6_F_6_···F_6_C_6_) are stabilized primarily by dispersion with minimal anti-electrostatic character [32]. As Bayse commented [204], there are several studies that have reported a lack of completeness in the MESP model.

Zhao et al. [205] have demonstrated that two interacting negatively charged B_12_I_9_^–^ monoanions can not only attract, in apparent defiance of Coulomb’s law, but that the energy barrier at 400 K is small enough that they can combine to form a stable B_24_I_18_^2–^ moiety. This result was in line with the experimental observation of “spontaneous” formation of B_24_I_18_^2–^ in an ion trap. It was argued that this perhaps unexpected attraction between monoanions is due to competition between the attractive dipole–dipole interaction caused by the aspherical shape of the particle and the repulsive interaction between the like charges. In fact, this kind of attractive engagement between anionic moieties have been widely observed in the crystalline phase. An example [189] is the attraction between the [Cl_4_Pb]^2–^ anions that combine to form the inorganic layer in a variety of organic-inorganic perovskite systems (as in catena-[octakis(2,4,6-trimethylanilinium) bis(bis(*μ*-chloro)-dichloro-lead) bis(*μ*-chloro)-hexachloro-lead], materials (C_9_H_14_N)_2_PbCl_4_, [206]. In similar vein, the short O···O close contacts between ClO_4_^–^ anions, which have an entirely negative surface, observed in some crystals have been described as counterintuitive interactions [191]. The stability of these interactions is driven largely by dispersive forces and the MESP model may not be fully suitable to provide complete insight into the nature of these interactions.

Zhang et al. [207] have explored a number of fullerenes and carbon nanotubes using the MESP model. They argued that the large electrophilic regions on their electrostatic surfaces cannot be explained by the σ-hole or π-hole models. The authors calculated the MESP using the 0.0004 au isodensity envelopes for closed (5,5) C_160_, C_60_, C_80_ and C_100_ with a scale of −1.26 (red) to 1.26 (blue) kcal mol^−1^. According to them, it was the electron-withdrawing cavity that plays a key role for the formation of these large electrophilic regions on their surfaces. To support this argument, they have shown that the fullerene C_60_ has distinct electrophilic regions on the molecular surface, whereas C_50_H_10_, with the same curvature as C_60_, does not. This contradicts the general view that the high curvatures of fullerenes or carbon nanotubes lead to the large regions of positive potential on their outer surfaces, which may be reasonable given that the high curvatures of fullerenes or carbon nanotubes weaken the π-conjugation and hence decreases the electron density around the carbon rings of these systems. Our recent study [111] has provided a reasonable explanation of the surface reactivity of the fullerene derivatives using the MESP model, and its propensity towards the formation of tetrel bonds.

Holthoff et al. [200] have advanced the view that some crystals are stabilized by “anti-electrostatic” halogen bonds (AEXBs) between two anions, based on studies of 1,2-bis(dicyanomethylene)cyclopropanid moieties. Their explanation involved the importance of polarization and orbital interactions in halogen bonding, which may be useful for new classes of anionic XB donors. In this case, electrostatic interaction potentials were strictly negative (repulsive), a situation where the description of halogen bonding by static σ-holes will fail. Other similar studies [32,135,174,193,194,208,209] lead to an analogous conclusion, although in many of them dispersion played an imperative role in stabilizing the complexes. Predicting bonding interactions simply using “hole” concepts may not be enough [32,174,193,194], as other significant stabilizing factors may be important. As has been pointed out [210,211], the model clearly faces limitation when used to predict the strength of an interaction or a chemical bond, even though the descriptors of the model correlate with interaction energy, among other physical properties (*pK_a_* [143], dissociation energy, and force constants *k*_σ_ [115], etc.).

## 8. On the Applicability of the Molecular Electrostatic Surface Potential Model

The MESP model has found broad applications across fields such as 2D materials, liquid crystals, catalysis, energy devices, crystal engineering, and drug design [30,131,212,213,214,215,216]. Its versatility lies in providing mechanistic insight into molecular properties and interactions, making it a widely adopted tool for understanding structure–function relationships. What follows is a summary of its application in selected areas of particular research interest.

### 8.1. Photocatalysis

**Figure 11:**
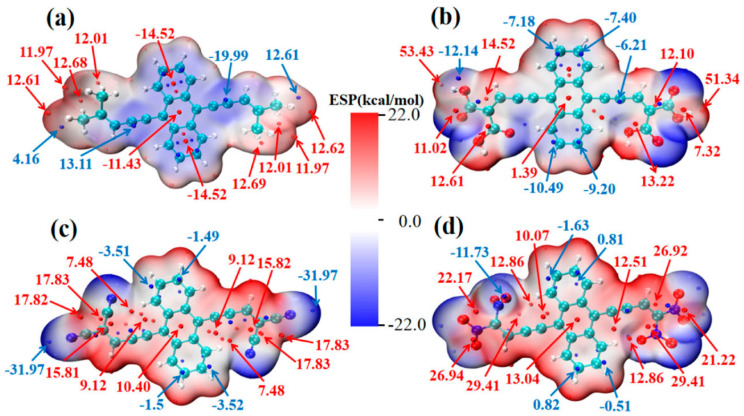
The molecular electrostatic potential vdW isosurface and its extreme points of (**a**) -H, (**b**) -COOH, (**c**) -CN, and (**d**) -NO_2_ conjugated molecular systems [217]. The red and blue regions represent the positive and negative electrostatic potential, respectively.

Li and coworkers [217] investigated the effect of substituents on conjugated anthracene–alkyne systems for photocatalysis. They demonstrated that substitution strongly influences the molecular dipole moment and second-order nonlinear optical (NLO) properties. Unsubstituted molecules exhibit a uniform electrostatic potential distribution (Figure 11a), resulting in small dipole moments and low NLO coefficients. In contrast, the carboxyl system exhibits the highest positive electrostatic potential (51.34–53.42 kcal mol^−1^, Figure 11b), the cyano system shows offset potentials near the substituent (Figure 11c), and the nitro system, with multiple potential maxima and a significant intramolecular electric field (Figure 11d), generates a long-range internal field. These variations in internal fields account for the observed differences in NLO responses and highlight the critical role of substituents in tuning photocatalytic properties.

**Figure 12:**
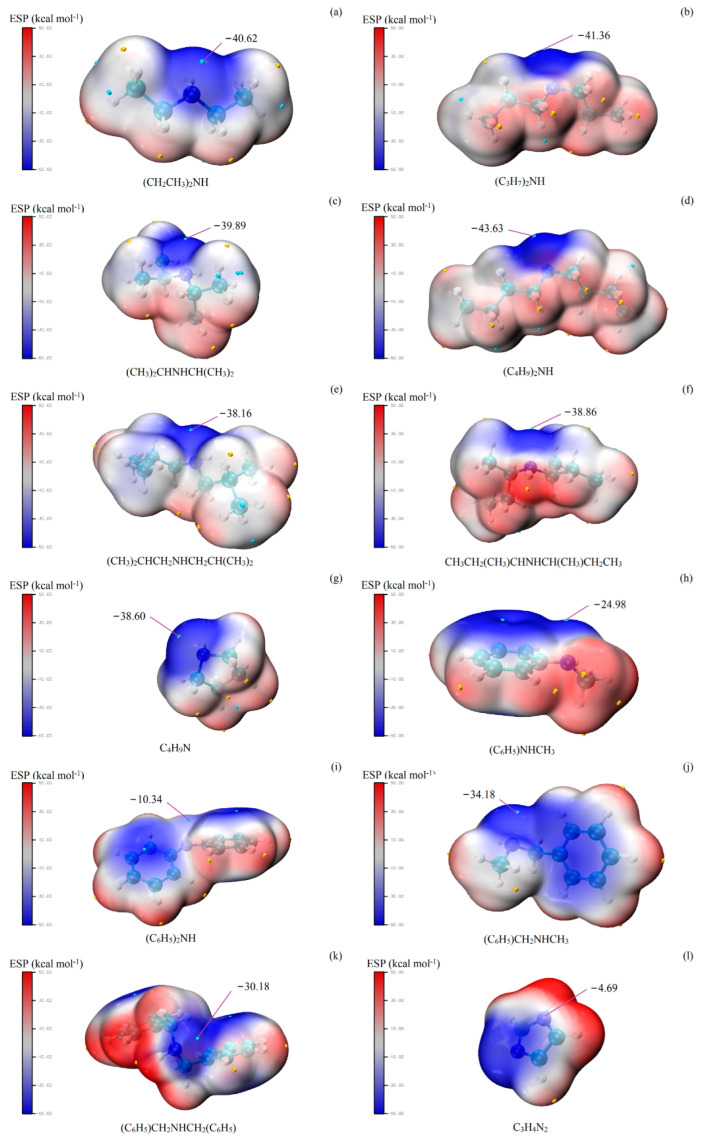
(**a**–**l**): Illustration of the electrostatic potential mapped with the molecular van der Waals surfaces of some amines, reported by Wang et al. [218]. The local minima of electrostatic surface potential (ESP) are marked in blue.

Methacrolein (MAL) is a key intermediate in the production of methyl methacrylate and other polymers. Wang et al. [218] used MESP to investigate how secondary amines and acids catalyze the condensation of formaldehyde and propionaldehyde into MAL. Their study revealed that catalytic activity depends on both the nucleophilicity of the amine nitrogen and steric accessibility, with acids serving as essential co-catalysts. At the M06-2X/def2-TZVP level (geometries from B3LYP/6-311+G(d,p)), the nitrogen atom was located near the global electrostatic potential minimum on the van der Waals surface (Figure 12a–l), reflecting its nucleophilic strength. Catalytic efficacy generally correlated with negative potential: imidazole, with the least negative potential, showed poor activity, whereas diethylamine, di-n-propylamine, di-n-butylamine, and pyrrolidine, with highly negative potentials, were highly effective. Steric hindrance limited the performance of diisopropylamine and di-sec-butylamine, and n-methylbenzylamine exhibited divergent behavior despite a potential similar to pyrrolidine. These results underscore the predictive power of MESP for rationalizing both electronic and steric contributions to catalytic performance.

The low concentration of photocatalytic alkaloids in plants presents a major challenge for rapid structural elucidation by spectral analysis. While single-crystal X-ray diffraction (XRD) remains the most precise technique, it requires crystallization, which is often time-consuming. To overcome this limitation, strategies such as the host–guest method, hydrogen-bonded frameworks, the crystal sponge approach, and DNA duplex sequences have been developed. Qin et al. [219] achieved a breakthrough by identifying a co-former that organizes these low-concentration molecules into highly ordered co-crystals, enabling rapid and accurate structural determination by XRD and revealing the stabilizing interactions. Among the co-formers, 1,5-naphthalenedisulfonic acid (NDS) proved particularly effective; its calculated electrostatic potential surface shows a pronounced negative charge on the 1,5-disulfonate groups, aligning with positively charged regions of the alkaloids. This complementarity confirms NDS’s efficacy as a co-former and highlights its utility as a powerful tool for structural analysis of complex photocatalytic alkaloid systems.

### 8.2. Catalysis

Gold nanoparticles are excellent catalysts to drive a variety of chemical reactions [21,35]. The catalytic activity is related to the binding affinity of the reactants on surface of the nanoparticle. Stenlid and Brinck [21] have shown that the high activity of Au clusters is associated with electron deficient (electrophilic) σ-holes on bonded Au atoms that are the binding sites for Lewis bases with the adsorption energy correlating with *V*_S,max_. For symmetric Au clusters of varying sizes, it was observed that the sites of most positive *V*_S,max_ are localized at the corners, edges and facets, with the electrophilicity the σ-hole decreasing in that order.

**Figure 13:**
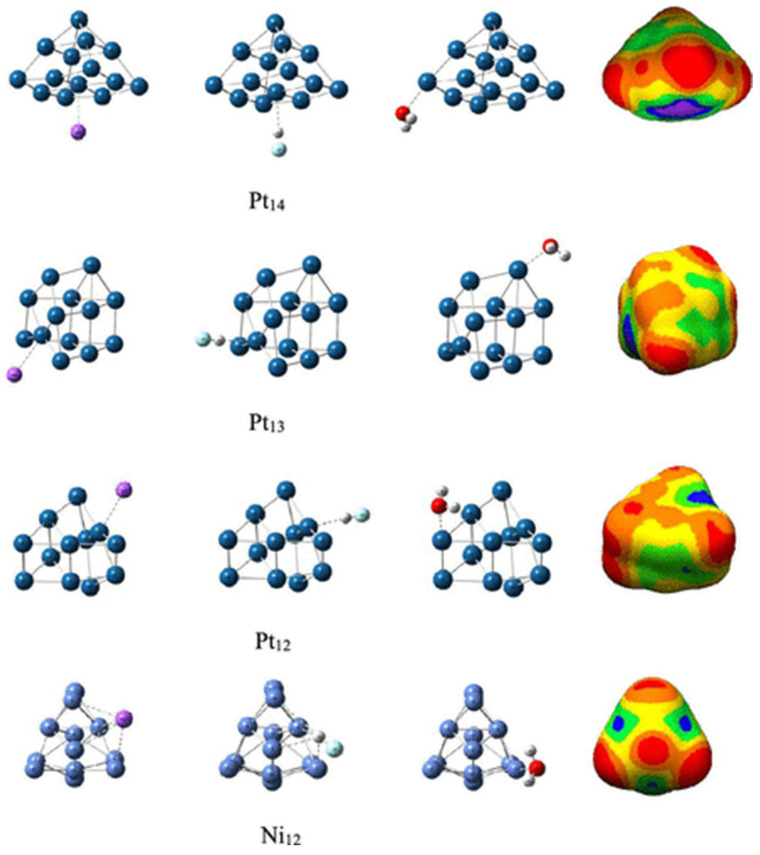
Illustration of the strongest binding sites of Na^+^, HF, and H_2_O with different nanoparticles (**left**), and the corresponding surface electrostatic potential maps of the bare nanoparticles from the same angle of view (**right**), reported by Li et al. [35].

Brinck and coworkers [35] examined the interaction of Pt_14_, Pt_13_, Pt_12_, and Ni_12_ nanoparticles with Lewis acids (Na^+^, HF) and a Lewis base (H_2_O) at the TPSSh/Def2-TZVP level of theory to provide an improved understanding of the interaction of transition metals with such species in order to facilitate the design of more efficient catalysts. The results obtained using the MESP model led to the interpretation that Pt and Ni nanoparticles are governed by σ_d_*-* and σ_s_*-*holes, respectively; the electrostatic potential surface maps of the bare nanoparticles are shown in Figure 13 (right). The lowest minimum (*V*_S,min_) and highest maximum (*V*_S,max_) of *V*_S_(**r**) on each particle can predict the most favorable binding site for the two Lewis acids and a Lewis base, respectively. In the case of H_2_O, the binding strength versus *V*_S,max_ correlation is better for Ni_12_ than for the Pt nanoparticles. The nature of interactions of these nanoparticles with Lewis acids and Lewis base is shown in Figure 13 (left). The binding energies for the nanoparticles with the interacting moieties varied between −0.25 eV (Pt_14_–HF) and −1.75 eV (Pt_14_–Na^+^).

Linear correlations were established between *V*_S,max_ or *V*_S,min_ with the interaction energy, Δ*E*_int_, for the complexes formed between the Pt and Ni nanoparticles with the two the Lewis acids and one Lewis base. Although some of the correlations had rather low values of *R*^2^, and others featured rather few data points, the authors found that the *V*_S,min_ of the Pt nanoparticles correlates well with Δ*E*_int_ for interactions with the Na^+^ and HF Lewis acids. This led them to demonstrate the importance of *V*_S,min_ for describing the activities of Pt nanoparticles with Na^+^ and HF. There is also a good correlation when Ni_12_ interacts with HF (H down), despite the fact that HF in many cases tilts over, yielding a relatively complex interaction mode with the surface. For the correlation of Ni_12_ with Na^+^, the *V*_S,min_ could locate the most active site but gave a correlation of lower quality. Concerning H_2_O, the *V*_S,max_ ranked well with the interaction sites of Ni_12_, which was not the case with Pt. In essence, it was concluded that the sites with the *V*_S,max_ and *V*_S,min_ of the largest magnitudes coincide with the strongest binding sites for H_2_O and Na^+^ (and HF), respectively.

**Figure 14:**
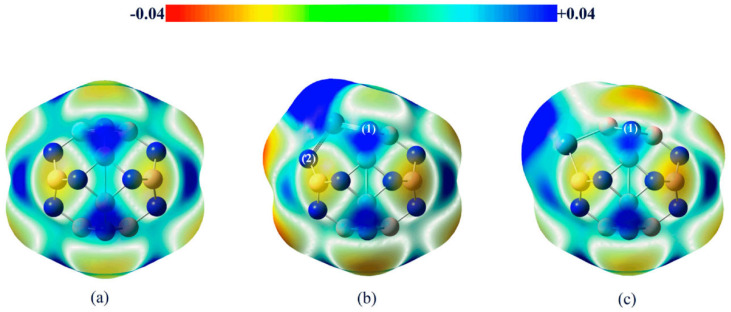
The electrostatic potential of: (**a**) B_12_N_12_, (**b**) B_11_N_12_Ni and (**c**) B_12_N_11_Ni clusters, mapped with on their respective 0.001 a.u. isodensity envelopes. (1) and (2) in (**b**,**c**) refer N, reported by Wang et al. [220].

Motivated by a wish to discover catalysts comprising non-previous metals, Wang et al. [220] examined the catalytic performances of two configurations of a boron nitride (BN) cluster doped with a single nickel atom. The MESP plots of undoped B_12_N_12_ and Ni-doped clusters (B_11_N_12_Ni and B_12_N_11_Ni) are shown in Figure 14. From Figure 14a, it is clear that the B and N atoms have positive and negative electrostatic potentials, respectively. In cases when Ni was doped (Figure 14b,c), the maximum and minimum of potential were observed around the Ni and N atoms which were positive and negative, respectively.

**Figure 15:**
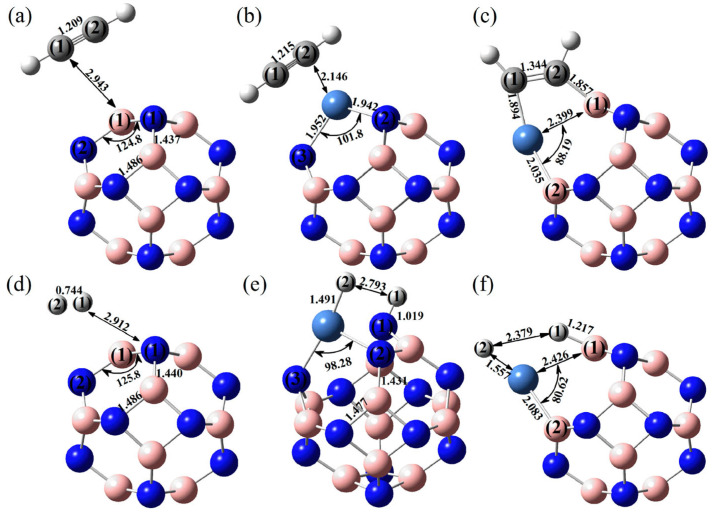
The optimized structures of C_2_H_2_ absorbed on B_12_N_12_ (**a**), B_11_N_12_Ni (**b**) and B_12_N_11_Ni (**c**); the optimized structures of H_2_ absorbed on B_12_N_12_ (**d**), B_11_N_12_Ni (**e**) and B_12_N_11_Ni (**f**). H atom is colored white; B in pink; C in grey; N bright blue; Ni in light blue [220]. Bond lengths and bond angles are in Å and degrees, respectively.

The optimized configuration shown in Figure 15a suggests that the acetylene molecule is adsorbed on the B_12_N_12_ cluster. However, the authors of the study argued there was no difference between the structures of acetylene and B_12_N_12_ clusters before and after adsorption. The adsorption of acetylene on B_12_N_12_ is physical in nature; at the [B3LYP/6-31+G(d,p)] level of theory, the adsorption energy *E*_d_ was found to be −2.83 kcal mol^−1^. This was not the case when Ni-doped B_12_N_12_ was used (Figure 15b,c)). In the first case (Figure 15b), the acetylene molecular was physically adsorbed on the B_11_N_12_Ni cluster with an [B3LYP/6-31+G(d,p)(H, N, B, C)/LANL2DZ(Ni)] *E*_d_ = −11.92 kcal mol^−1^, and the C≡C bond of acetylene was slightly elongated compared to that in free acetylene. In the second case (Figure 15c), adsorbed acetylene on the surface B_12_N_11_Ni was significantly deformed compared to its free linear geometry, with *E*_d_ = −40.40 kcal mol^−1^. This comparatively strong adsorption could be because both the Ni and B sites are very electrophilic, leading to the formation of Ni–C and B–C bonds.

The catalytic activity of B_12_N_12_, B_11_N_12_Ni and B_12_N_11_Ni towards molecular H_2_ was observed to be very similar (see Figure 15d–f). The HOMO-LUMO energy gap is reduced as a result of Ni doping, suggesting an increase in catalytic activity of the Ni doped clusters and hence signaling their suitability for the adsorption of molecular entities.

The activity of the B_11_N_12_Ni and B_12_N_11_Ni clusters differs by only 1.63 kcal mol^−1^, but the B_11_N_12_Ni cluster is expected to display higher ethylene product selectivity than the B_12_N_11_Ni cluster. This difference may be the result of the moderate degree of acetylene and hydrogen adsorption on the B_11_N_12_Ni cluster, based on the Sabatier Principle.

**Figure 16:**
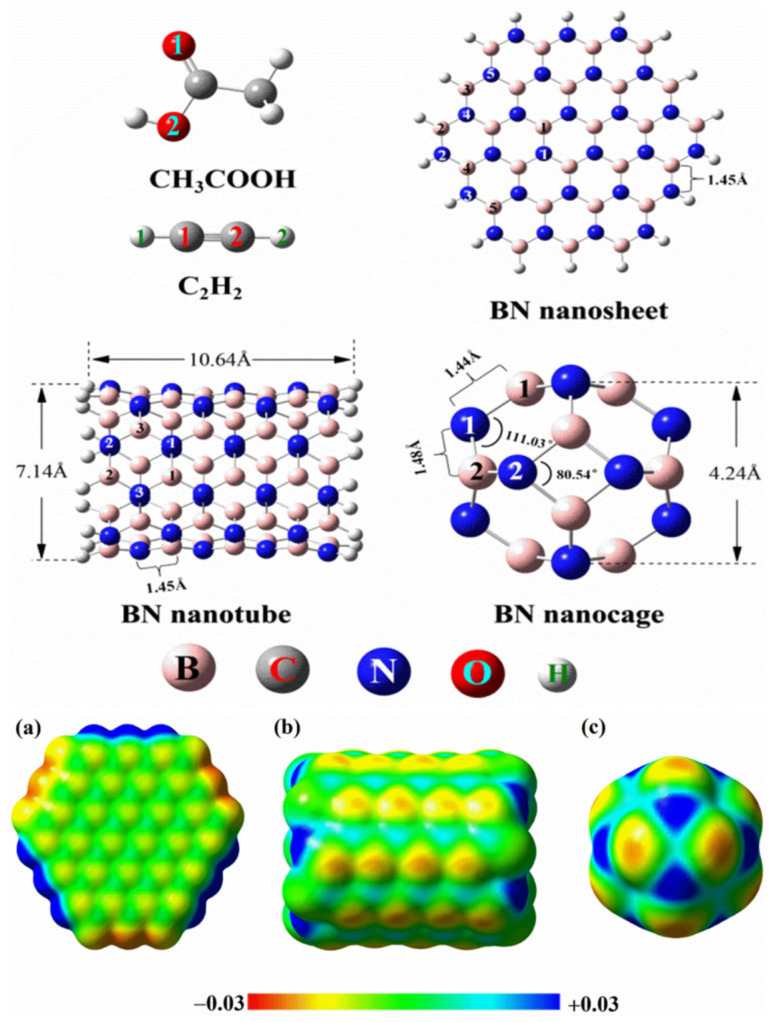
(**Top**) [B3LYP/6-31G(d,p)] level optimized structures of C_2_H_2_, CH_3_COOH and BN nanosheet, BN nanotube, and BN nanocage [221]. (**Bottom**) The 0.001 a.u. isodensity mapped potential on the molecular electrostatic surfaces of (**a**) a BN nanosheet, (**b**) BN nanotubes, and (**c**) a BN nanocage.

The catalytic reaction of acetylene and acetic acid with BN nanomaterials driven by an acetylene acetate reaction was examined by Zhang et al. [221]. The DFT-B3LYP relaxed structures of various chemical species involved in the study are shown in Figure 16 (Top). The MESP plots of the 2D sheet, tubular and cage structures of BN are illustrated in Figure 16a–c.

It is evident from Figure 16a–c that the electrostatic surfaces of the BN nanostructures are all different. The B sites in the nanocage structure (Figure 16c, blue regions) are significantly more electrophilic than the N sites. A similar conclusion about the surface of B and N may be reached for the tubular and sheet-like structures of BN, but the surface reactivity is strongest in the cage structure. This explains why the authors of the study [221] determined that the cage structure has superior catalytic performance.

**Figure 17:**
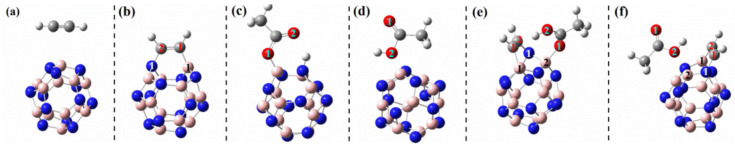
[B3LYP/6-31G(d,p)] level optimized adsorption structures of C_2_H_2_ and CH_3_COOH molecules on B_12_N_12_ nanocages. (**a**,**b**) are the single adsorption structures of C_2_H_2_ on B_12_N_12_. (**c**,**d**) are the single adsorption structures of CH_3_COOH on B_12_N_12_. (**e**,**f**) are the co-adsorption structures of C_2_H_2_ and CH_3_COOH on B_12_N_12_, respectively. Reproduced from ref. [221].

The *E*_d_ for the interaction between C_2_H_2_ and B_12_N_12_, Figure 17a, was endothermic (*E*_d_ = 5.32 kcal mol^−1^); this is typical of physical adsorption. By contrast, the configuration shown in Figure 17b was exothermic (*E*_d_ = −26.33 kcal mol^−1^). This is typical of chemical adsorption, in which, the C≡C (sp) triple bond of acetylene transformed into a C=C (sp^2^) double bond, thus activating the acetylene molecule. This configuration (Figure 17b) was not explored in the study of Wang et al. [220], so they arrived at a different conclusion about the surface reactivity of B_12_N_12_.

The nature of the adsorption of CH_3_COOH on the surface of the BN cage structure was very similar, with *E*_d_ = −19.43 kcal mol^−1^ and 6.27 kcal mol^−1^ for structures shown in Figure 17c and d, respectively. In the former, acetic acid is dissociated and adsorbed during the adsorption process. It did not dissociate in the latter case during the adsorption process, although the O(2) atom of the carboxyl group does interact with a B atom of the catalyst.

The values of *E*_d_ of acetylene and acetic acid on the cage structure were −24.28 kcal mol^−1^ (Figure 17e) and −17.10 kcal mol^−1^ (Figure 17f), respectively. Acetylene adsorbed in different ways on the (Figure 17e,f). The connection between C and B involved an electrophilic reaction with H^+^, whereas the connection to N entailed a nucleophilic reaction with CH_3_COO^−^. This is not surprising since the electrostatic potentials of C atoms bonded to B and N were negative and positive, respectively. The catalytic surface profile of the cage structure was stronger than that of the 1D tubular and 2D sheet structures of BN since these systems accommodated a physical adsorption of acetylene.

**Figure 18:**
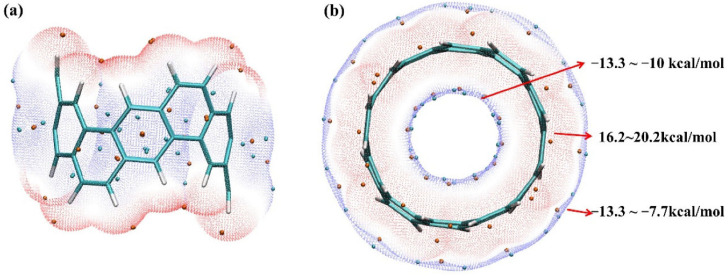
The [B3LYP/def2-SVP] level electrostatic potential of the (**a**) side and (**b**) top view of [6,6]-CNB, mapped on the 0.01 a.u. isodensity envelope [225]. The red (blue) regions represent the area where the electrostatic potential is positive (negative), and the tiny red (blue) spheres represent the maximum (minimum) of the electrostatic potential.

The catalytic activity of carbonaceous materials has been known since at least 1925 [222]. Carbonaceous nanomaterials are excellent catalytic materials thanks to their large specific surface area and many surface-active centers. These catalytic materials include fullerenes, graphene, carbon nanotubes and carbon nanodots [223,224]; the [6,6] carbon nanobelt [6,6]-CNB is an example and is composed of 12 edge-sharing rings. Lu et al. [225] designed a series of dimers of [6,6]-CNB combined with heterogeneous catalytic substrates (N_2_, CH_4_, CO, CO_2_, H_2_, H_2_O, H_2_O_2_, and O_2_) that are commonly used in the field of energy chemistry. The motivation was the view that a fundamental level understanding of interactions between catalysts and supports is of great significance for regulating catalytic active sites and their performance. The MESP of [6,6]-CNB is shown in Figure 18a,b. The distribution of potential on the electrostatic surface of the interior and exterior surfaces of the carbon framework of [6,6]-CNB were different. The extrema of potential on the interior carbon surface of the system range between −13.3 and −10.0 kcal mol^−1^, whereas that on the external carbon surface range between −13.3 and −7.7 kcal mol^−1^. The electrostatic potential on the surfaces of the hydrogen atoms ranged between 16.2 and 20.2 kcal mol^−1^, showing they are strongly electrophilic. However, authors of the study did not make it clear in a quantitative way what specific regions on the carbon surface of [6,6]-CNB feature the minima and maxima of potential. One might expect that the centroid regions of the arene rings feature potential maxima.

**Figure 19:**
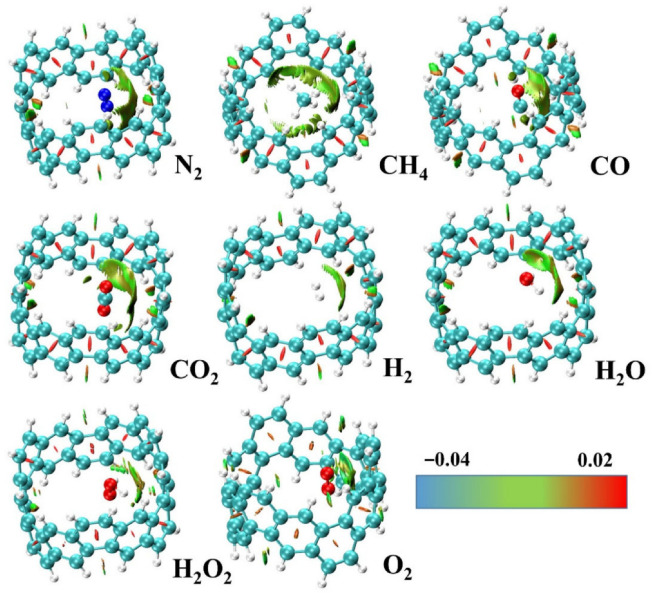
The RDG isosurface plot (isovalue = 0.5) of small molecules adsorbed on the interior surface of [6,6]-CNB. The color scheme highlights the attractive (blue) and repulsive (red) nature of interaction between the host carbon nanobelt and the guest small molecules [225].

Among the dimers formed between the exterior of [6,6]-CNB and the small molecules investigated, it was found that the adsorption of O_2_ led to the formation of a covalent bond that deformed the framework. The small molecules were also found to adsorb on the interior surface of [6,6]-CNB, but the moderate interaction energy (−2.0 < *E*_d_ < −8.5 kcal mol^−1^) was primarily due to a significantly stronger dispersion interaction. The reduced density gradient (RDG) plots of the absorbed molecules on the interior surface of [6,6]-CNB are shown in Figure 19. The conclusion of the study was that a basic understanding of the physical components of weak intermolecular interactions is crucial for the performance and selectivity of molecular catalysts.

**Figure 20:**
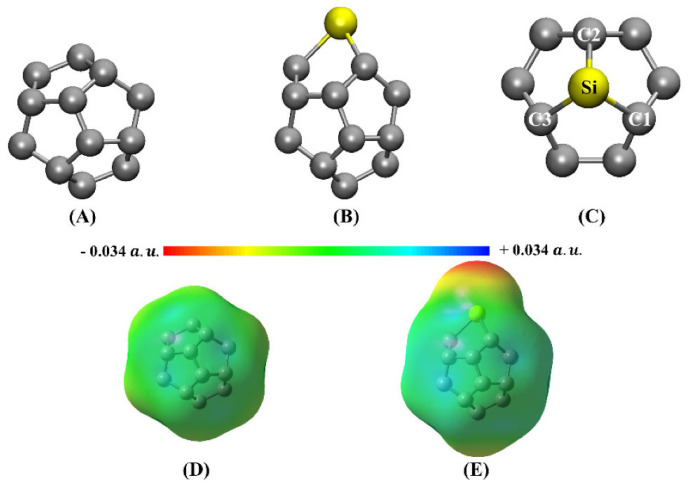
Illustration of the [*ω*B97XD/6-311+G(d,p)] optimized geometries of the (**A**) C_20_ and (**B**) C_19_Si fullerenes. Shown in (**C**) is the upper view perspective of the C_19_Si structure. The electrostatic potential on the surfaces of C_20_ and C_19_Si are shown in (**D**,**E**), respectively. Reproduced from ref. [226].

Lemos Silva et al. [226] replaced a C atom of fullerene C_20_ with Si (Figure 20A–C), and examined the nature of the adsorption of molecular CO, CO_2_ and N_2_ on C_20_ and C_19_Si. While C_20_ is non-polar, its Si-doped analog has a dipole moment of 1.950 D. The MESPs are given in Figure 20D and E, respectively, and show the very different nature of the surface of two species. Since the potential extrema on C_20_ molecule vary between −7.0 and 12.0 kcal mol^−1^ these should be discernible in Figure 20D; that they are not is probably because the same scaling was used to color both C_20_ and C_19_Si.

The adsorption of CO, CO_2_ and N_2_ onto C_20_ was unfavorable. However, an immediate result of the polarization of the carbon fullerene by doping with Si doping was that C_19_Si now favorably adsorb CO, CO_2_ and N_2_. C_19_Si significantly favored CO adsorption over that of CO_2_ or N_2_, and the increase of the electric field intensity (between 0.005 a.u. and 0.025 a.u.) resulted in the transformation of physical adsorption to a partial covalent bond formation for the C_19_Si-CO system. Although these were the concluding remarks of the study, it is not clear how the MESP plots enabled the authors to resolve the origin of the transformation from physical adsorption to one with partial covalent bond formation.

**Figure 21:**
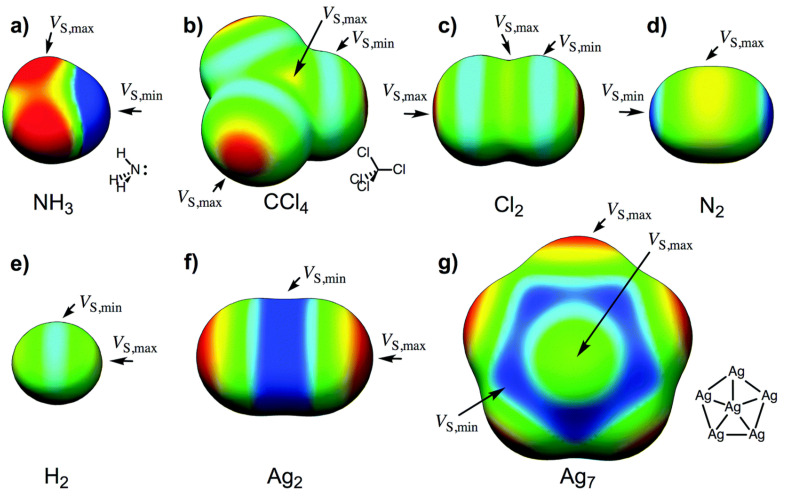
The electrostatic surface potential, *V*_S_(**r**), computed on the 0.001 a.u. isodensity surface for the (**a**) NH_3_, (**b**) CCl_4_, (**c**) Cl_2_, (**d**) N_2_, (**e**) H_2_, (**f**) Ag_2_, and (**g**) Ag_7_ compounds. Coloring from high to low potential, central values of the colors in eV in parentheses: red (0.8) > yellow (0.4) > green (0.0) > cyan (−0.2) > blue (0.4). Selected maxima and minima in the surface potential are marked as *V*_S,max_ (σ-hole) and *V*_S,min_ (σ-lump) respectively. Reproduced from ref. [22].

**Figure 22:**
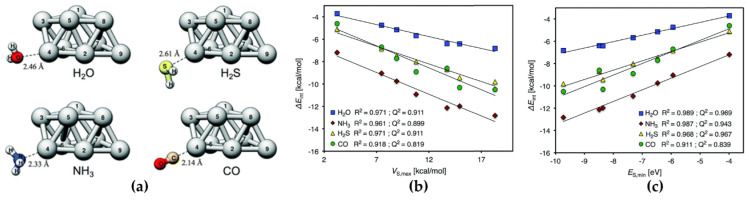
(**a**) The favored adsorption structures of H_2_O, H_2_S, NH_3_ and CO Lewis bases on Ag_9_, reported by Stenlid and coworkers [22]. Correlation between (**b**) *V*_S,max_ (top) and (**c**) *E*_S,min_ (bottom) with local interaction energies, Δ*Ε_int_*, of the H_2_O, NH_3_, H_2_S and CO electron donors at the seven unique sites of Ag_9_. *V*_S,max_ and *E*_S,min_ were evaluated on the 0.001 a.u. isodensity surface. *E*(**r**) represents the local electron accepting capability (Lewis acidity) of a compound and minima in *E*(**r**) on an isodensity surface, *E*_S,min_, correspond to Lewis acidic sites.

Stenlid and coworkers [22] have introduced and explored the concept of “regium bonds,” a type of noncovalent interaction between noble metal nanoparticles—copper, silver, and gold—and electron donors (CO, H_2_O, NH_3_ and H_2_S) and electron accepting entities (BH_3_, BF_3_, HCl (with H down) and Na^+^), resembling traditional noncovalent bonds like hydrogen or halogen bonds. Using density functional theory, they identified specific regions on these nanoparticles—known as σ-holes (electron-deficient) and σ-lumps (electron-rich), Figure 21a–g, which drive these interactions. Shown in Figure 22 are the structures of the Lewis bases H_2_O, H_2_S, NH_3_ and CO on Ag_9_. Plots of H_2_O and H_2_S interaction energies with the various sites on Au_9_, Ag_9_ and Cu_9_ vs. the site resolved *V*_S,max_ and *E*_S,min_ obtained at the 0.001 a.u. isosurface, gave a weak correlation that led to the conclusion that some interactions involve factors beyond purely electrostatic or charge-transfer/polarization controls. This is shown in Figure 22b,c for Ag_9_, although for this particular case values of *R*^2^ are reasonably high.

Other studies [144,227,228,229] have provided important insights into the reactivity of transition metal cluster surfaces [230], encompassing transition metal complexes [48,231], metalloporphyrins [232], nanocomposites [232], and Mn-, Fe-, Co-, or Cu-doped indium nitride nanocages [228]. Planar clusters such as Au_8_(PH_3_)_8_ and Au_8_(PH_3_)_4_-outer functions as reduction sites, donating electrons primarily from the phosphine ligands [227]. As the number of ligands coordinated to the gold atoms increases, the clusters’ ionization energy decreases accordingly. By contrast, Au_8_(PH_3_)_4_-inner displays a more limited negative electrostatic potential due to steric hindrance from out-of-plane ligands, which diminishes its capacity for molecular reduction. The high catalytic activity of gold nanoparticles is explained by the magnitude and location of σ-holes (corners > edges > facets)—positive surface potential regions at low-coordinated Au atoms—which serve as binding sites for Lewis bases, with binding strength correlating to *V_S,max_* and accounting for the observed increase in catalytic activity with decreasing particle size, a concept also applicable to other transition metal nanoparticles like platinum [21].

An N_2_ molecule consists of two nitrogen atoms bound by an exceptionally strong homonuclear triple bond. Each nitrogen atom has a pair of electrons in the 2s orbital with opposite spins and three lone-pair electrons in the 2p orbitals with parallel spins. The s-p orbital hybridization results in four bonding orbitals (two σ and two π orbitals) and four antibonding orbitals (two σ* and two π* orbitals), with the electrons shared in the π and 2σ orbitals forming the N≡N bond.

**Figure 23:**
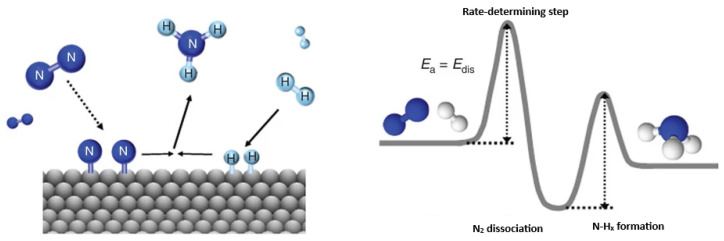
Schematic view of the reaction mechanism and energy profile for ammonia synthesis over conventional Fe/Ru-based catalyst, taken from the study of Kitano et al. [243]. The N_2_ and H_2_ molecules may react on the catalyst surface through a Langmuir-Hinshelwood mechanism to form NH_3_ in which N_2_ dissociation is the rate-determining step (RDS). The energy barrier (*E*_dis_) for this step corresponds to the apparent activation energy (*E*_a_) for ammonia synthesis.

The reduction of dinitrogen, N_2_, or nitrogen fixation, is a key reaction of the nitrogen cycle [233,234] and the importance of, for example, ammonia and nitrates in industry and agriculture has driven research into new methods of nitrogen fixation, often drawing inspiration from biological systems [235,236]. Thermodynamically, nitrogen reduction reactions (NRR) are feasible, indicated by negative Gibbs free energy. However, activating N_2_ under ambient conditions is challenging due to several factors: (1) a substantial energy gap (10.82 eV) between the HOMO and the LUMO, which hinders electron transfer; (2) a high enthalpy requirement (+37.6 kJ mol^−1^) for the initial protonation to form N_2_H^+^, preceding dinitrogen bond cleavage; (3) N_2_’s high stability and inertness, with a high bond dissociation energy (945 kJ mol^−1^), a significant first-bond breaking energy (410 kJ mol^−1^), the absence of a permanent dipole, and a large triplet state energy (6.17 eV); and (4) a negative electron affinity (−1.9 eV), low proton affinity (5.12 eV), and high ionization potential (15.85 eV) [237]. From the analysis of MESP [56], and as noted already above, we have shown that N_2_ possesses a belt of positive potential linked to its π* anti-bonding orbital. This region can accept electron density when N_2_ is adsorbed onto a catalyst surface such as an iron- or ruthenium-based catalyst [238,239], leading to the dissociative adsorption of N_2_ (Figure 23, left). Conventionally, the rate-determining step (RDS) in ammonia synthesis is the dissociation of N_2_ to two adsorbed nitrogen atoms (*2N, where * denotes the catalytic surface; cf. Figure 23, right). However, for many catalysts, the hydrogenation of adsorbed N atoms can become the RDS [240]. The activated N sites subsequently react with H atoms to form NH_x_ intermediates (x = 1–3), ultimately yielding NH_3_, which desorbs from the catalyst surface. This sequence of adsorption, activation, hydrogenation, and desorption is critical for efficient ammonia synthesis [238,241,242].

### 8.3. Battery Materials

The rational design of chemical systems for the development of battery materials is an active topic of research [244,245,246,247,248] that often employs the MESP model on molecular entities to examine their reactivity. Many studies have appeared during the last decade in which many electrolyte additives for the development of lithium-, and sodium-ion batteries have been proposed. For instance, bulky cations such as tetrabutylammonium (TBA^+^) and Cs^+^ have been proposed as electrolyte additives in Na-O_2_ batteries since these cations facilitate the stabilization of sodium superoxide in the electrolyte [249]. Similarly, Che et al. [250] used Rb^+^ and Cs^+^ as electrolyte additives for sodium-ion batteries that could significantly modify the chemical composition of solid electrolyte interphase (SEI) on hard carbon surfaces [245], resulting in a significant increase in the ionic conductivity and stability of the SEI. Wu et al. [13] have demonstrated that the electrostatic potential can be used as a solvent descriptor to probe the rational design of electrolytes for lithium-ion batteries. They emphasized that the lowest negative electrostatic potential ensures the nucleophilic capacity of the solvent whilst a relatively positive value means decreased solvation energy. The former and a high positive electrostatic potential are the main characteristics of non-solvating antisolvents.

In their discourse on lithium-ion batteries, Qin et al. [251] have presented the strategic employment of trifluoromethylbenzene (PhCF_3_), an aprotic co-solvent containing an electron-withdrawing trifluoromethyl group (–CF_3_) and an aromatic ring, to orchestrate the interfacial behavior of anions in the proximity of a graphite surface through subtle ion–dipole interactions. Unlike common additives such as fluoroethylene carbonate (FEC) and vinylene carbonate (VC) which are involved in Li^+^ solvation and sacrificially decompose on the graphite electrode to form an additive-derived solid electrolyte interphase (SEI), PhCF_3_ preferentially coats the graphite surface, catalyzing the decomposition of anions while preventing the co-intercalation of propylene carbonate (PC).

**Figure 24:**
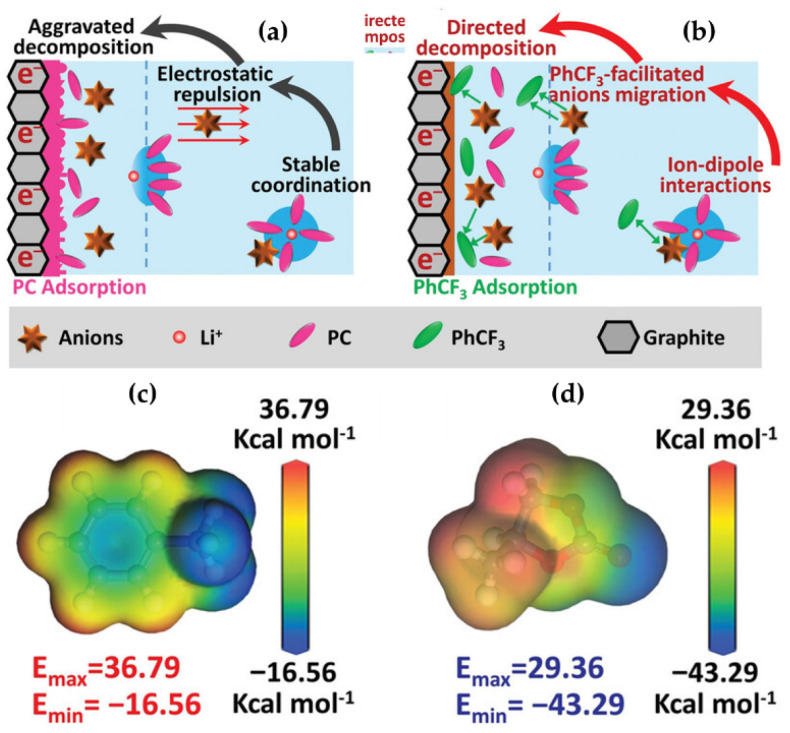
The interfacial behavior of Li^+^-PC complexes and anions near the surface of graphite electrode in (**a**) blank and (**b**) PhCF_3_-containing electrolyte. Shown are also MESP plots of (**c**) PhCF_3_ and (**d**) PC. (The isodensity envelope used to compute the potential was not mentioned in the study). Reproduced from the work of Qin et al. [251].

The mechanism of this process is depicted in Figure 24. In a barren electrolyte, anions, on nearing the graphite surface, detach from the Li^+^ solvation shell due to long-range electrostatic forces during the cathodic process (Figure 24a), leading to an accumulation of Li^+^-PC complexes near the graphite interface. The predominant decomposition of PC and the resultant formation of a fragile, fluffy SEI contribute to the eventual exfoliation of the graphite structure. However, when PhCF_3_ takes its place on the graphite surface through the π–π stacking, the ion–dipole interaction between anions and PhCF_3_ compensates for electrostatic repulsion, encouraging anions to gather on the graphite surface (Figure 24b). The robust SEI that emerges from this anionic decomposition fortifies the graphite anode within a PC electrolyte, even at modest concentrations of Li salts (<1 M). Thus, use of PhCF_3_ sidesteps the limitations of solvent-coordinated electrolytes (SCEs), such as high viscosity and prohibitive costs, while simultaneously securing a stable interface. The PhCF_3_-regulated SEI paves the way for the reliable performance of NCM613/graphite pouch cells, with over 300 cycles and an impressive 96% capacity retention. Furthermore, this electrolyte is versatile, with a wide liquid range (−70 to 160 °C) and high-voltage compatibility (4.4 V for NCM811/Li cells, 4.35 V for NCM613/graphite pouch cells).

The authors [251] used the MESP model to probe the nuanced ion–dipole interactions between PhCF_3_ and bis(fluorosulfonyl)imide anion (FSI^−^). Figure 24c and d represents the MESP plots of PhCF_3_ and PC, respectively. The potent electron-withdrawing character of fluorine in PhCF_3_ precipitates a significant electron accumulation on the trifluoromethyl group, manifesting as an *E*_max_ of 36.79 kcal mol^−1^ and an *E*_min_ of −16.56 kcal mol^−1^ (Figure 24c). (The study does not clarify what *E*_max_ and *E*_min_ refer to, but we assume they may represent the local most maxima and minima of potential on the surfaces of the molecules examined). This elevated *E*_max_ value of PhCF_3_ underscores a high thermodynamic propensity for interaction with anions. Moreover, the trifluoromethyl group in PhCF_3_, functioning as a Lewis acid center, exerts a strong attraction toward the negatively charged region of FSI^−^ anions. Consequently, these interactions were argued to enhance the dissolution of Li salts in PC, thereby intensifying the Li^+^-PC interaction, confirmed by various experimental observations such as NMR spectroscopy.

**Figure 25:**
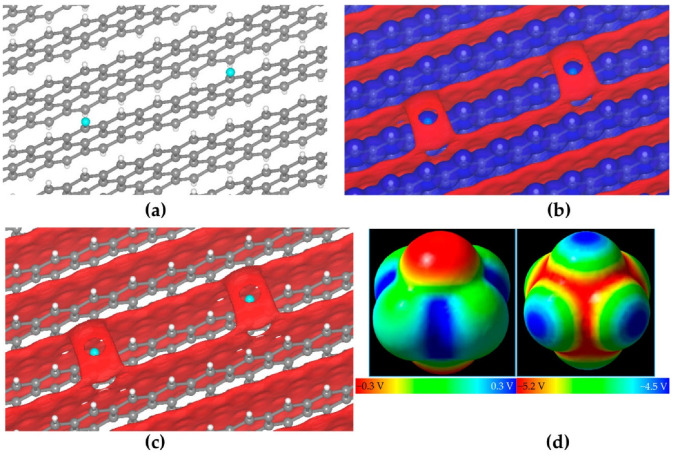
Passivated H-terminated (001) surface. (**a**) Crystal of passivated graphite with fluorine. Isopotential surface of graphite with a fluorine atom, separated between negative (red) and positive (blue) regions; (**b**) graphite atomic conformation and isopotential ±2.8 V; and (**c**) graphite atomic conformation and isopotential −2.8 V. (**d**) The MESP plot of (**left**) PF_6_ around its C_4_ axis and (**right**) the PF_6_^−^ anion. An electron density surface of 0.0004 a.u. was used on which to compute the potential. Reproduced from ref. [76].

Roman-Vicharra and coworkers [76] used theoretical methods to examine the existence of σ-holes on the surface of graphite anodes and of a few solid electrolytes (Li_3_PS_4_, Li_6_PS_5_Cl and Li_7_P_2_S_8_I) by exploring their electrostatic potentials. In order to understand why solid electrolytes such as Li_7_P_2_S_8_I (LPSI), are so promising for use in Li-ion batteries, they explored their ionic conductivity mechanism as a function of their electronic structure. To demonstrate the occurrence of σ-holes on molecular surfaces, they examined benzene and phenyl halogen molecules. The σ-holes were found on the halogens in the phenyl halogens, with a PhI > PhBr > PhCl trend in strength at a ±0.3 V isopotential. Using MESP, the study was then extended to the crystalline solid. LiPF_6_ is one of the most common salts for Li-ion batteries. Fluorine comes directly from the decomposition of PF_6_^−^ on the surface of the electrode, producing a σ-hole from −2.80 to −2.75 V; for other potentials there is no sigma-hole formation (Figure 25a–c). Chlorine comes from the decomposition of Li_6_PS_5_Cl, accompanied with a small σ-hole from −2.55 to −2.3 V. In case of the Li_7_P_2_S_8_I solid electrolyte, the I atom produced a σ-hole between −2.55 to −2.25 V. The strength of the σ-hole followed the order I > Cl > F. It was argued further that the σ-holes in the solid electrolytes form preferentially at the PS_4_ tetrahedron. Since Li-ions should be able to drift in any part of the battery, the fact that they can be attracted and eventually absorbed by regions of strong negative potential produced by high-electronegativity counterions becomes detrimental to ionic conductivity.

The authors [76] have suggested that PF_6_^–^ may be transiently oxidized. Neutral PF_6_ has a lower symmetry than its anion (*D_4h_* and *O_h_*, respectively). Their calculations show that PF_6_ has two negative potential caps along its shorter C_4_ axis (Figure 25d). By contrast, PF_6_^−^ has a positive potential on the outermost portion of each F along the P-F bond axis. However, given that PF_6_^−^ carries an overall charge of −1, the potential on the surface of the molecular entity should be negative everywhere. When color-coded, the surface of the molecular entity displaying blue, cyan, green, yellow, and red regions should all be equipped with negative electrostatic potential; such segmented colors arose because of asymmetrical distribution of the charge density on the surface of the molecular entity. This means that there should not the presence of any positive σ-hole on F in PF_6_^−^ since they are all purely negative, as like as the positive regions were observed everywhere on the surface of the CH_3_NH_3_^+^ and [NH_3_NH_3_]^2+^ cations [168], for example. Nevertheless, the authors of the study have shown that for neutral PF_6_, moving over a short range from ±0.30 to ±0.35 V gives us a fleeting instance of a σ-hole, which disappears quickly as they reach nuclear domain potentials close to the atoms.

### 8.4. Photovoltaics

A number of studies have been reported that used the MESP model to investigate donor–acceptor (D–A) structure features of molecular entities for application in organic [252,253,254,255,256,257,258], quantum/carbon dots [259,260], dye-sensitized [261,262,263] and organic-inorganic hybrid [264,265,266,267] solar cell semiconductors. Photophysical and optoelectronic properties such as the reorganization energy of electrons and holes, exciton binding energy, charge transfer, charge separation, bandgap, open circuit voltage (*V*_oc_), Fill factor (*ff*), short circuit current density (*J*_sc_) and efficiency (*η*), among others, have been explored. The MESP model was mainly invoked in these studies to reveal the presence of attraction between donor-acceptor moieties responsible for the functionality of the solar cells, as well as to shed light on the nature of “charge transfer” and “charge separation” between them [255,257,268]. Since charge generation is a crucial step to understand organic photovoltaics, Yao and coworkers [255] used the MESP model to develop a mechanism to explain efficient charge separation in non-fullerenes, a result which is contradictory to the accepted view that charge separation in organic solar cells emerges from sufficient energetic offset between the (polymer) donor and (fullerene) acceptor. Another interesting study that utilized the MESP model to understand how it assists in a basic understanding of charge transport in crystalline azaacene systems has been discussed elsewhere [269]. The authors of that study computationally analyzed the effect of the insertion of up to four nitrogen atoms (N-substitution) into the π-conjugated cores of anthracene, tetracene and pentacene. It has been found that N-substitution facilitates the π-stacking molecular arrangement in the corresponding crystals that generally results in large charge transfer integrals favorable for charge transport.

Yao et al. [255] investigated the wide bandgap polythiophene derivative PDCBT-2F as the polymer donor and the low bandgap non-fullerene small molecule IT-4F as the acceptor for organic solar cells, focusing on the influence of MESP calculations on charge generation. They found that MESP values are predominantly negative on the conjugated backbone for the donor and unevenly positive for the non-fullerene acceptor, allowing for the establishment of an internal electric field (IEF) at the donor-acceptor interface, directed from the donor to the acceptor. This IEF enhances exciton dissociation and facilitates electron transfer from the donor to the acceptor, which is crucial for charge generation in organic solar cells.

**Figure 26:**
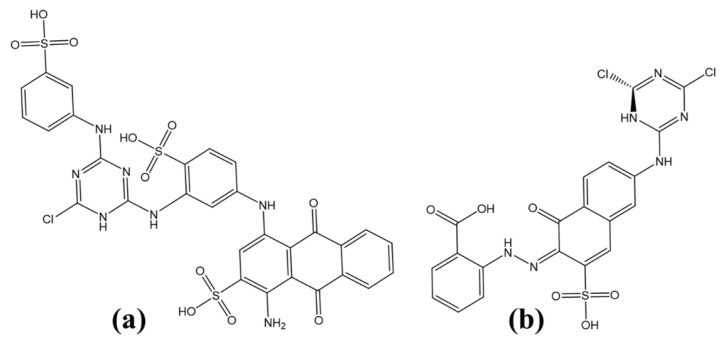
The structures of (**a**) RB 5 and (**b**) RB 10 dye. Reproduced from ref. [262].

Dindorkar et al. [262] used theoretical methods to modify reactive blue 5 (RB 5) and reactive brown 10 (RB 10) dyes, Figure 26, to improve their efficiency in designing dye sensitizers with enhanced efficiency for dye-sensitized solar cells (DSSCs). A benzodithiophene-based π-spacer was chosen (coded as BDTA) to design a dye sensitizer based on the donor-π-acceptor strategy. Analysis of the electrostatic potential maps of the BDTA spacer-based sensitizers showed a shift in charge density near the donor-π junction, indicative of the better transportability of electrons.

Ronca et al. [263] examined dye adsorption on the TiO_2_ conduction band energy in DSSCs, and demonstrated that an extensive charge rearrangement accompanies the dye–TiO_2_ interaction, which was revealed exploring the MESP of the donor-acceptor systems. This amounted to the transfer of 0.3–0.4 electrons from the dyes bound in a dissociative mode to the semiconductor.

Yanagisawa and coworkers [261] employed density functional theory-based molecular modeling (DFT/MM) to examine van der Waals (vdW) aggregation between small molecules, specifically KI and I_2_, in the presence of acetonitrile (AN) or 4-tert-butylpyridine (TBP). These interactions lead to the formation of KI_3_, and further vdW aggregation produces (KI_3_)_2_ with AN, (KI_3_)_2_ with (AN)_2_, and (KI_3_)_2_ with TBP in AN-based DSSC electrolytes. The calculated UV-Vis spectra of N_3_ (proton) dye with K^+^I_3_^−^ closely match the reported incident photocurrent efficiency (IPCE) action spectra (λ = 500–630 nm) of N_3_-sensitized DSSCs, verifying efficient electron transfer from the dye to TiO_2_ in the anode/TiO_2_/N_3_ (proton)/KI/I_2_/AN/cathode structure. The aggregation is driven by attraction between electron-poor (electrophilic) and electron-rich (nucleophilic) surfaces. The vdW aggregation of I_3_^−^ at DSSC interfaces facilitates unidirectional electron flow, contributing to high *ff* and *V_oc_*, and enhances electron diffusion in both K^+^I_3_^−^-based electrolytes and at the TiO_2_/N_3_ interface.

**Figure 27:**
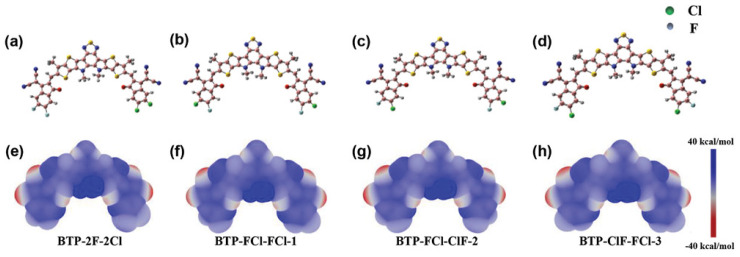
(**a**–**d**) Molecular structures and ESP distributions of BTP-2F-2Cl, BTP-FCl-FCl-1, BTP-FCl-FCl-2, and BTP-FCl-FCl-3, respectively. (**e**–**h**) Averaged ESP values of the atoms around the halogen atoms of the BTP-2F-2Cl compared with BTP-FCl-FCl-1, BTP-FCl-FCl-2, and BTP-FCl-FCl-3 respectively. Reproduced from Hu et al. [258].

The MESP plots for the atoms in an asymmetric Y_6_-like acceptor, BTP-FCl-FCl, and three of its isomers, reported by Hu et al. [258] are shown in Figure 27a–d. The halogen atoms (F and Cl) have negative potential values due to their high electronegativity, yet a large portion of the molecules show positive potential. The potentials of BTP-2F-2Cl and BTP-FCl-FCl are nearly identical, except for the eight carbon atoms adjacent to the halogens (Figure 27e–g). The average potential values for BTP-FCl-FCl slightly exceed those of BTP-2F-2Cl, while BTP-FCl-FCl also displays a smaller standard deviation (≈25 meV), indicative of a more uniform potential distribution. This heightened potential difference at the donor-acceptor (D/A) interface results in a stronger internal electric field (IEF), promoting exciton dissociation. Additionally, the potentials of the IC-2F and IC-2Cl end groups in BTP-2F-2Cl are 91.44 and 73.43 kcal mol^−1^, respectively, whereas that of the end groups in BTP-FCl-FCl exhibit nearly equal values. Consequently, BTP-FCl-FCl/BTR-Cl interfaces display a more significant and uniform IEF compared to BTP-2F-2Cl/BTR-Cl interfaces. Furthermore, the total dipole moments of BTP-FCl-FCl (≈0.4 D) are lower than those of BTP-2F-2Cl (0.73 D). Given the more complex contact at the D/A interface and the less uniform BTR-Cl blend film, the varied orientation of BTP-2F-2Cl molecules with higher dipoles may introduce greater electrostatic disorder, impeding charge mobility and device performance. The favorable nature of the potential and lower dipole of BTP-FCl-FCl enhance photo-induced electron transfer from donor to acceptor, leading to improved PCE, aligning with experimental findings.

### 8.5. Crystal Engineering and Anion Recognition

A number of studies reported in the last decade or so have used the MESP model to determine the acidity and basicity of specific regions of the electrostatic surfaces of a wide variety of molecular entities to rationalize the role these play in their solid state structure. While the majority have focused on crystals driven by directional halogen bonds (for example [31,82,131,185,204,270,271]), it is not uncommon to find studies in which, for example, tetrel bonds [58,89,189,272,273,274,275,276,277], chalcogen bonds [47,48,51,52,274] or pnictogen bonds [53,54,55,86,95] play an important role in the design of functional crystals. It has been shown that the order of solid state structures can be driven by halogen bonding between a covalently bonded halogen with an electrophilic site on it in one molecule, and a negative site on an interacting partner molecule (see for example [31,278,279]). Several studies [216,280,281,282,283,284] have focused on the application of comounds such as iodoperfluoroarenes, haloimidazolium, and halotriazole/triazolium as halogen bond donor motifs, and mechanically linked rotaxane and catenane skeletons as halogen bond anion host systems in anion receptor design, sensing, anion-templated self-assembly, and organocatalysis.

**Figure 28:**
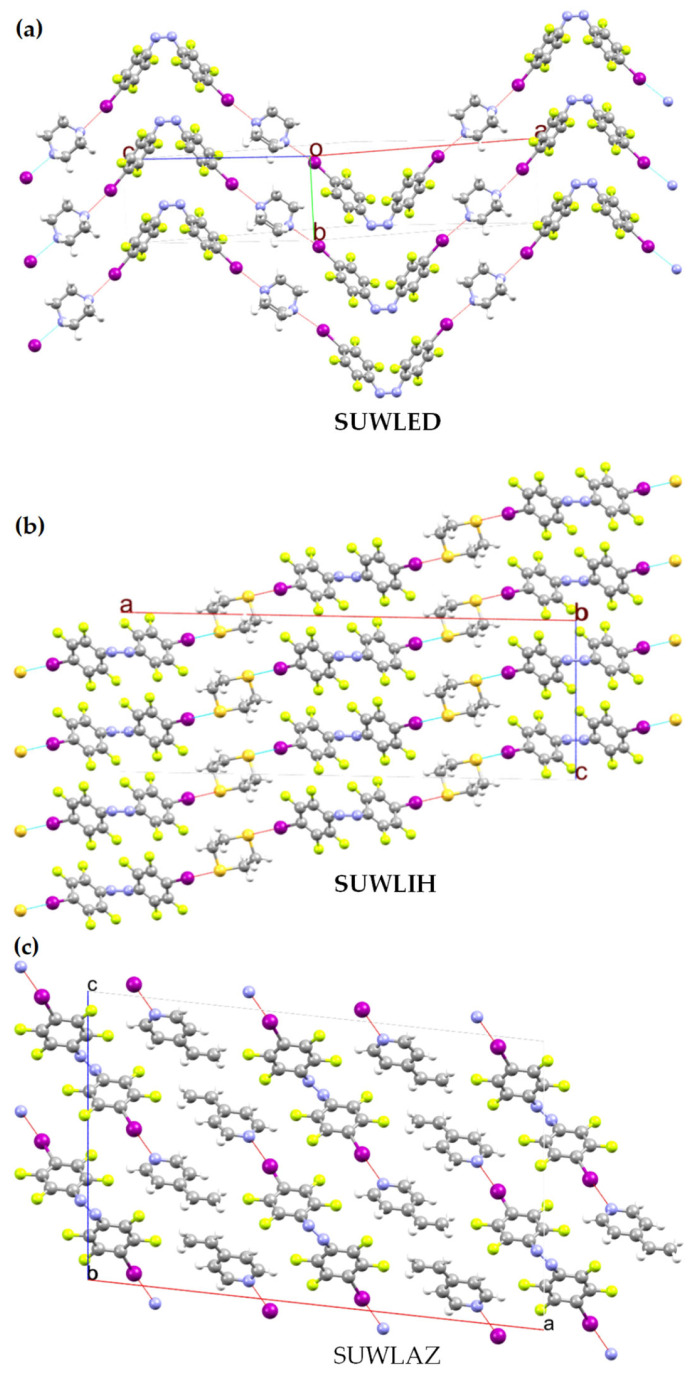
Examples of of I···N and I···S directional halogen bonding interactions (dotted lines in red) leading to ordered crystals of (**a**) bis(2,3,5,6-tetrafluoro-4-iodophenyl)diazene 1,4-diazabicyclo [2.2.2]octane; (**b**) bis(2,3,5,6-tetrafluoro-4-iodophenyl)diazene 1,4-dithiane; and (**c**) bis(2,3,5,6-tetrafluoro-4-iodophenyl)diazene bis(4-vinylpyridine). The CSD references are shown in uppercase letters. Atom types: H—gray white; N—blue; C—gray; F—green; S—yellow; I—purple.

Some examples of crystals engineered by directional Type-II halogen bonding are shown in Figure 28 [285]. The lone pair-dominant electron rich sites on N and S in 1,4-diazabicyclo [2.2.2]octane, 4-vinylpyridine, and 1,4-dithiane act as electron density donors. They each interact with the covalently bonded iodine atoms in bis(2,3,5,6-tetrafluoro-4-iodophenyl)diazene, resulting in the formation of directional halogen bonds. These interactions show up as I···N (Figure 28a,b) and I···S close contacts (Figure 28b). In particular, the co-crystallization of bis(2,3,5,6-tetrafluoro-4-iodophenyl)diazene with 1,4-diazabicyclo [2.2.2]octane resulted in needle-like crystals of (Figure 28a), consisting of antiparallel azo⋯phenyl stacks of bis(2,3,5,6-tetrafluoro-4-iodophenyl)diazene (with a repeat distance of 6.331(1) Å), laterally decorated with I···N halogen bonds to 1,4-diazabicyclo [2.2.2]octane molecules (I···N distance 2.758(2) Å) that bridge neighboring stacks. The co-crystallization of bis(2,3,5,6-tetrafluoro-4-iodophenyl)diazene and 1,4-dithiane gave long needle-like crystals (Figure 28b), consisting of antiparallel stacks of bis(2,3,5,6-tetrafluoro-4-iodophenyl)diazene (repeat distance 5.4079(4) Å) bridged by dithiane molecules connected through I⋯S halogen bonds (I⋯S distance 3.2907(5) Å). Similarly, needles of (bis(2,3,5,6-tetrafluoro-4-iodophenyl)diazene)(4-vinylpyridine)_2_, Figure 28c revealed that the antiparallel stacks of bis(2,3,5,6-tetrafluoro-4-iodophenyl)diazene (stack repeat 5.4157(7) Å) are laterally decorated with halogen-bonded 4-vinylpyridine electron density acceptors (I⋯N distance 2.780(2) Å).

**Figure 29:**
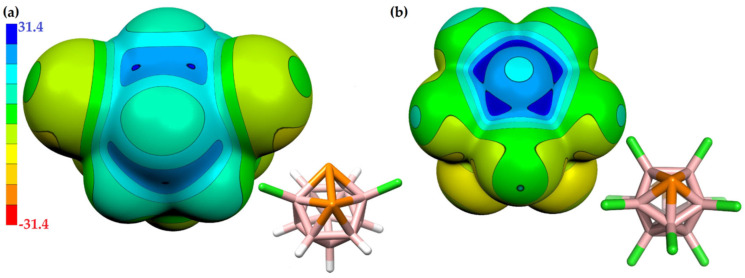
The [HF/cc-pVDZ] computed molecular electrostatic potential surface and molecular diagram of (**a**) 3,6-Cl_2_-*closo*-1,2-P_2_B_10_H_8_ and (**b**) *closo*-1,7-P_2_B_10_Cl_10_. The color range in the bar is in kcal mol^−1^. Reproduced from ref. [288].

Single crystals such as 3,6-Cl_2_-*closo*-1,2-P_2_B_10_H_8_ [286] and the 12-vertex *closo*-phosphaborane *closo*-1,7-P_2_B_10_Cl_10_ with toluene [287] are types of heteroboranes in which hypervalent phosphorous atoms play a significant role in crystal packing. Although these crystal structures were reported some 20 years ago, a recent computational analysis [288] of their structures was performed to shed light on the nature of the noncovalent interactions responsible for the crystal packing. The MESP plots shown in Figure 29a suggest that a σ-hole on each Cl atom along the extension of the B–Cl bond in Cl_2_-*closo*-1,2-P_2_B_10_H_8_ is highly localized and positive (green region) but the lateral portion of the same atom is weakly nucleophilic. The electrophilicity of Cl in *closo*-1,7-P_2_B_10_Cl_10_, Figure 29b, increases as the number of Cl atoms in the molecular entity increases. By contrast. The electrophilic strength of hypervalent P atom is stronger in *closo*-1,7-P_2_B_10_Cl_10_ than in Cl_2_-*closo*-1,2-P_2_B_10_H_8_, which can be appreciated from the colors that represent the electrostatic potential.

**Figure 30:**
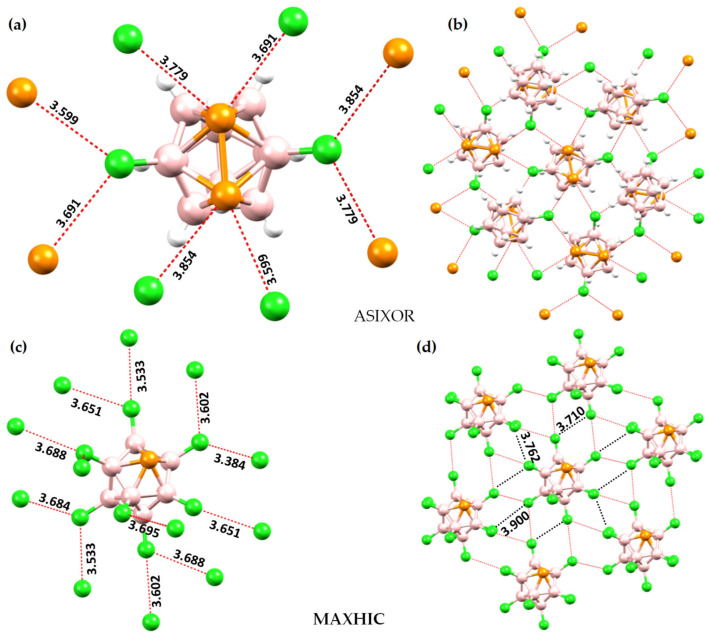
The dominant intermolecular interactions in the crystal of (**a**) 3,6-dichloro-*closo-*1,2-diphosphadodecaborane(12)(H_8_B_10_Cl_2_P_2_); (**b**) 2,3,4,5,6,8,9,10,11,12-decachloro-*closo*-1,7-diphosphadodecaborane toluene solvate. Selected bond distances are in Å. For clarity, toluene is deleted from (**b**). The CSD reference code is shown in uppercase letters in each case.

Our geometric analysis suggests that each P site in 3,6-Cl_2_-*closo*-1,2-P_2_B_10_H_8_ is involved in an attractive engagement with two Cl atoms of two neighboring molecules, forming directional P⋯Cl pnictogen bonds (Figure 30a) that are either shorter or longer than the sum of the van der Waals radii of Cl and P, 3.72 Å [289]. The network of P···Cl pnictogen bonds formed by the central 3,6-Cl_2_-*closo*-1,2-P_2_B_10_H_8_ with six nearest neighbors is shown in Figure 30b. There are also a number of H···Cl hydrogen bonds between the molecular entities that assist in the crystal packing.

The packing in the crystal of *closo*-1,7-P_2_B_10_Cl_10_ is driven by several intermolecular interactions: H···Cl hydrogen bonds, P···π(arene) pnictogen bonds, Type-II Cl···Cl halogen bonds, and Cl···Cl Type-I halogen bonds. Shown in Figure 30c,d are halogen bonds with intermolecular bond distances between 3.384 and 3.900 Å. The Type-I halogen bonds were always longer than twice the sum of the van der Waals radius of Cl, 3.64 Å; two of them are marked in Figure 30d, with Cl···Cl distances of 3.762 and 3.710 Å. However, Fanfrlík and Hnyk [288] are of the view that the crystal is dominated by B–Cl···Cl–B Type-I halogen bonds (they referred to them as dihalogen bonds!) and strong B-P···π pnictogen bonds (PnBs). The latter bond has an intermolecular bond distances of 3.08 Å and an [MP2.5/CBS] interaction energy of −10.55 kcal mol^−1^.

**Figure 31:**
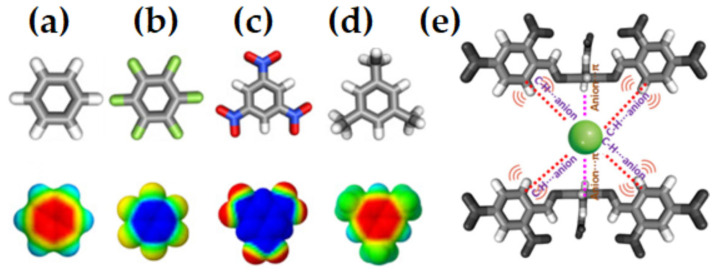
MESP surfaces of (**a**) benzene, (**b**) hexafluorobenzene, (**c**) trinitro benzene and (**d**) trimethylbenzene. Shown in (**e**) are the intermolecular interactions (C-H···anion and π···anion) between the receptor and the anion in the receptor-anion complex. (No information about the nature of the isodensity density envelope and computational methods used were provided.) Reproduced from ref. [290].

Lakshminarayanan et al. [290] used the MESP model as a web-based tool (MolView [291] th) to support their experimental deductions concerning C-H···anion and anion···π interactions in several receptor-anion complexes. The receptors included benzene, hexafluorobenzene, trinitrobenzene, and trimethylbenzene. As expected from the various studies described above, the centroid of the fully fluorinated and NO_2_-substituted arene derivatives act as an electrophile (positive π-holes, blue regions, Figure 31) because of the presence of electron-withdrawing groups, while that of benzene and methyl substituted benzene act as a nucleophile (negative π-hole, red regions) because of peripheral H atoms and methyl groups that are electron donating groups. However, as the authors noted, MolView should be used with caution since it might not yield accurate data, but the information gathered using such codes might be useful for receptor design and to explain the selectivity and nature of interaction present in the host-guest complex. Based on the results noted above, the authors have designed a receptor host L consisting of nitro substitutions on the aryl moieties capable of binding a variety of anions (fluoride, chloride, cyanide, acetate, bifluoride, etc.). The binding between them, Figure 31e, was governed by several non-bonding interactions, such as C-H···anion and π···anion interactions.

The importance of halogen [281,282,292,293], tetrel [58,275,276,281], chalcogen [52,294,295,296], and pnictogen bonding [281,297,298,299] in anion recognition is well recognized. While do not cover such studies here, we just emphasize that anion binding is a crucial field of research for the fundamental understanding and development of, for example, novel receptors for sensing and transport.

Scheiner [276] evaluated the ability of tetrel atoms Tt (T = Si, Ge, Sn, Pb) in molecular receptors to bind various anions with strong tetrel-bond interactions as a guide for designing optimal anion receptors, a study that focuses on interaction energetics and receptor design rather than on molecular electrostatic potential maps. The Sn atom appeared to form the strongest tetrel bond of the tetrel family. Connecting an –SnF_3_ group to an imidazolium or triazolium yielded a strong halide receptor. Placing a pair of –SnF_3_ groups on one molecule to form a bimodal dicationic receptor with two tetrel bonds increased the strength of the tetrel bond, but did not lead to a simple doubling of the bond strength. The two tetrel groups were placed on opposite sides of an alkyl diamine chain of any length, but SnF_3_^+^NH_2_(CH_2_)*_n_*NH_2_SnF_3_^+^ with *n* between 2 and 4 showed the strongest halide binding. Of the various anions tested, OH^−^ binds most strongly, with the order observed: OH^−^ > F^−^ > Cl^−^ > Br^−^ > I^−^. While the binding energies of the larger NO_3_^−^ and HCO_3_^−^ anions were shown to be highly dependent on the charge of the acceptor, a bipodal dicationic receptor has advantages over a monocationic one which can engage in only a single tetrel bond.

**Figure 32:**
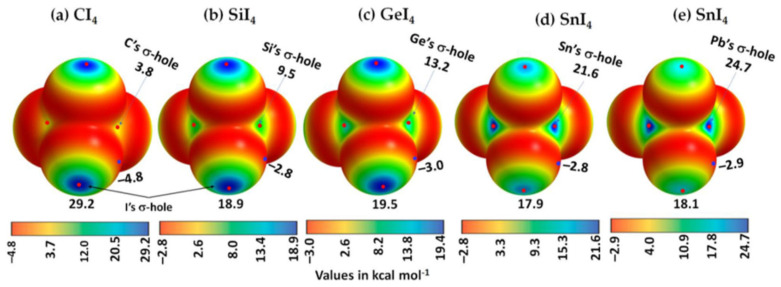
[*ω*B97X-D/def2-QZVPPD] level 0.001 a.u. isodensity envelope mapped potential on the electrostatic surfaces of (**a**) CI_4_, (**b**) SiI_4_, (**c**) GeI_4_, (**d**) SnI_4_, and (**e**) PbI_4_. The strength of Tt’s and I’s σ-holes is shown in each case; filled tiny blue and red circles represent *V*_S,min_ and *V*_S,max_, respectively, and are in kcal mol^−1^. Reproduced from ref. [58].

One of us [58] has used the MESP model to demonstrate the ability of I_4_Tt to recognize halide anions leading to the formation of 25 molecule–anion complex systems [I_4_Tt···X^−^] (Tt = C, Si, Ge, Sn and Pb; X = F, Cl, Br, I and At). It was observed that the strength of electrophilic character on the surface of the tetrel atom increases in the series from C to Pb in I_4_Tt (Figure 32) which had a profound effect in controlling its anion selectivity. The tetrel bond strength in the [I_4_C···X^−^] series and [I_4_Tt···X^−^] (Tt = Si, Sn; X = I, At) was weak-to-moderate, whereas that in the remaining 16 complexes the bond has a distinct dative tetrel bond character with significant interaction energies (from −3.0 to −112.2 kcal mol^−1^ at the [CCSD(T)/def2-TZVPPD] level of theory) and short Tt···X close contact distances.

**Figure 33:**
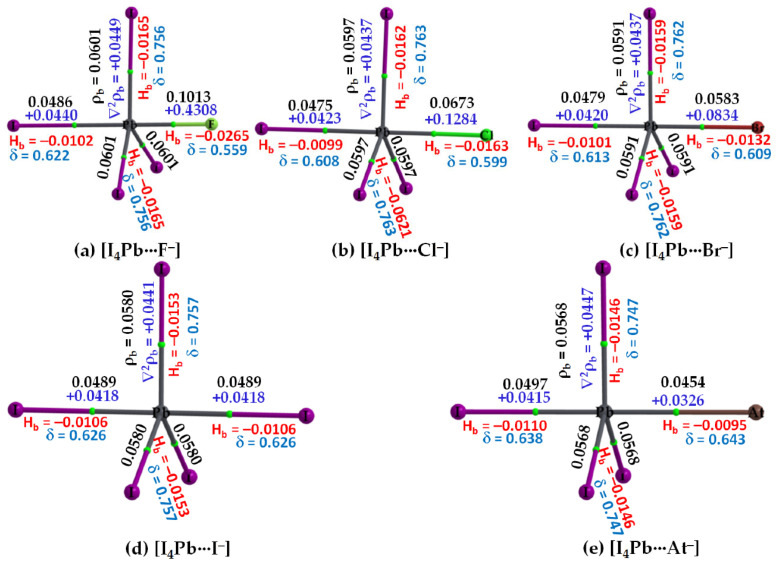
[*ω*B97X-D/def2-QZVPPD] level QTIM-based molecular graphs of [I_4_Pb···X^−^] (X = F, Cl, Br, I, At)—see Figure 32—showing the bond paths (solid and dotted lines in atom color) and bond critical points (tiny spheres in green) between bonded atomic basins. Large spheres represent the atomic basins, with atoms labeled. The charge density (*ρ*_b_), the Laplacian of the charge density (∇^2^*ρ*_b_), the total energy density (*H*_b_), and the delocalization index (*δ*) values (a.u.) are shown in black, blue, red, and faint-blue fonts, respectively. Reproduced from ref. [58].

The significant variation in interaction energies was shown to be the result of different levels of tetrel bonding, comprising of ordinary and dative tetrel bonds, between the interacting partners at the equilibrium geometries of the complex systems. The formation of dative tetrel bonds between I_4_Pb and X^−^ in [I_4_Pb···X^−^] is shown in Figure 32; this is supported by QTAIM-based charge density topological properties (charge density, *ρ*_b_; the Laplacian of the charge density, ∇^2^*ρ*_b_; and the total energy density, *H*_b_), shown in Figure 33.

**Figure 34:**
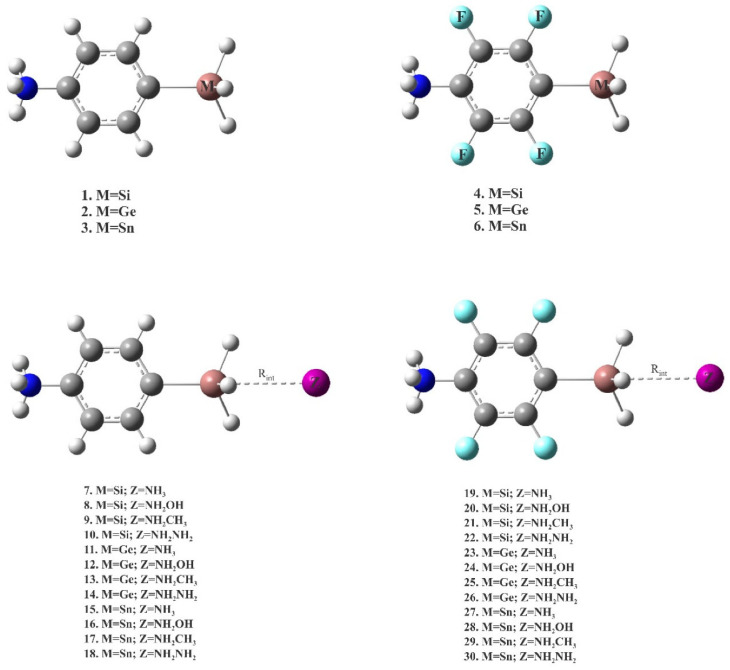
Illustration of the geometries of monomers **1**–**6** and cationic tetrel-bonded complexes **7**–**30**. Atoms shown as balls: H—white; C—gray; N—blue; F—cyan; M = faint-orange; Z = purple. Reproduced from ref. [275].

The nature of the intermolecular bonding observed in ref. [58] is in agreement with the conclusions reached by Esrafili and Mousavian [275]. They examined charge-assisted tetrel-bond interactions between neutral molecules XF_3_M (X=F, CN; M=Si, Ge and Sn) and several anions A^−^ (A^−^ = F^−^, Cl^−^, Br^−^, CN^−^, NC^−^ and N_3_^−^), as well as that between the cations [p-NH_3_(C_6_X_4_)MH_3_]^+^ (M=Si, Ge, Sn; X = H, F) and neutral molecules Z (Z = NH_3_, NH_2_CH_3_, NH_2_OH and N_2_H_4_) (Figure 34). The application of the MESP model to the XF_3_M series revealed that the *V_S,max_* of bound Sn atom in F_4_Sn (*V_S,max_* = 96.5 kcal mol^−1^) is greater than that of Ge (*V_S,max_* = 70.2 kcal mol^−1^) and Si (*V_S,max_* = 57.3 kcal mol^−1^) in GeF_4_ and SiF_4_, respectively. The σ-hole on the surface of M in MF_3_CN was more positive as the size of the M atom increased from Si to Ge to Sn. The [MP2/aug-cc-pVTZ] level M···A^−^ intermolecular bonding distances for a given anion type increased in the order SiF_3_CN < SiF_4_ < GeF_3_CN < GeF_4_ < SnF_3_CN < SnF_4_. The interaction energies for the anionic complexes varied between −16.35 and −96.30 kcal mol^−1^ (F_4_−Si···Br^−^ and CNF_3_−Sn···Br^−^, respectively), suggesting that the tetrel bonding interactions leading to the formation of many of these anionic complexes have dative bond character. By contrast, the [MP2/aug-cc-pVTZ//MP2/aug-cc-pVDZ] level interaction energy for cationic complexes varied between −7.69 and −26.98 kcal mol^−1^ (1+NH_3_ and 6+NH_2_NH_2_, respectively; see Figure 34 for configurations 1 and 6), which feature moderate to strong tetrel bonds. There was no obvious relationship between the *V_S,min_* values associated with a given electron density donor (such as in NH_2_NH_2_) and the interaction energies. This, it was speculated, was a consequence of the involvement of secondary interactions between these Lewis bases and the H atoms of -MH_3_ in the Lewis acid.

**Figure 35:**
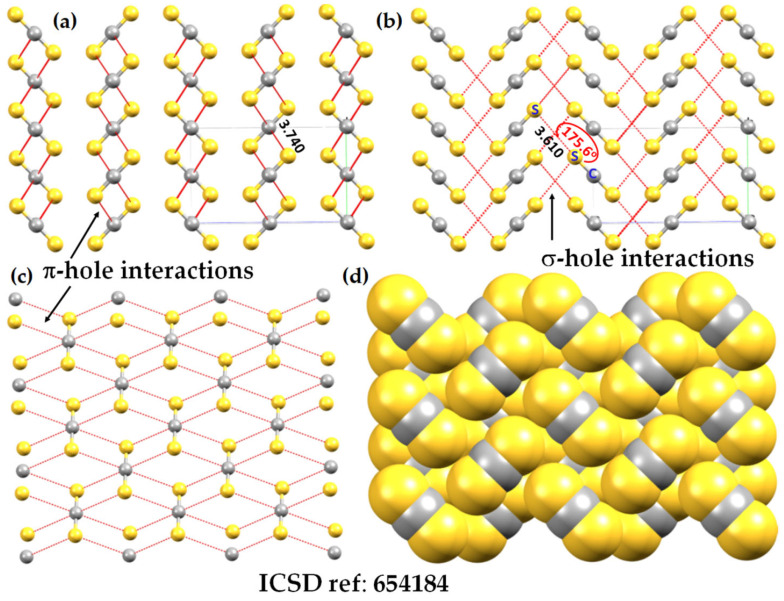
The nature of the intermolecular interactions in the crystal structure of CS_2_: (**a**) directional π-hole centered tetrel bonded interactions; (**b**) directional σ-hole centered chalcogen bonded interactions; (**c**) the possible number of π-hole centered interactions formed by the tetrel donor C site in each CS_2_; (**d**) the space-filling model of the nature of packing between the CS_2_ molecules in the crystal. Shown in (**a**,**b**) are selected bond distances (Å) and angles (degrees).

A delightfully simple yet profoundly illustrative example of how the collective involvement of a σ-hole and a π-belt plays a role in its structure determination is the crystal structure of carbon disulfide (CS_2_). The types of interaction between CS_2_ molecules can be effectively deduced from the MESP model of the CS_2_ monomer [115,300]. As shown in Figure 35a, each layer of CS_2_ in the crystal is governed by (C)_π_···S type π-belt interactions. Each layer is connected to the nearest layer by C=S(σ)···S type σ-hole interactions, Figure 35b, which are highly directional (∠C=S(σ)···S = 175.6°). The π-hole interactions are tetrel bonds centered on the π-holes, while the σ-hole interactions form the σ-hole centered chalcogen bonds. Within this structure, each sulfur atom (the donor of electron density) participates in forming two (C)_π_···S type π-belt interactions. Simultaneously, each carbon atom (as the acceptor of electron density) is engaged in the formation of four π-hole centered interactions, as shown as in Figure 35c; the space-filling model is shown in Figure 35d. These types of interactions epitomize the delicate, yet powerful, forces that shape the very essence of molecular and crystal structures.

The burning of fossil fuels emits a significant amount of CO_2_, causing climate change concerns. Carbon capture and storage (CCS) aims to reduce emissions into the atmosphere, with fullerenes showing promise as adsorbents. In light of this, Rezaee et al. [301] carried out a DFT study on some B, N, and P doped C_20_ (C_20-*n*_X*_n_*, *n* = 0, 1, 2, and 3; X = B, N, and P) to evaluate the adsorption energy, height, and the CO_2_ angle. The study demonstrated that the B and N-doped fullerenes had interactions with CO_2_ the strength of which far exceeded the interaction with clean C_20_, changing a physisorption to a physicochemical adsorption.

**Figure 36:**
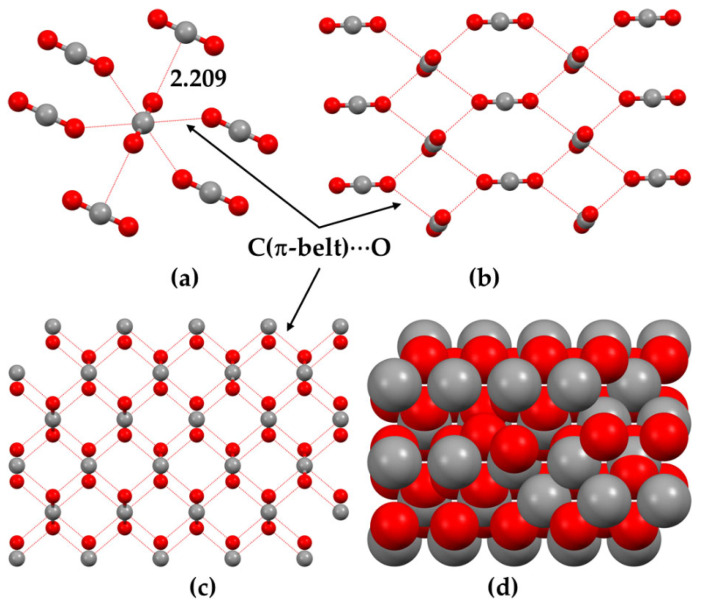
Three different views (**a**–**c**) of the nearest neighbor interactions of CO_2_ in its tetragonal crystal structure [303], and (**d**) the space-filling model. The experimentally determined crystal is reported elsewhere [305]. The C(π-belt)⋯O bond distance (Å) is marked in (**a**).

The (CO_2_)_2_ dimer, at the [CCSD(T)-F12/aug-cc-pVTZ] level of theory, has a “slipped parallel” configuration, whilst the T-shaped structure is merely a transition state [302]. This conclusion was reached after a thorough exploration of the configurational space of the dimer. It accords with the solid state structure of CO_2_. There are several structures known. For instance, the cuprite structure (tetragonal space group *P4_2_/mnm* [303]) is zero-dimensional and consists of two CO_2_ clusters. The cyanogen chloride-derived structure crystallizes in the orthorhombic *Pbcn* space group [304]. The structure is zero-dimensional and consists of four CO_2_ clusters. C^4+^ is bonded in a linear geometry to two equivalent O^2−^ atoms. Both C=O bond lengths are 1.17 Å. In the tetragonal crystal, each C-site and O-site is bonded noncovalently to at least four O and two C atoms of neighboring molecules. As illustrated in Figure 36a,b, the CO_2_ molecules engage in π-hole interactions with their nearest neighbors, weaving a complex web of molecular connections within a layer. They are all of the C(π-belt)⋯O_2_C type; thus the electron deficiency around the C atom in CO_2_ is able to accept electron density from four O sites of four nearest neighboring CO_2_ molecules. Shown in Figure 36c, is a view of the extended crystal structure, further illustrated in Figure 36d with a space filling model.

These observations accord with the 0.002–0.004 a.u. isodensity envelope surfaces mapped potential of CO_2_ [306,307,308,309]. The C=O bond is strongly polarized towards oxygen due to its higher electronegativity. CO_2_ has a polarizability of 26.3 × 10^−25^ cm^3^ which is only ∼50% higher compared to N_2_ (17.6 × 10^−25^ cm^3^). The quadrupole moment of the CO_2_ molecule (13.4 × 10^−40^ C m^2^) is a little higher than that of N_2_ (4.7 × 10^−40^ C m^2^) [310]. The bonding region around the carbon atom is equipped with a wide belt of positive potential, and the O and C-ends with a negative potential. Accordingly, and as indicated above, it was shown that CO_2_ can form tetrel bonds perpendicular to its C_∞_ axis, via the electrophilic belt around the C atom, with, for example, the lone-pair of a Lewis base, thus forming π-hole centered B⋯CO_2_ complexes, where B = CO, HCCH, HCN, NH_3_, H_2_O, H_2_S, and PH_3_ [115]. This is different to CS_2_, which forms chalcogen bonds with the same bases via the electrophilic region that lies at each S atom and is centered on the C_∞_ axis (σ-hole interaction). N_2_O also has a small electric dipole moment, and therefore forms a complex with a given Lewis base of similar geometry to that of its CO_2_ counterpart, but with small distortions resulting from the lower symmetry and the non-zero electric dipole moment in the case of N_2_O [115].

**Figure 37:**
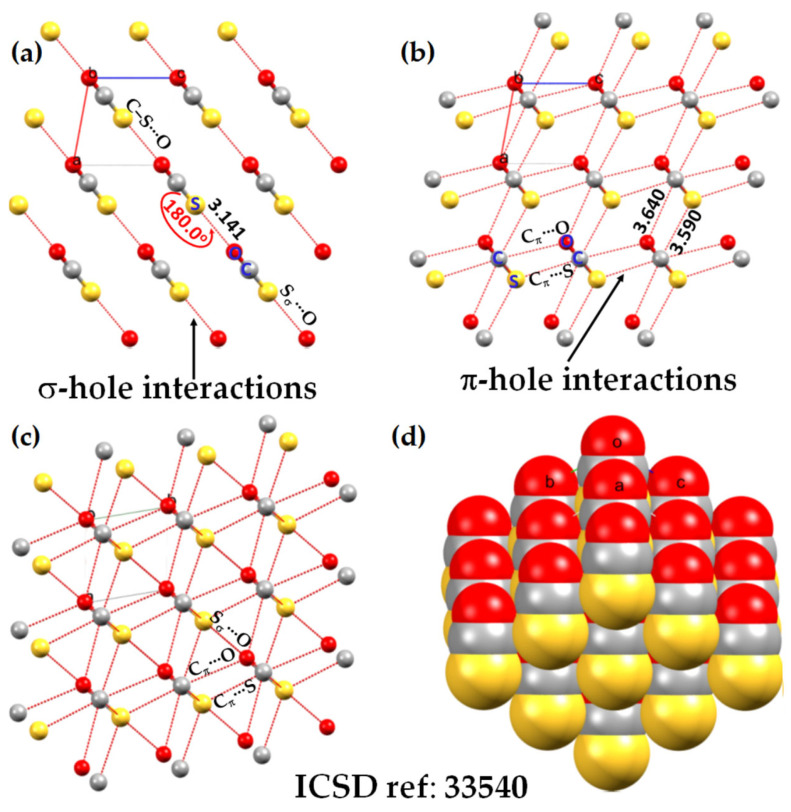
Illustration of the nature of intermolecular interactions (dotted lines in red) in the crystal of OCS: (**a**) directional σ-hole interactions; (**b**) directional p/π-hole interactions; (**c**) combined σ- and p/π-hole interactions; and (**d**) the space-filling model depicting the packing arrangement of OCS molecules in the crystal. The ICSD code is provided in (**d**), and selected intermolecular bond distances (Å) and angles are displayed in (**a**,**b**).

The refined structure of the crystalline carbonyl sulfide (OCS) has been known since 1982, stabilized at 90 K, and examined using neutron powder diffraction [311]. This structure has rhombohedral symmetry (*R3m*), with a single molecule in the unit cell aligned along the [111] crystallographic axis. The bonding between OCS molecules is shown in Figure 37. Two types of intermolecular interactions can be observed. In Figure 37a is shown the S⋯O σ-hole interactions, while in Figure 37b is illustrated C⋯O and S⋯O p/π-belt interactions. A configuration where both interaction types coexist is shown in Figure 37c. Meanwhile, Figure 37d reveals The crystal structure depicted using a space filling mofrl is given in Figure 37d.

A number of experimental and theoretical studies have been conducted using the MESP model to explore the interactions of OCS molecule with a variety of entities, including, for example, chloride (Cl^–^); (OCS)*_n_* clusters, *n* = 2–8 [312]; trimethylamine (CH_3_)_3_N [313,314]; para-substituted pyridine [315,316]; and 1,2-dihydroxybenzene and 1,2-dimethoxybenzene [316]. It was shown that the sulfur atom in OCS has a σ-hole along the extension of the C=S bond, with a *V_S,max_* value of 87.4 kJ mol^−1^ at the [MP2=full/aug-cc-PVDZ] level [315]. A belt positive electrostatic potential is seen around the carbon atom, with *V_S,max_* = 86.1 kJ mol^−1^. The *V_S,max_* values for the S and C in OCS are significantly higher than those in CS_2_, where for S, *V_S,max_* = 70.9 kJ mol^−1^ and for C, *V_S,max_* = 23.1 kJ mol^−1^ [317]. This difference is clearly due to the presence of O in OCS. Interestingly, the sulfur atom in OCS also develops a belt of negative electrostatic potential around it with *V_S,min_* = −6.3 kJ mol^−1^, although the most negative portion of the molecule is centered on the O atom along the outermost extension of the C=O covalent bond (*V_S,min_* = −60.9 kJ mol^−1^). It was demonstrated that the fully relaxed geometries of the binary complexes of OCS with substituted pyridine comprising of either chalcogen or tetrel bonds, similar to those reported for the pyridines complexes of CS_2_ [315]. The absolute values of potential of course change when changing the isodensity envelope used of their calculation, but the overall picture remains the same. Singh and Gadre [318] used ab initio methods to explore the clusters of CO_2_, CS_2_ and OCS, and compared the intermolecular interaction topologies with those observed in their crystal structures.

### 8.6. Electrostatic Potential Maps of Substitution and Elimination Reactions

An understanding of reaction mechanisms in many branches of chemistry stands as a formidable challenge to the novice, often driving students to retreat into rote memorization to navigate the labyrinth [89,319,320]. Despite efforts to champion mechanistic reasoning as a more profound approach, the elusive grasp of the underlying chemical principles continues to confound many. In this context, the study by Nelson and colleagues [24] provides much insight. They propose that integrating electrostatic potential maps into the organic chemistry curriculum may serve as a powerful tool for deepening students’ comprehension. By promoting deeper sense-making among students, this approach has the potential to elevate their grasp of reaction mechanisms from simple memorization to genuine comprehension.

The view aligns with the findings of Shin et al. [321], who demonstrated that electrostatic potential can serve as a physically meaningful metric for predicting substituent effects. The authors conducted extensive DFT calculations on over 400 molecules, followed by rigorous statistical analyses. Their study revealed a linear correlation between electrostatic potential and substituent parameters across various reaction systems. They showed that MESP could be widely adopted to enhance the validity of linear free energy relationships (LFERs) under different chemical conditions. The results indicated that the MESP shift induced by a functional group on a monosubstituted benzene ring is a strong predictor of substituent effects on the compound’s electronic behavior in chemical reactions. As such, MESP can serve as a firmly grounded alternative to traditional empirical parameters as such the Hammett [322] or Swain–Lupton parameters [322,323], or the charge shift [324].

Utilizing a topography-based electrostatic docking model, Gejji et al. [325] explored ion pair formation involving the trifluoromethanesulfonate ion (T_f_¯) with cations such as Li^+^, Na^+^, and NH_4_^+^. Their study demonstrated that MESP could effectively predict various minima, transition states, and saddle point structures of these ion pairs on the potential energy surface. Similarly, Mottishaw et al. [326] utilized electrostatic potential maps to visualize and conceptualize Sanger’s pivotal observation regarding the different reactivities of 2,4-dinitrochlorobenzene and 2,4-dinitrofluorobenzene. This approach was further extended to compare the reactivity of a series of halobenzenes in nucleophilic aromatic substitution (S_N_Ar) fluorination, a reaction of significant importance in pharmaceutical and medicinal chemistry. When combined with experimental data from the literature, the electrostatic potential maps align with and strongly corroborate the observed reactivities of various substrates in S_N_Ar reactions. Similarly, Wilmot et al. [326] focused on developing a straightforward technique to precisely predict and visualize the diastereoselectivity of reductions involving ketones, aldehydes, and allyl chlorides. By mapping electrostatic potential onto the frontier molecular orbital involved in the reduction, the approach highlights a difference in electrostatic potential on the faces of the carbonyl group, allowing for highly accurate predictions of the nucleophilic attack’s preferred face.

Of relevance to molecular entities in interstellar space, Bhasi and coworkers [327] used theoretical methods to scan the potential energy surface of the reaction between NH and NS which produces two very stable species, HNSN and HNNS. HNNS has a N–N linkage and is the more stable. The reaction can lead to the formation of N_2_ via the HNNS isomer and tunneling effects may make this reaction feasible even at the extremely low temperatures of the interstellar medium. Supported by a MESP analysis, a similar mechanism was suggested for the reaction between NH and NO, positioning HNNS and HNNO as potential stable reservoirs for interstellar N_2_.

**Figure 38:**
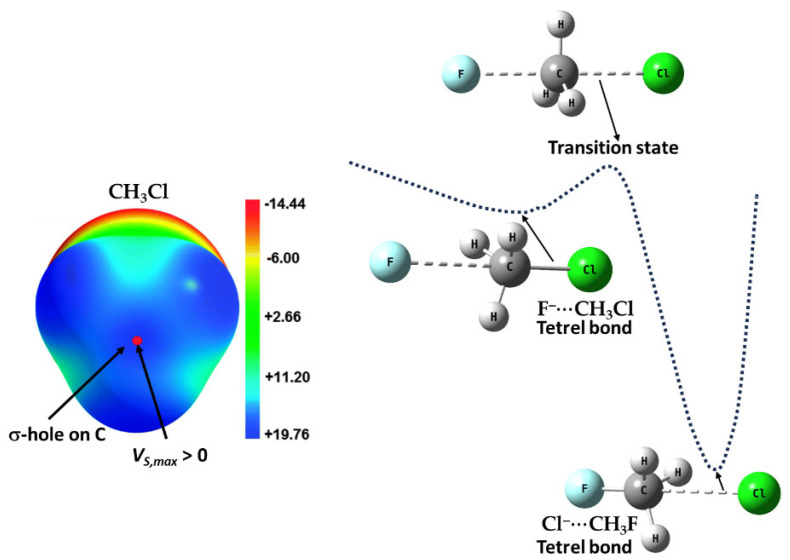
(**Left**) The MESP plot of CH_3_Cl, showing the σ-hole on the carbon atom (values in kcal mol^−1^) charactered by the maximum of potential (*V_S,max_*). (**Right**, schematic) The reaction profile of the S_N_2 reaction mechanism of F^–^ + CH_3_Cl ⟶ Cl^–^ + CH_3_F.

In an insightful article, “Fifty Years of Nucleophilic Substitution in the Gas Phase,” Wester [319] explored S_N_2 reactions at a saturated carbon atom, X^–^ + CR_3_Y ⟶ Y^–^ + CR_3_X, where X and Y are halogens, and R = H or another substituent. While the article does not discuss the application diversity of the MESP model, it is pertinent to consider Grabowski’s earlier work. Grabowski [89] used MP2/aug-cc-pVTZ calculations to study complexes of ZH_4_, ZFH_3_ and ZF_4_ (Z = C, Si and Ge) with HCN, LiCN, and Cl^−^, which act as Lewis bases through their nitrogen or chlorine centers. The Z-atoms in these complexes were found to serve as Lewis acid centers, forming σ-hole bonds with the Lewis bases (with exceptions for some complexes of CH_4_, CF_4_, and F_4_Si). The S_N_2 mechanism detected for the Sn and Ge analogs are not observed for the Si moieties since the positive electrostatic potential at the central reaction site on the tetrel atom decreases in the order Sn > Ge > Si > C. The analysis of the electrostatic potential surfaces of these interacting species revealed that the electron charge redistribution associated with tetrel bond formation is similar to that observed in S_N_2 reactions. This is readily understood for the reaction F^–^ + CH_3_Cl ⟶ Cl^–^ + CH_3_F, which has a small activation energy barrier between them (cf. Figure 38) [89]. Of course, the carbon’s σ-hole on CH_3_Cl, plays the vital role as an electrophile to drive the reaction (see Figure 38, left). Other similar studies have been reported [328,329,330,331].

### 8.7. Medicinal Chemistry, Drug Design and Biologically Relevant Systems

Since the influential work of Auffinger et al. in 2004 [332], the MESP model has been extensively used to enhance our understanding of the physical chemistry and chemical physics of many protein-ligand and protein-protein biologically relevant systems [5,40,333,334]. The model has been particularly valuable in elucidating the role played by halogen bonding involving ligands important in medicinal chemistry and in drug design, and in ligands such as thyroid hormones and inhibitors, which bind specifically to proteins and nucleic acids, useful in the rational design of more effective inhibitors for therapeutic targets and in the development of biologically-active materials [185,335,336]. As Politzer and Murray demonstrated [337], a very useful application of the MESP model in biochemistry and pharmacology to identify characteristic patterns of positive and negative potentials that either promote or inhibit particular types of biological activities. To this end, Frontera and coworkers [338] have highlighted the importance of σ-hole centered halogen bonds in protein ligand complexes, including the characterization of the Protein Data Bank (PDB) structures driven by (C–I⋯A, A = O, S, Se, π) halogen bonded (HaB) interactions.

**Figure 39:**
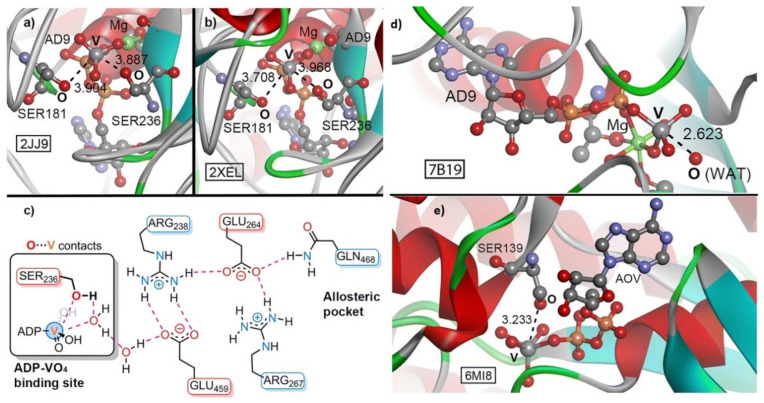
Partial views of the X-ray crystal structure of 2JJ9 (**a**) and 2XEL (**b**) with indication of the O⋯V contacts (distances in Å). Amino acids and water molecules involved in the noncovalent network of interactions (highlighted in purple) connecting the allosteric pocket and the ADP-vanadate binding site in the 2JJ9 structure (**c**). Shown in (**d**,**e**) are the partial views of the X-ray crystal structures of 7B19 and 6MI8 structures with indication of the O⋯V contact. Distances in Å. Reproduced from ref. [339].

In an insightful study, the same authors showed the crucial role played by π-holes in nitro aromatic ligands, specifically highlighting the positive electrostatic potential on the nitrogen atom of the nitro group [108]. They provided compelling evidence for the presence of “π-hole interactions” between the nitro moiety of these ligands and the lone pairs in protein structures, with interaction energies around −5 kcal mol^−1^. Additionally, the group explored another type of interaction, V⋯O/N, focusing on the σ-hole present in vanadium atoms within DP metavanadate (VO_4_) and ADP orthovanadate (VO_5_) [339]. These σ-holes interact with lone pairs on protein residues (e.g., serine, glutamate, histidine), backbone carbonyl groups, and water molecules. Their detailed PDB inspection revealed 32 structures containing metavanadate and orthovanadate moieties, with 25 of these showing significant V⋯O/N noncovalent contacts. The strength and directionality of V⋯O interactions in, for example, four PDB (Figure 39a–e) offers a theoretical perspective on the potential significance of these noncovalent contacts.

Traditionally, biologically significant anions like NO_3_^−^ have been seen as typical electron donors. However, a recent study [109] showed that NO_3_^−^ has an anisotropic charge distribution. The charge density on the nitrogen is minimal and can be spread over a larger area, effectively transforming it into a Lewis acidic site that can interact favorably with electron-rich partners, especially when the O-atoms in NO_3_^−^ interact constructively with water or NaCl. For example, N’s sterically accessible π-hole in [LiNO_3_·2H_2_O] is positive with a strength of +25 kcal mol^−1^, which in principle, makes it capable of forming electrostatically-driven nitrogen-centered pnictogen bonds [53,119].

**Figure 40:**
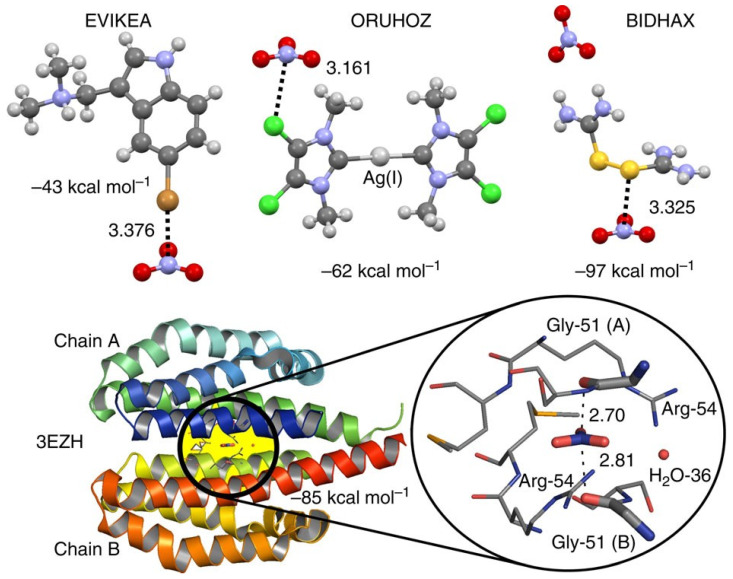
(**Top**) Illustration of N’s π-hole driven NO_3_^−^ complexes found in some crystal structures deposited in the CSD; in each case, the intermolecular distances (Å), the complex binding energy (kcal mol^−1^), and CSD reference codes in uppercase letters are shown. (**Bottom**) An example from the PDB with a zoom-in of the nitrate ligand’s binding pocket (residues ≤ 4 Å displayed). All these selected fragments were computed at the BP86-D3/def2TZVP level of theory leading to the indicated energies (dominated by charge compensation). Color code: carbon–grey; hydrogen–white; nitrogen–blue; oxygen–red; sulfur–yellow; chlorine–green; bromine–brown; and silver–light grey. Reproduced from ref. [109].

Because a bare yet semi-nucleophilic π-hole in NO_3_^−^ interacts with negatively charged sites in entities with which it interacts, as found in many crystal structures, it has been proposed that these interactions are formally (pseudo) anti-electrostatic interactions found in the solid state [340,341]. To develop this concept, the authors modeled fragments extracted from the structures of various salts (Figure 40, top), where the −O_3_N(π-hole)···Y (Y = Br, Cl, S; electron-density rich) distances are smaller than the sum of the van der Waals radii of the respective atomic basins and the interactions are energetically favorable (see interaction energies in kcal mol^−1^). Similarly, in the protein structure 3EZH (ref. [29]; Figure 4, bottom), two isostructural chains (A and B) encircle a central nitrate. The NO_3_^−^ anion is hydrogen-bonded to two arginine residues (Arg-54), with N···N distances of approximately 3 Å. Moreover, the carbonyl oxygen atoms of two glycine residues (Gly-51) appear to interact with the nitrate ligand’s π-hole. The interatomic N···O distances of 2.70 and 2.81 Å are within the sum of the van der Waals radii for N and O atoms (3.07 Å), providing evidence for the existence of these π-hole pnictogen-bonded interactions.

Parker et al. [342] designed artificial DNA base pairs by substituting hydrogen atoms by halogens, modifying the G, C, A and T nucleosides. This methodology proved effective in the rational design of artificial proteins and nucleotides. The resulting structures were energetically stable and coplanar, with the stability of the halogenated base pairs within 2 kcal mol^−1^ of their hydrogen-bonded counterparts. Among the halogens tested (Cl, Br, and I), bromine was found to be the most suitable for inclusion in biological systems due to its optimal balance of polarizability and steric compatibility. Gomila and colleagues [343] noted that halogenation of nucleic acids has been a common practice for decades, primarily to aid in X-ray diffraction analysis. Incorporating halogen atoms into DNA/RNA bases not only alter the electronic distribution but also extends the range of noncovalent interactions beyond the range of traditional hydrogen bonds (HB). Their analysis of the PDB identified 187 structures involving halogenated nucleic acids (either unbound or protein-bound) where at least one base pair was halogenated. The authors subsequently investigated the strength and binding preferences of various halogenated base pairs using RI-MP2/def2-TZVP level calculations. The observed bonding modes, along with a range of interaction energies, were consistent with the electrostatic potential of hydrogen and halogen atoms. This alignment helped to explain the stability of various paired configurations, whether they involved Watson-Crick base pairing or Hoogsteen base pairing.

Relevant to protein-drug recognition and its burgeoning applications in biomedical science, the enigmatic tetrel bonds offered by the carbon center of CF_3_ groups within certain molecular structures have been scrutinized through a sophisticated blend of PBD and quantum mechanical calculations at the RI-MP2/def2-TZVPD level of theory [344]. An extensive survey of the PDB found 419 X-ray crystallographic structures, featuring organic ligands with CF_3_ groups and adjacent electron-rich atoms (A = N, O, and S), unveiled a fascinating array of CF_3_⋯A tetrel bonds. The occurrence of these interactions in the crystals were identified based on their directional properties and intermolecular distances, with the distance (C⋯A) between the CF_3_ carbon (C) and the Lewis base (A = N, O, and S) between 2.5 Å and 4 Å. The electronegative atoms considered belonged to amino acids and ligands, and interactions with solvent molecules were not taken into account. The angle criterion used for the search was 120° ≤ ∠X–C⋯A ≤ 180°, where X is any atom attached to the C of the CF_3_ group. Quantum mechanical calculations gave stabilization energies between −0.3 and −5.3 kcal mol-1, and the σ-hole of the carbon atom was shown to be correlated with the basis set superposition corrected binding energy values for several complexes, with the latter shown to be a good predictor of the tetrel bond interaction strength. Hammett regression plots were demonstrated for some tetrel bonded complexes involving meta- and para-substituted benzene derivatives, providing insight into the effects of substituents on these bonds.

**Figure 41:**
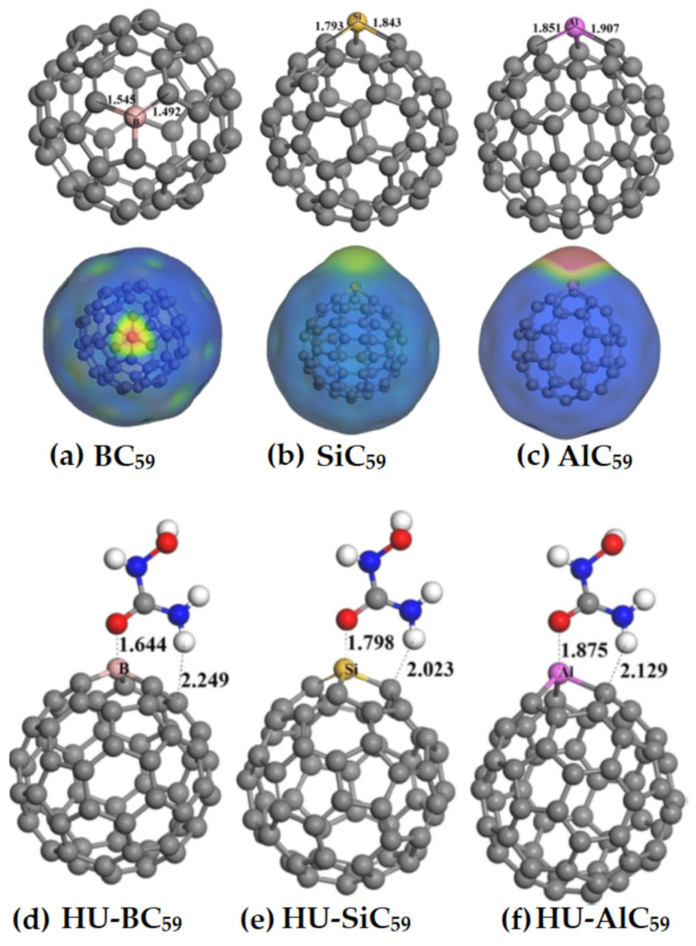
DFT relaxed structures (Top) and MESP plots (Bottom) of (**a**) BC_59_, (**b**) SiC_59_, and (**c**) AlC_59_, where the colors blue and red in the MESP plot represent negative and positive regions, respectively [345]. Shown in (**d**–**f**) are the relaxed geometries of HU⋯BC_59_, HU⋯SiC_59_, and HU⋯AlC_59_ complexes, respectively. Bond distances are in Å. The PBE functional together with a double numerical basis set including d-polarization functions (DNP) was used.

Wang et al. [345] conducted a theoretical study on the interaction between the anticancer drug hydroxyurea (HU) and both pristine C_60_ and heterofullerene MC_59_ (M = B, Si, Al), with the latter systems depicted in Figure 41a–c (top). HU, also known as hydroxycarbamide, is a potent anticancer agent used to treat head and neck cancer, breast cancer, and chronic myelogenous leukemia [346,347], although it can cause side effects such as rashes, leukopenia, and leg ulcers. Their findings reveal a notable contrast in how HU interacts with pure C_60_ versus the doped fullerenes. On pristine C_60_, HU exhibits a weak interaction with an adsorption energy of −6.2 kcal mol^−1^, indicating that HU is primarily physically adsorbed through hydrogen bonding (HU)H⋯C_60_. Consequently, pristine C_60_ is not suitable for HU drug delivery. In stark contrast, HU shows chemisorption on the doped fullerenes BC_59_, SiC_59_, and AlC_59_ (Figure 41e–g), with an adsorption energy of −25.87, −36.14, and −50.13 kcal mol^−1^, respectively. The appreciable adsorption energies were attributed to the highly electrophilic nature of the doped species (B, Si, and Al), revealed by a MESP analysis (Figure 41a–c (bottom)). Because of this, it was suggested that BC_59_, SiC_59_, and AlC_59_ may be promising candidates for drug delivery systems involving HU.

To evaluate the effectiveness of the MESP model in interactive drug design, and its time-intensive nature, Rathi and colleagues [4] have innovatively employed a graph convolutional deep neural network (DNN) model to efficiently generate MESP surfaces from high-quality quantum mechanical data. Their advanced DNN-fp model, which significantly outperforms the AM1-BCC model, produces electrostatic potential surfaces within mere seconds, facilitating its application in interactive drug design. This was tested by evaluating the efficacy of the DNN-fp model by comparing the generated ligand ESPs with experimentally measured molecular properties, including hydrogen bond basicity, acidity constants, and two instances of protein–ligand binding affinity. In all cases, a robust correlation was observed between the DNN-fp ESP values and the experimental data. Notably, the correlation coefficients for the DNN-fp model were generally on par with those derived from DFT-based electrostatic potentials and superior to those obtained using AM1-BCC based electrostatic potentials.

## 9. The MESP Model Limitations and Complementary Approaches

Activation energies (*E_a_*) for methane’s C–H bond activation by neutral and cationic Pd(II)–methoxy complexes correlate quantitatively with electrostatic descriptors of N-heterocyclic carbene (NHC) ligands [348]. In particular, *V_min_* (global electrostatic minimum), and *V_C_* (electrostatic potential at the carbene carbon nucleus) quantify ligand electron-donating ability and track changes in *E_a_*. These descriptors therefore provide a concise predictive framework for assessing trans influence, especially in systems with minimal secondary interactions or steric hindrance.

The descriptors such as *V_S,max_* correlate with interaction strength in hydrogen- and halogen-bonded systems, but they remain indirect indicators and do not encompass dispersion or many-body effects [349]. Some have demonstrated that MESP alone may fail to predict binding energies or accurately rank interaction strengths in systems where electrostatic terms are not the dominant contribution; in many cases such anomalies arise because secondary interactions modify primary interactions and therefore influence interaction energies.

Grimmel et al. [350] examined a diverse set of localized lone-pair donors, π-systems, and aromatic sites to evaluate whether local minima of the molecular electrostatic potential can serve as indicators of protonation sites. Their dataset shows that more negative MESP minima generally correspond to more favorable protonation at localized lone-pair centers, supporting the use of MESP as a qualitative predictor of proton affinity. However, the relationship is not universally linear and becomes less reliable in delocalized π-systems and multifunctional molecules, where structural relaxation, resonance stabilization, and polarization effects contribute significantly to protonation energies. Accordingly, the MESP minima provide a useful first-order heuristic for identifying likely protonation sites, but they do not fully capture all thermodynamic contributions governing protonation.

Brink and co-workers [349] examined 20 halogen-bonded complexes of the types R–Br···Br^−^ and R’–C≡C–Br···Br^−^ (R = substituted methyl group) using the M06-2X/6-311+G(d,p) level of theory. A point-charge model, in which Br^−^ is represented by a point charge in the electronic Hamiltonian, reproduced the halogen-bond energies with remarkable accuracy: the interaction energies correlated linearly with the point-charge interaction energy with a slope of 0.88, zero intercept, and *R*^2^ = 0.9995. Decomposition of the interaction energy shows that polarization contributes substantially to bond strength in addition to electrostatics. Across the data set, electrostatic energies range from −4 to −18 kcal mol^−1^, while polarization energies range from −4 to −10 kcal mol^−1^, highlighting the significant role of polarization in halogen-bond stabilization within the point-charge framework and obviating the need to invoke charge transfer as a separate contribution.

Although the MESP model is widely used for qualitative and semi-quantitative analysis of noncovalent interactions, it primarily reflects the electrostatic component and does not explicitly capture dispersion or many-body correlation effects especially in low-polarizable fluorine-based systems [32,195]. These effects also dominate interaction energies in dispersion-bound systems such as noble gas medicated complexes [351] and π–π stacking [352,353], as well as in counterintuitive [107,187,193] and anti-electrostatic σ- and π-hole interactions [32,196,200,354].

It was also shown that electrostatic interactions are neither necessary nor sufficient to explain the parallel-displaced geometries of π-stacked systems. The classical quadrupolar model [355]—described as “neat, simple, and wrong”—fails to capture the significant charge penetration that occurs at typical nonbonded contact distances, including π-stacking separations of about 3.4–3.8 Å [356]. This charge penetration contributes to interaction stabilization in ways not described by a purely multipolar electrostatic picture, and may be understood from the work of Wheeler and coworkers [352,353,355,357,358]. Similarly, attractive interactions between like-charged species can arise from local electronic environments, or solvent-mediated effects, that render specific regions of an anion locally acidic, thereby enabling binding despite overall electrostatic repulsion. This phenomenon has been demonstrated in studies by Schreiner [110].

**Figure 42:**
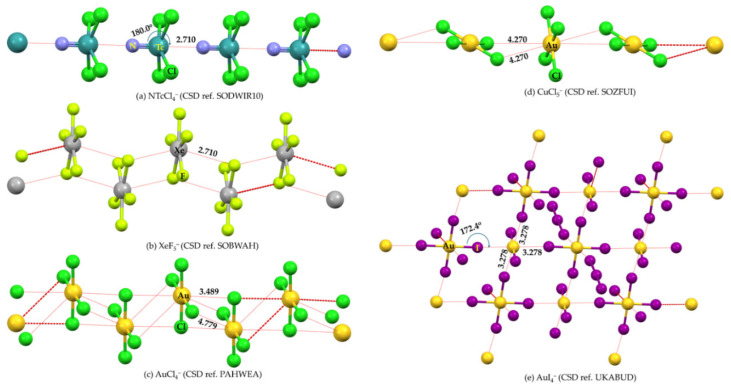
(**a**–**e**) Illustration of selected close contacts in inorganic anionic fragments observed in the crystalline phase. Organic counterions and auxiliary groups have been omitted for clarity. Selected intermolecular distances and angles are in Å and degrees, respectively. Dotted red lines indicate intermolecular close (and hanging) contacts. The CSD reference is shown for each case.

Like-charged anions can nevertheless form stabilized assemblies despite the electrostatic repulsion expected from their overall charge. Many of these anionic assemblies are contained in organic–inorganic hybrid adducts or cocrystalline salts. Representative examples include (C_12_H_24_CsO_6_^+^)_n_,n(Cl_4_NTc^–^) [359], (C_4_H_12_N^+^,F_5_Xe^–^) [360], (C_5_H_12_NO^+^,AuCl_4_^–^) [361] (C_6_H_5_Cl_3_P^+^,AuCl_4_^–^) [362] and 2(C_8_ H_22_ N_2_ ^2+^)∙(AuI_2_^–^,Au I_4_^–^,2(I_3_^–^)) [363]. Figure 42a–e feature intermolecular distances consistent with possible close contacts that give rise to geometric architectures in different dimensions. In such systems, stabilization arises from polarization, charge-transfer, and dispersion effects rather than simple Coulombic attraction [364].

Holthoff et al. [200] demonstrated halogen-bonded complexes in which anionic donors engage in attractive interactions with other anions even though their electrostatic potential surfaces are uniformly negative, indicating that the binding originates from non-electrostatic contributions. Further studies of anion–anion adducts, including X_4_Tr^−^⋯TrX_4_^−^ complexes (Tr = B, Al, In, Tl; X = F, Cl, Br) [364] and complexes such as MCl_3_^−^⋯pyridine/CN^−^ (M = Be, Mg, Ca, Sr, Ba) [365], reveal anti-electrostatic behavior in the gas phase.

Cl_4_Tr^−^⋯CN^−^ adducts (Tr = Al, Ga, In, Tl) are endothermic in the gas phase with binding energies of approximately 37–40 kcal mol^−1^, yet become stabilized in aqueous solution with binding energies between −5 and 15 kcal mol^−1^ [364]. Similar stabilization trends are observed in stacked (MX_3_^−^)_2_ dimers (M = Zn, Cd, Hg; X = Cl, Br, I) [365], AeX_5_^−^ species interacting with bare anions (F^−^, Cl^−^, CN^−^) [366], and dimers of [Au(CN)_4_]^−^, [AuCl_4_]^−^, [AuBr_4_]^−^, [AuI_4_]^−^, and [AuO]^−^ with small anions such as F^−^, Cl^−^, and CN^−^ [367,368].

The examples above demonstrate that anion–anion interactions, though counterintuitive from a purely electrostatic viewpoint, can be stabilized through solvation and noncovalent electronic effects, underscoring the necessity of energy decomposition analyses to capture their full physical origin. Nevertheless, it must be emphasized that in real crystals such situations do not occur in isolation. Anions do not self-assemble exclusively with other anions; rather, they are always accompanied by organic or inorganic cations. In such systems, other noncovalent interactions—most commonly hydrogen bonding, electrostatic ion pairing, or related contacts—are generally primary and bring the anions into proximity, with the observed anion–anion contacts arising as secondary consequences of the overall packing arrangement.

Table 3 summarizes MP2/aug-cc-pVDZ level *V_S,max_* of isolated TrX_4_^−^ anions together with anion⋯anion triel bond distances, interaction and binding energies of (TrX_4_^−^)_2_ homodimers in water and van der Waals radii sums for noncovalent contacts, with BSSE-corrected energies in parentheses [364]. The data show that fluoride homodimers exhibit the strongest interactions, characterized by short triel bond distances (~2.0–2.3 Å) and highly negative interaction energies (up to ~−40 kcal mol^−1^, BSSE corrected), reflecting strong triel bonding and significant stabilization even in aqueous solvent. In contrast, chloride and bromide systems display longer bond distances (>3 Å) and much weaker interaction energies (generally −2 to −8 kcal mol^−1^), consistent with reduced charge density and dispersion-dominated contacts. *V_S,max_* values differ only marginally between gas and solvent phases, showing that solvation has little effect on surface electrophilicity; however, comparisons with van der Waals radii sums indicate mixed behavior—some dimers exhibit contacts shorter than the vdW limit consistent with genuine noncovalent interactions, whereas others (notably BF_4_^−^, BCl_4_^−^, AlCl_4_^−^, GaCl_4_^−^, AlBr_4_^−^ and GaBr_4_^−^) display distances comparable to or exceeding the vdW sum, suggesting weaker, dispersion-dominated contacts.

The inverse fit using the model E=a(1/r)+b  captures the overall trend that higher 1/r values correspond to more negative interaction energies, consistent with stronger interactions at shorter distances. The relatively high $R^{2}\approx 0.9\;$ indicates a meaningful inverse dependence, though significant scatter and deviations from a perfect inverse law remain. These deviations reflect chemical specificity—F-containing adducts behave differently from Cl- and Br-based systems—and additional polarization and charge-transfer effects that modulate the energies beyond simple geometric dependence. Thus, the inverse model serves as a useful first-order approximation but not a complete predictive description of interaction strength.

The energy decomposition analysis (EDA) [369,370] often complements the MESP model by partitioning interaction energies into electrostatic, polarization, dispersion, and exchange–repulsion components in a method-dependent manner. Thus, MESP and EDA address different but complementary aspects of noncovalent interactions: MESP provides qualitative insight into preferred interaction sites, whereas EDA offers quantitative (though scheme-dependent) energy decomposition. Neither method alone delivers a complete or uniquely defined description of bonding.

It is also important to recognize that multiple EDA frameworks exist, including supermolecular schemes such as Morokuma–Kitaura EDA [371], Extended Transition State–Energy Decomposition Analysis (ETS-EDA) [372], and ALMO-based variants [372,373]; perturbative approaches such as symmetry-adapted perturbation theory (SAPT) [372,374,375] and hybrid methodologies [376,377]. While these methods have been successfully applied to hydrogen bonds, halogen bonds, and other σ-hole interactions, their results should be interpreted as outputs of a methodological decomposition rather than as evidence of uniquely defined physical forces.

As emphasized by Politzer and coworkers [378], quantities such as charge transfer, exchange, and polarization are path-dependent constructs arising from specific partitioning schemes. Different energy decomposition analyses may therefore yield divergent numerical values and even contradictory interpretations for the same system, as shown for complexes formed of methyl halides and trifluoromethyl halides with formaldehyde [379,380]. This does not invalidate decomposition as an investigative technique, but it requires careful qualification: decomposed components illuminate aspects of the interaction within a chosen framework, yet they are not physical observables and cannot be assigned independent physical reality. The Hellmann–Feynman theorem remains fully compatible with this perspective by attributing the net force to the interaction between nuclei and the total electron density rather than to arbitrarily separated contributions.

Furthermore, Politzer and coworkers emphasize that halogen bonding can be understood primarily in terms of electrostatic and polarization effects arising from the interaction of the electron density with the nuclei [378]. Although energy decomposition analyses sometimes report a “charge-transfer” contribution, its magnitude and even its definition depend strongly on the chosen decomposition scheme and reference states. In many formulations, charge transfer is not an independent driving force but rather a manifestation of polarization and the redistribution of electron density that accompanies complex formation. From this viewpoint, the strength of any noncovalent interaction, including counterintuitive interactions, is driven by sites of opposite polarity on interacting atoms can be rationalized without invoking a distinct charge-transfer interaction; electrostatics and polarization suffice to explain the stabilization, while decomposed charge-transfer terms serve as interpretative constructs. This may be consistent with the Hellmann–Feynman framework [378].

ETS-NOCV (extended transition state–natural orbital for chemical valence) analysis is a method that decomposes interaction energies into components such as electrostatics, exchange, charge transfer, and orbital interactions, thereby providing insight into bonding mechanisms beyond simple electrostatic descriptors. However, as noted by Mitoraj et al. [381,382], the approach involves some arbitrariness in the definition of molecular subsystems and energy partitions. ETS-NOCV visualizes orbital interactions through charge-density deformation analysis, linking electron flow to donor–acceptor and polarization effects. The resulting decomposition is method-dependent and not unique; NOCV contributions should therefore be interpreted as qualitative or semi-quantitative descriptors of interaction mechanisms rather than scheme-independent or directly measurable forces. While ETS-NOCV effectively reveals polarization and charge-transfer pathways, it does not provide a universally defined separation of interaction energies.

Charge-density–based topological approaches such as quantum theory of atoms in molecules (QTAIM) [383] further complement MESP by characterizing bond critical points and electron density distributions, offering an alternative description of bonding that is not restricted to surface potentials [25,124]. However, several studies report limitations of QTAIM in weak interaction regimes [125,384,385], including cases such as intramolecular interactions in aminoethanol [385] and other systems where expected attractive interactions are not detected or where unexpected interactions are identified, as in the crystalline systems CF_3_ICl_2_ and CF_3_IClF [384].

**Figure 43:**
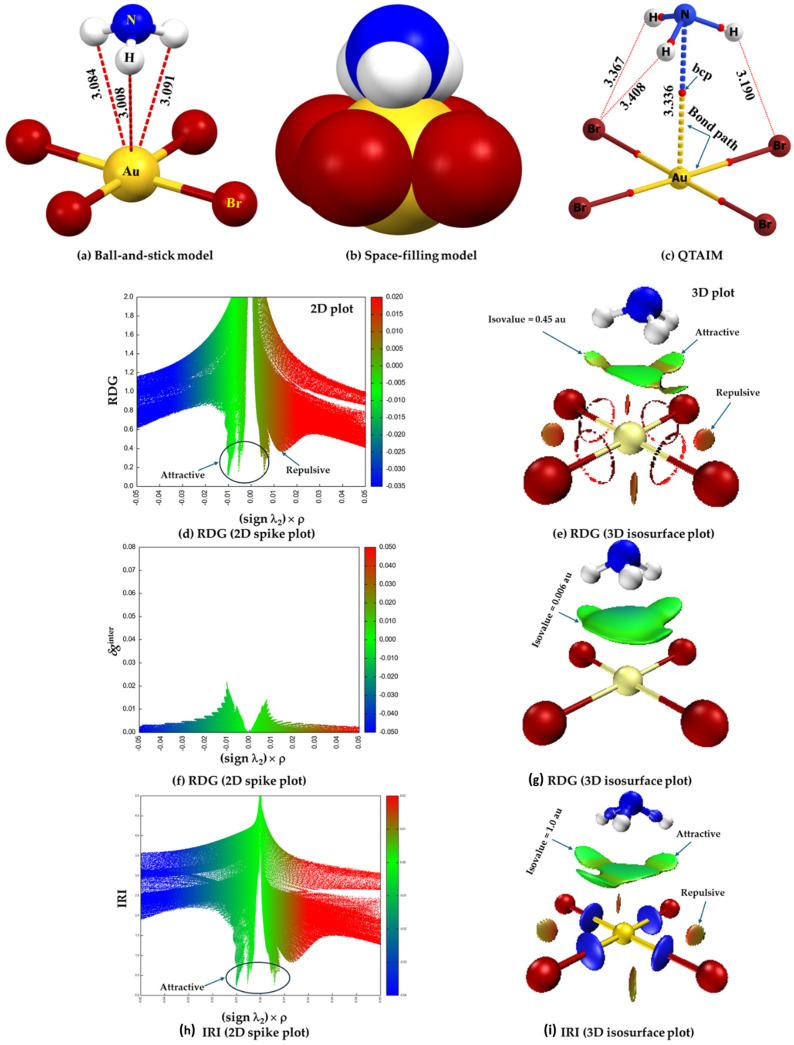
(**a**) Ball-and-stick representation of the optimized geometry of Br_4_Au^−^···NH_3_ computed at the M06-2X/def2-TZVPPD level; (**b**) corresponding space-filling model; (**c**) QTAIM molecular graph of the complex. Panels (**d**,**f**,**h**) present the 2D spike plots obtained from the RDG, IGM, and IRI analyses, respectively, while panels (**e**,**g**,**i**) show the corresponding 3D isosurface maps. Bond lengths in (**a**,**c**) are given in Å. The red dotted lines in (**c**) are manually drawn to highlight the H···Br hydrogen-bond interactions.

Another illustrative example emerged from this work concerns the Br_4_Au^−^···NH_3_ anion–molecule complex formed between Br_4_Au^−^ and NH_3_ (M06-2X/def2-TZVPPD). From the geometric perspective alone (Figure 43a), or based on the concept of the space filling model (Figure 43b), the Au···H and Au···N interactions between the Au center and a hydrogen and nitrogen atoms of NH_3_ is best described as a weak noncovalent interaction, primarily electrostatic in origin, arising from attraction between the partially positive hydrogen and nitrogen of NH_3_ and the negatively charged Au in Br_4_Au^−^ fragment, thereby dominated by Coulombic and polarization effects, with at most minor charge-transfer contributions, and is fully concordant with the MESP model.

QTAIM analysis, however, identifies an N···Au interaction characterized by a bond path and bond critical point (bcp) (Figure 43c). This leads to an interpretation that arises from interaction between the electron-deficient region on nitrogen (along the extension of the *C_3v_* axis opposite the lone pair direction) and a negative *d_π_*-hole on gold. However, this topological description does not fully account for additional stabilizing interactions in the complex (e.g., N···Au, H···Au and H···Br). In particular, the hydrogen atoms of NH_3_ possess *s*-type σ-holes that are oriented toward the electron-rich bromide and gold sites, giving rise to multiple H···Br and H···Au close contacts. These secondary/primary interactions are not equivalently reflected in the bond-path topology, highlighting limitations of relying solely on QTAIM criteria to characterize the full interaction pattern in such systems.

Wick and Clark [386] emphasized the stringent space-partitioning framework underlying QTAIM, drawing attention to conceptual limitations inherent in its topological definitions. In particular, they showed that bcps may arise artificially under the influence of an external polarizing field or even upon addition of a single non-bonded atom, thereby questioning the reliability of bcps and associated bond paths as unequivocal indicators of chemical bonds or attractive intermolecular interactions. This interpretation aligns with the conclusions of Foroutan-Nejad et al. [387], who likewise argued that bond paths should not be equated with chemical bonds. Nevertheless, QTAIM continues to be widely employed to elucidate bonding features across diverse chemical systems, including recent applications reported by Schreiner [388]. Collectively, these examples indicate that although QTAIM may not provide definitive criteria in regimes dominated by weak interactions, it remains a valuable complementary framework when applied with appropriate methodological caution and chemical judgment.

Using SAPT, Scheiner [388] analyzed a series of halogen, chalcogen, pnicogen, and tetrel bonded complexes and demonstrated that the electrostatic component constitutes more than half of the total attractive interaction energy. In particular, the extrema of the MESP—the positive maximum on the Lewis acid (e.g., Cl on FCl, Si on FH_3_Si, Te on TiH_2_, and FH_2_As, etc.) and the negative minimum on the Lewis base (e.g., NH_3_)—offer a physically intuitive measure of interaction propensity. Although the simple product of these extrema reproduces the electrostatic term only with moderate quantitative precision, it nevertheless reflects the principal driving force behind complex formation. While induction and dispersion also contribute meaningfully to stabilization—and while QAIM-based descriptors may correlate well with total interaction energies—MESP retains a particularly conceptual value as a first order approximation because it connects interaction strength to fundamental electrostatic characteristics of the unperturbed monomers.

Several isosurface-based topological approaches have been proposed in recent years, including reduced density gradient (RDG) [389], independent gradient model (IGM) [390,391,392,393], interaction region indicator (IRI) [394] analysis methods, in which covalent and noncovalent interactions of different strengths can be visualized through 2D spectra (sign(*λ*_2_) × *ρ* versus RDG, *δg*^inter/intra^ and IRI), as well as the 3D isosurfaces between interacting atomic basins. These approaches provide a visual and qualitative means of identifying regions of weak intermolecular interactions by analyzing low-gradient regions of the electron density, revealing noncovalent interaction zones such as hydrogen/halogen bonds, van der Waals contacts, and steric repulsion through isosurfaces correlated with electron-density features. Such representations offer intuitive spatial insight beyond purely energetic descriptions. However, RDG and related isosurface methods do not quantify interaction energies or provide scheme-independent measures of bonding strength; their interpretation remains qualitative and dependent on the chosen isosurface thresholds (such as isovalues) and color-mapped regions. Moreover, distinguishing weak attractive interactions from numerical noise in low-density regions can be challenging, particularly in complex or highly delocalized systems.

In an RDG versus sign(*λ*_2_) × *ρ* plot, the most pronounced spikes generally appear at positive sign(*λ*_2_) × *ρ* values (i.e., sign(*λ*_2_) > 0), corresponding to steric repulsion or Pauli exchange, where electron densities strongly overlap and the density gradient varies rapidly; these regions are typically represented by red spikes. Attractive interactions, such as hydrogen/halogen bonding and related noncovalent contacts, appear in the negative sign(*λ*_2_) × *ρ* region (sign(*λ*_2_) < 0), commonly shown as blue/green spikes, as illustrated for the Br_4_Au^−^···NH_3_ complex (Figure 43d). Weak dispersive (van der Waals) interactions are located near sign(*λ*_2_) × *ρ* ≈ 0 at low RDG values and usually manifest low-intensity features rather than sharp spikes; in the present case, such contributions are minimal (isosurfaces in brown). These spikes are consistent with the corresponding isosurface representation shown in Figure 43e, where green and brown isosurfaces denote noncovalent attractive interactions (with green typically associated with stronger attractive contacts and brown with weaker dispersive interactions around sign(*λ*_2_) × *ρ* ≈ 0), while the red regions correspond to sign(*λ*_2_) > 0 and indicate steric repulsion.

In an IGM analysis, *δg^inter^* versus sign(*λ*_2_) × *ρ* scatter plot (where *δg^inter^* incorporates intermolecular contributions), the characteristic spikes arise from regions of intermolecular interaction and are distributed on either side of sign(*λ*_2_) × *ρ* ≈ 0, reflecting attractive contact regions within the complex. The height of a spike reflects the magnitude of *δg^inter^* and therefore correlates with the strength of the corresponding interaction: the larger the height of the spike, the stronger the interaction. As shown in Figure 43f for the Br_4_Au^−^···NH_3_ complex, the interaction between N and Au, as well as that between H and Au, appears stronger (with a peak height up to ~0.023 a.u. in the negative sign(*λ*_2_) × *ρ* region) than the longer H···Br contacts (with a peak height up to ~0.015 a.u. in the positive sign(*λ*_2_) × *ρ* region), consistent with the more centrally located, flatter isosurface for the N···Au and H···Ag interaction compared to the narrower isosurfaces associated with the long-ranged H···Br interactions.

IRI also reveals both covalent and weak interaction zones through smooth isosurfaces (commonly at an isovalue of 1.0 or greater). Its definition relies only on the electron density and its gradient, making it computationally simpler and less expensive than alternatives such as DORI (density overlap region indicator) descriptor [395,396], which additionally requires the Hessian of the density. Moreover, IRI generally provides smoother and less noisy isosurfaces delineating interaction regions between atomic basins (blue for inter-fragment regions and green for intra-fragment regions), offering a clearer graphical representation of bonding and weak interactions than RDG- or IGM-based NCI analyses; these methodologies, along with related implementations, are available in software such as Multiwfn [397,398] and other computational platforms [392,399]. The improved visualization is reflected in both the spike and isosurface plots shown in Figure 43g,h. Consequently, isosurface-based analyses may be best regarded as complementary visualization tools to energetic and topological methods rather than standalone descriptors of interaction strength. This perspective aligns with the view that noncovalent interactions originate from the overall electronic structure of the system—captured by the total energy and electron density—while decomposed contributions serve as useful interpretative tools rather than fundamental observables.

## 10. Conclusions and Outlook

Over the past decade, numerous studies have highlighted the power of the MESP model in chemical analysis, demonstrating its ability to rationalize reactivity, noncovalent interactions, and supramolecular behavior across diverse chemical systems. Yet, the model’s limitations—sometimes underreported—underscore the need for careful application and interpretation. Seminal works by Scheiner and Murray et al. illustrate the subtleties of “counterintuitive” versus “intuitive” noncovalent interactions, particularly those involving σ- and π-holes, emphasizing that electrostatic extrema are not inherently positive and can be highly anisotropic. This review has underscored the practical utility of the MESP model in identifying electrophilic and nucleophilic domains, rationalizing molecular recognition, and predicting supermolecular organization, while also correcting widespread misconceptions regarding noncovalent chemistry.

The MESP model demonstrates broad applicability across chemistry, catalysis, materials science, chemical engineering, biology, and drug design. It provides a direct window into electrostatic landscapes that govern molecular recognition, reactivity, and noncovalent interactions. Nonetheless, the model’s predictive accuracy is highly sensitive to the level of electronic structure theory employed and to the choice of isodensity envelope. Systems containing highly electronegative or weakly polarizable atoms may exhibit underestimated or misrepresented anisotropies. For instance, in fluorine-substituted molecules such as CF_4_, conventional isodensity surfaces can obscure lateral potential minima near fluorine atoms and fail to highlight the electropositive extension along C–F bonds. These limitations reflect methodological sensitivity rather than conceptual failure; adopting an isodensity threshold of ≥0.0014 a.u., as recommended by Bader et al. [66], recovers these subtleties and enables precise mapping of critical points and noncovalent interaction regions. This careful calibration ensures MESP analyses align with chemical intuition, capturing nuanced electrostatic features in complex or counterintuitive bonding environments.

Looking forward, the MESP model holds promise for next-generation computational chemistry applications. Integrating MESP-derived electrostatic descriptors with machine-learning algorithms and high-throughput screening can accelerate the rational design of catalysts, functional materials, and drug candidates by predicting reactivity and binding affinity from first principles. Moreover, extending MESP calculations to include time-dependent effects, solvent interactions, and dynamic conformational ensembles will enhance predictive accuracy for real-world chemical systems, enabling quantitative forecasts of electron transfer, photophysical properties, and supramolecular assembly. Ultimately, these developments position the MESP model not only as a descriptive tool but also as a predictive framework capable of guiding experimental design across multiple domains of chemistry and materials science.

## 11. Software and Computational Details

Computations of molecular entities, where required, were performed using Gaussian 16 [400], with the theoretical methodology described in the relevant sections. Molecular graphs within the Quantum Theory of Atoms in Molecules (QTAIM) [383] framework were generated using AIMAll 19.10.12 [401]. Molecular electrostatic surface potential (MESP) plots and extrema of potential were analyzed using VMD 2.0 [402], Multiwfn 2.1.2 [398] and AIMAll [401]. Crystal structures were examined using the Cambridge Structural Database (CSD 6.01) [127,128,129], and Mercury software 4.0 [403] was used for visualization and preparation of the graphs presented in this work.

## Figures and Tables

**Table 1:** CCSD(T)(F12c)/cc-pVDZ-F12 level intermolecular dissociation energies *D*_e_ for some selected complexes formed with some selected bases B ^a^.

Lewis Base B	O_2_C⋯B/kcal·mol^−1^	O=N=N⋯B/kcal·mol^−1^	S=C=S⋯B/kcal·mol^−1^
OC	1.17	1.10	0.71
HCCH	2.11	1.94	1.02
HCN	2.19	1.87	1.47
H_2_O	3.29	2.98	1.91
H_2_S	1.87	1.73	1.26
H_3_N	3.47	2.82	2.24
H_3_P	1.50	1.41	1.61

^a^ Values in [115] were reported in kJ mol^−1^ (1 kJ mol^−1^ = 0.239006 kcal mol^−1^).

**Table 2:** MP2/aug-cc-pVTZ level intermolecular distances (*r*) and CCSD(T)/aug-cc-pVTZ//MP2/aug-cc-pVTZ level interaction energies (*E^int^*) for selected π-hole tetrel-bonded complexes formed by TrBH_2_ (Tr = Si, Ge, Sn, Pb) [79].

Complex	*r*/Å	*E^int^*/kcal·mol^−1^
SiH_2_···N_2_	2.042	−6.17
GeH_2_···N_2_	2.211	−4.08
SnH_2_···N_2_	2.549	−3.48
PbH_2_···N_2_	2.643	−3.03
SiH_2_···HCN	1.951	−17.53
GeH_2_···HCN	2.087	−13.14
SnH_2_···HCN	2.373	−11.13
PbH_2_···HCN	2.485	−9.21
SiH_2_···CO	1.889	−23.39
GeH_2_···CO	1.989	−16.07
SnH_2_···CO	2.347	−9.56
PbH_2_···CO	2.498	−7.16
SiH_2_···OC	2.581	−2.16
GeH_2_···OC	2.593	−2.14
SnH_2_···OC	2.769	−2.14
PbH_2_···OC	2.785	−2.11
SiH_2_···C_6_H_6_	2.381	−10.18
GeH_2_···C_6_H_6_	2.446	−8.78
SnH_2_···C_6_H_6_	2.700	−7.79
PbH_2_···C_6_H_6_	2.746	−6.81

**Table 3:** MP2/aug-cc-pVDZ level maxima (*V_S,max_*) of isolated TrX_4_^−^ anions (kcal mol^−1^), anion⋯anion triel bond distances (r(Tr⋯X) in Å), and interaction and binding energies (*E_int_* and *E_b_* in kcal mol^−1^) of (TrX_4_^−^)_2_ homodimers in water (solvent). van der Waals radii sums (Å) for noncovalently bonded atom contacts are also reported, and values in parentheses correspond to basis-set superposition error (BSSE)–corrected energies [364].

Anion	*V_S,max_* (Gas)	*V_S,max_* (Solvent)	*E_int_*	*E_b_*	*r*(Tr⋯X)	vdW Radii Sum
BF_4_^−^	−116.2	−116.3	−0.22 (0.66)	−0.23 (0.65)	4.047	3.37
AlF_4_^−^	−85.4	−85.6	−27.66 (−23.22)	−0.64 (3.80)	2.026	3.71 (cov: 1.78)
GaF_4_^−^	−78.8	−78.5	−28.79 (−21.20)	−7.24 (0.34)	2.093	3.78 (cov: 1.79)
InF_4_^−^	−57.9	−55.5	−39.66 (−33.65)	−22.04 (−16.03)	2.187	3.89 (cov: 1.99)
TlF_4_^−^	−62.1	−59.4	−34.68 (−26.57)	−20.48 (−12.37)	2.259	3.93 (cov: 2.02)
BCl_4_^−^	−82.7	−81.7	−1.96 (−0.77)	−1.96 (−0.77)	4.481	3.73
AlCl_4_^−^	−80.4	−81.0	−2.58 (−0.83)	−2.55 (−0.80)	4.414	4.07
GaCl_4_^−^	−79.0	−79.8	−2.87 (−0.89)	−2.83 (−0.85)	4.350	4.14
InCl_4_^−^	−61.6	−62.2	−11.31 (−5.28)	−4.41 (1.62)	3.033	4.25
TlCl_4_^−^	−63.2	−63.4	−9.08 (−3.03)	−5.80 (0.25)	3.249	4.29
BBr_4_^−^	−73.7	−72.3	−2.63 (−0.50)	−2.61 (−0.48)	4.274	3.77
AlBr_4_^−^	−73.7	−72.7	−4.43 (−0.89)	−4.34 (−0.80)	4.355	4.11
GaBr_4_^−^	−72.6	−71.5	−4.84 (−1.04)	−4.73 (−0.93)	4.312	4.18
InBr_4_^−^	−63.0	−64.7	−6.06 (−2.52)	−5.37 (−0.64)	3.934	4.29
TlBr_4_^−^	−64.2	−65.7	−7.79 (−1.69)	−6.47 (−0.37)	3.722	4.33

## Data Availability

No new data were created or analyzed in this study.
